# Serum proteomics of adults with acute liver failure provides mechanistic insights and attractive prognostic biomarkers

**DOI:** 10.1016/j.jhepr.2025.101338

**Published:** 2025-01-30

**Authors:** Katharina Remih, Franziska-Maria Hufnagel, Anna Sophie Karl, Valerie Durkalski-Mauldin, William Martens Lee, Constantine J. Karvellas, Zemin Su, Jody A. Rule, Petra Tomanová, Laura Krieg, Isabel Karkossa, Kristin Schubert, Martin von Bergen, Frank Tacke, Sonja Luckhardt, Nicole Ziegler, Aimo Kannt, Bastian Engel, Richard Taubert, Robert John Fontana, Pavel Strnad, the US Acute Liver Failure Study Group

**Affiliations:** 1 Medical Clinic III, Gastroenterology, Metabolic Diseases and Intensive Care, University Hospital RWTH Aachen, Health Care Provider of the European Reference Network on Rare Liver Disorders (ERN RARE LIVER), Aachen, Germany; 2 Department of Public Health Sciences, Medical University of South Carolina, Charleston, SC, USA; 3 Department of Internal Medicine, Division of Digestive and Liver Diseases, UT Southwestern Medical Center, Dallas, TX, USA; 4 Department of Critical Care Medicine, University of Alberta, Edmonton, AB, Canada; 5 Department of Econometrics, Prague University of Economics and Business, Prague, Czechia; 6 Department of Molecular Systems Biology, Helmholtz Centre for Environmental Research, Leipzig, Germany; 7 Department of Hepatology and Gastroenterology, Charité-Universitätsmedizin, Campus Charité Mitte and Campus Virchow-Klinikum, Berlin, Germany; 8 Fraunhofer Institute for Translational Medicine and Pharmacology ITMP, Frankfurt am Main, Germany; 9 Goethe University, Institute of Clinical Pharmacology, Frankfurt am Main, Germany; 10 Department of Gastroenterology, Hepatology, Infectious Diseases and Endocrinology, Hannover Medical School, Hannover, Germany; 11 Department of Internal Medicine, Division of Gastroenterology, University of Michigan, Ann Arbor, MI, USA

**Keywords:** Acute liver injury, Proteomic profiling, Acetaminophen, ALF subtyping

## Abstract

**Background & Aims:**

Acute liver failure (ALF) is defined as rapid onset coagulopathy and encephalopathy in patients without a prior history of liver disease. We performed untargeted and targeted serum proteomics to delineate processes occurring in adult patients with ALF and to identify potential biomarkers.

**Methods:**

Sera of 319 adult patients with ALF (∼50% acetaminophen [APAP]-related cases) were randomly selected from admission samples of the multicenter USA Acute Liver Failure Study Group consortium and subdivided into discovery/validation cohorts. They were analyzed using untargeted proteomics with mass spectroscopy and a serum cytokine profiling and compared with 30 healthy controls. The primary clinical outcome was 21-day transplant-free survival. Single-cell RNAseq data mapped biomarkers to cells of origin; functional enrichment analysis provided mechanistic insights. Novel prognostic scores were compared with the model for end-stage liver disease and ALFSG prognostic index scores.

**Results:**

In the discovery cohort, 117 proteins differed between patients with ALF and healthy controls. There were 167 proteins associated with APAP-related ALF, with the majority being hepatocyte-derived. Three hepatocellular proteins (ALDOB, CAT, and PIGR) robustly and reproducibly discriminated APAP from non-APAP cases (AUROCs ∼0.9). In the discovery cohort, 37 proteins were related to 21-day outcome. The key processes associated with survival were acute-phase response and hepatocyte nuclear factor 1α signaling. SERPINA1 and LRG1 were the best individual discriminators of 21-day transplant-free survival in both cohorts. Two models of blood-based proteomic biomarkers outperformed the model for end-stage liver disease and ALFSG prognostic index and were reproduced in the validation cohort (AUROCs 0.83-0.86) for 21-day transplant-free survival.

**Conclusions:**

Proteomics and cytokine profiling identified new, reproducible biomarkers associated with APAP etiology and 21-day outcome. These biomarkers may improve prognostication and understanding of the etiopathogenesis of ALF but need to be independently validated.

**Impact and implications:**

Acute liver failure (ALF) is a sudden, and severe condition associated with high fatality. More sensitive and specific prognostic scores are urgently needed to facilitate decision-making regarding liver transplantation in patients with ALF. Our proteomic analysis uncovered marked differences between acetaminophen and non-acetaminophen-related ALF. The identification of routinely measurable biomarkers that are associated with 21-day transplant-free survival and the derivation of novel prognostic scores may facilitate clinical management as well as decisions for/against liver transplantation. Further studies are needed to quantify less abundant proteins. Although we used two cohorts, our findings still need to be independently and prospectively validated.

## Highlights

• Hepatocellular injury markers such as aldolase B reliably distinguish between APAP and non-APAP-related ALF.
• Acute-phase response and nuclear factor 1α signaling emerge as key processes associated with ALF outcome.
• Proteomic assessment improves the understanding of etiopathogenesis in ALF and identifies novel, biologically plausible biomarkers.

## Graphical abstract

**Figure:**
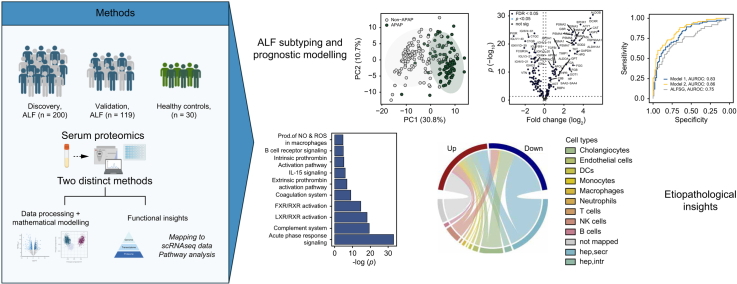

## Introduction

Acute liver failure (ALF) is a rare condition defined by a rapid loss of liver function within 26 weeks in the absence of pre-existing liver disease.^1^ In the USA, acetaminophen (APAP) toxicity is responsible for ∼50% of patients with ALF, whereas viral infection, other drug-induced liver injuries, autoimmune hepatitis, and indeterminate etiologies are other established causes.^1^ ALF is characterized by severe acute injury that can rapidly evolve into multi-organ failure. It is a common indication for urgent liver transplantation (LTX), but a decision for/against liver LTX is challenging because of its rapid development, scarcity of organs, and the need for life-long immunosuppression.^2^

To facilitate the clinical management of patients with ALF, several prognostic scores have been evaluated such as the model for end-stage liver disease (MELD) and the Acute Liver Failure Study Group Prognostic Index (ALFSG-PI). However, the former showed only a moderate ability to detect a potentially lethal ALF,^3^ whereas the ALFSG-PI performed less well in patients with favorable etiology and higher coma grades.^4^ Although there is a constant search for novel biomarkers, a better understanding of the complex changes occurring during ALF is essential for both prediction and treatment. Therefore, large-scale assessments such as the recently published miRNA panel^5^ are attractive approaches to improving the understanding of ALF pathogenesis and uncovering novel prognostic biomarkers. Serum proteomic analyses are equally promising since hepatocytes are responsible for the synthesis of the majority of serum proteins.^6^ In addition to changes in secreted proteins, hepatocyte injury is characterized by an increased release of various intracellular proteins such as aspartate/alanine aminotransferases (AST/ALT) that serve as liver injury markers.^6^ Moreover, the synthesis of liver-derived proteins is orchestrated by various liver-enriched transcription factors and adjusted to organismal needs. For example, the body responds to inflammation via the so-called acute-phase response (APR) that leads to increased production of acute-phase proteins (APPs) and diminished synthesis of anti-APP.^7^

Although several studies demonstrated that proteins indicative of hepatocellular injury such as cytokeratin 18 – M30 (M30)^8^ or hepatic synthesis of serum proteins such as factor V or hepcidin^1^^,^^9^ constitute attractive ALF prognostic markers, a large-scale analysis of proteomic changes occurring during ALF is currently lacking. Therefore, we performed untargeted serum proteomics of admission samples from a large, multicenter cohort of patients with ALF who had been prospectively enrolled in the ALFSG registry study. To account for the presence of inflammation and the corresponding changes in protein synthesis, a targeted cytokine profiling was carried out. The performance of assessed proteins was compared with the multivariable ALFSG-PI and MELD scores. Network analyses were undertaken to provide mechanistic insights.

## Materials and methods

### Patient cohort

The patients were randomly selected from a pool of 2,244 adult patients prospectively identified and recruited at 28 tertiary North American centers participating in the Acute Liver Failure Study Group (ALFSG) between 1999 and 2019. In total, 319 patients were randomly selected from the ALFSG registry and were subdivided into a discovery cohort (200 individuals) and validation cohort (119 individuals) by approximating the distribution of major ALF etiologies found in the overall registry (Table 1). All samples were drawn on Days 1 or 2 after admission to the hospital.

**Table 1:** Comparison of study cohorts and the overall ALFSG registry data.

Parameters	Discovery (n = 200)	Validation (n = 119)	Entire registry (N = 2,244)
Age, years	42 (31–53)	39 (32–51)	40 (24)
Females, n (%)	128 (64.0)	88 (74.0)	1,550 (69.1)
Body mass index	27 (24–32)	27 (23–31)	27.04 (6.62)
Diabetes, n (%)	34 (17.0)	22 (18.5)	372 (16.6)
Caucasians, n (%)	139 (69.5)	94 (79.0)	1,675 (74.6)
Ethnicity (% Hispanic or Latino)	19 (9.5)	9 (7.6)	209 (9.3)
21-day outcome			
Spontaneous survival, n (%)	100 (51.0)	57 (47.9)	1,059 (47.2)
Death, n (%)	51 (26.0)	31 (26.0)	693 (31.9)
LTX, n (%)	47 (24.0)	32 (27.0)	532 (23.8)

**Admission labs**			
ALT (IU/L)	2,024 (656–4,507)	1,865 (632–4,500)	1,898.5 (629, 4,411)
AST (IU/L)	1,783.5 (571–4,430)	1,809 (417–6,321)	1,561 (452, 4,961)
Alkaline phosphate (IU/L)	138 (104–189)	141 (107–198)	134 (99, 186.5)
Bilirubin (mg/dl)	8.16 (4–19)	7 (4–18)	7.3 (3.7, 19.2)
Creatinine (mg/dl)	1.3 (0.80–2.65)	1.40 (0.90–2.59)	1.6 (0.9, 3.0)
Hemoglobin (g/dl)	10.85 (9.58–16.60)	10.70 (9.50–12.40)	10.9 (9.5, 12.7)
INR	3 (2.20–4.98)	2.80 (2.05–4.40)	2.8 (2.0, 4.1)
MELD score	32.5 (26–38)	31 (26–37)	33.27 (26, 38)
Platelet count ( × 10^9^/L)	136 (86–195)	127 (73–198)	126 (83, 190)
Venous ammonia (μmol/L)	110 (70–186)	92 (63–129)	94.5 (62, 144)
Leukocyte count ( × 10^9^/L)	10 (6.5–14.2)	9.1 (6.5–13.9)	10.25 (6.7–14.8)
Clinical parameters at admission			
Admission HE grade 3/4, n (%)	93 (48.9)	58.00 (48.7)	1,030 (47.3)
Need for pressors, n (%)	46 (23.0)	19 (16.0)	464 (20.7)
Need for RRT, n (%)	50 (25.0)	24 (20.2)	505 (22.5)
Need for ventilator, n (%)	102 (51.0)	58 (48.7)	1,062 (47.4)
Etiology			
APAP, n (%)	95 (47.5)	66 (55.5)	989 (44.1)
Autoimmune, n (%)	28 (14.0)	17 (14.3)	159 (7.1)
Hepatitis B, n (%)	34 (17.0)	10 (8.4)	166 (7.4)
Indeterminate, n (%)	33 (16.5)	12 (10.1)	276 (12.3)
Other, n (%)	10 (5.0)	14 (11.8)	654 (29.1)

Data are expressed as median (25th-75th percentile) for continuous variables and n (%) for categorical variables. Admission HE grade 3/4, grade of hepatic encephalopathy at admission equal to or larger than 3.

ALT, alanine aminotransferase; APAP, acetaminophen; AST, aspartate aminotransferase; DILI, drug-induced liver injury; INR, international normalized ratio; LTX, liver transplantation; MELD, model for end-stage liver disease; pressors, vasopressors; RRT, renal replacement therapy.

All individuals met the previously defined ALF criteria.^1^^,^^8^^,^^9^ The selection of patients was carried out by ALFSG staff not directly involved in the project. The institutional review boards of all participating centers approved the research, and the clinical investigation was conducted according to the principles of the 1975 Declaration of Helsinki. As the patients with ALF present with an altered mental status, written informed consent was obtained from their legal next of kin. Monitoring and therapeutic interventions were according to local institutional standards of care. Demographic, clinical, laboratory, radiologic, and 21-day outcome data were recorded prospectively.

Sera of healthy controls were collected at the University Hospital Aachen (Germany) as part of the Alpha1 liver initiative as described.^10^ All cohorts were matched by age and sex.

### Pre-processing of serum proteomic datasets

Before processing the data, 716 proteins were identified in the discovery cohort, and 480 proteins in the validation cohort. All reverse and ‘only identified by site’ entries and potential contaminants were removed. The proteomic data were then log_2_-transformed and filtered for parameters reaching at least 70% valid values within at least one defined subgroup (healthy controls, APAP, or non-APAP cases of ALF). To further identify outliers, group-wise coefficients of variation were calculated and features with a coefficient of variation of maximum 0.3 within at least one defined subgroup were considered for downstream analysis (188 proteins in the discovery cohort and 137 proteins in the validation cohort). The remaining missing values were imputed column-wise with random draws from a distribution of the leftmost tail of the data (based on a quantile regression) [quantile regression imputation of left-censored data (QRILC) algorithm].^11^ Subsequently, the values were median-centered.

Differential abundance analysis for proteins and cytokines was performed by fitting the intensities in linear models with empirical Bayes moderation, no covariates were added. The *p* values were two-tailed and false discovery rate (FDR)-adjusted. Features were considered significantly differentially abundant across a given condition if the FDR was <0.05. Results are presented as volcano plots, plotting log_2_-fold changes on the x-axis and the obtained -log_10_ *p* values on the y-axis.

### Sample size calculation

Sample size estimation was based on prior human -omics studies.[12], [13], [14] Further, using statistical power analysis principles outlined by Cohen *et al.*,^15^ we estimated that at least 34 individuals in each subgroup are required to detect small effect sizes with a 0.8 statistical power at the 0.05 significance level. To further increase statistical power and adjust for multiple testing, we decided to include 200 individuals in the discovery cohort (sizes of subgroups: 100 spontaneous survivors, 98 death/LTX, two with missing information on outcome). The 2:1 split between the discovery and validation cohort is based on published recommendations.^16^

### Statistical analyses

All analyses were conducted using the R environment^17^ (R Foundation, Vienna, Austria, version 4.3.1) and R Studio^18^ (version 2023.06.1+524) using the following software packages: imputeLCMD,^19^ SummarizedExperiment,^20^ limma,^21^ ComplexHeatmap,^22^^,^^23^ circlize,^24^ glmnet,^25^^,^^26^ pROC,^27^ and corrplot.^28^ For the clinical data, continuous variables were displayed as median (IQR) and compared with the Wilcoxon rank-sum test, whereas categorical variables were presented as numbers (percentage), and comparison for proportion was done using the Χ^2^ test or Fisher’s exact test. Correlations between selected biomarkers were assessed in a pairwise manner using Spearman’s rank correlation test. Missing values in the clinical information were not imputed and not considered for any statistical calculations.

Principal component analysis (PCA) was carried out on the pre-processed proteomic data using the prcomp function with default parameters. Aiding the visualization, 95% CI ellipses were generated assuming a multivariate t-distribution.

Dot plots were used to display serum protein levels as determined via mass spectrometry across different conditions or etiologies. They display the median with first and third quartiles, their whiskers indicate the smallest and largest nonoutlier observations.

The ability of parameters to discriminate between APAP and non-APAP etiology or between 21-day transplant-free spontaneous survivors (SpS) *vs.* non-survivors (non-SpS) was assessed using the discovery cohort via univariate logistic regression models. For developing the multivariable models, all variables that differed between SpS and non-SpS were assessed, appropriate transformations were performed, and variables were analyzed for collinearity. Variables with an area under the receiver operating characteristic (AUROC) >0.55 and *p* value <0.05 in a univariable analysis were included. Variables with sample correlations greater than 0.7 or less than -0.7 were not included in the same model to mitigate collinearity and increase the stability and accuracy of estimated coefficients. Further, variable inflation factors were assessed for the selected models. Goodness of fit was assessed using the Hosmer–Lemeshow test. Performances of uni- and multivariable logistic regression models were evaluated using the AUROC, the DeLong test^29^ for correlated models, or the likelihood ratio test^30^ for nested models. The linearity between assessed variables and the logit of the 21-day outcome was assessed both graphically and using the Box–Tidwell test. Influential data points were assessed via Cook’s distance.

Data and results were reported using the TRIPOD reporting guidelines.^31^

For further methods, see the Supplementary information.

## Results

### Patients with ALF display substantial alterations in serum proteome

**Fig. 1:**
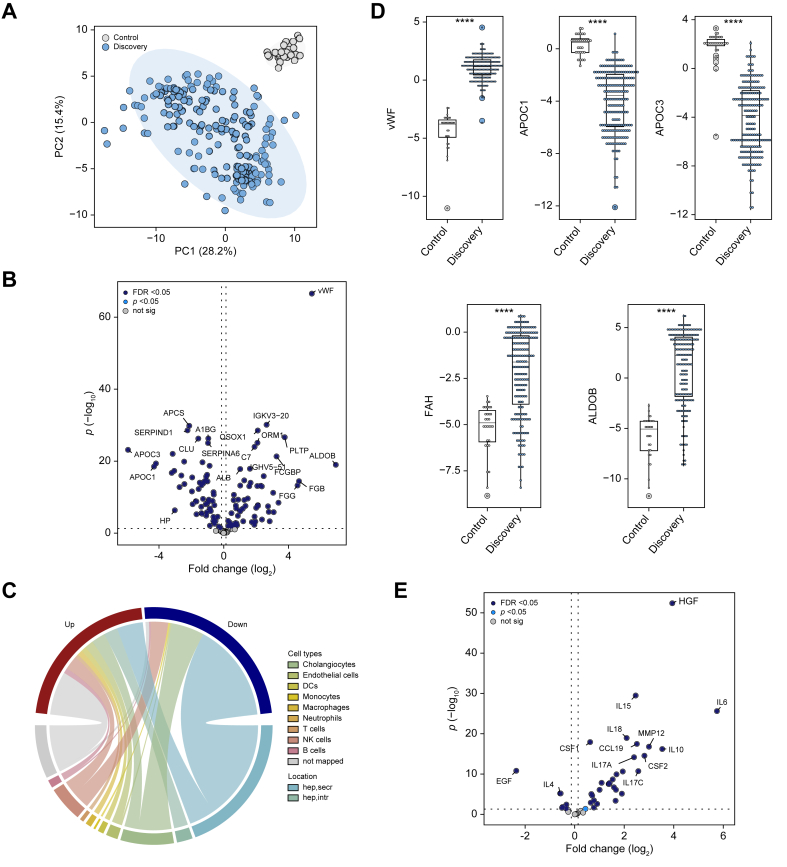
Serum proteomic alterations in patients with ALF *vs.* healthy controls. (A) The first two dimensions of the principal component analysis highlight marked differences in proteomic signatures of patients with ALF (discovery cohort) and healthy controls. (B) The corresponding volcano plot depicts the 187 differentially abundant proteins (98 elevated and 89 diminished in ALF, Bayesian linear regression). A log_2_ fold-change >0 indicates proteins elevated in patients with ALF *vs.* healthy controls (discovery cohort). (C) Chord diagram assigning the proteins altered between patients with ALF and healthy controls to publicly available liver single-cell RNAseq data (discovery cohort). Up/down refers to features increased/decreased in patients with ALF. (D) Serum levels of characteristic proteins that differ between patients with ALF and healthy controls, displayed via dot plots. Significance levels are as follows: ∗*p* <0.05, ∗∗*p* <0.01, ∗∗∗*p* <0.001 (Bayesian linear regression). (E) Volcano plot visualizing the results of the differential abundance analysis of a cytokine profiling comparing serum levels between patients with ALF and healthy controls (discovery cohort). The dotted horizontal lines in (B) and (E) depict an FDR <0.05. ALDOB, aldolase B; ALF, acute liver failure; APOC1, apolipoprotein C–I; APOC3, apolipoprotein C-III; FAH, fumarylacetoacetase, FDR, false discovery rate; NA, not available; not sig, features not reaching statistical significance in differential abundance analysis (*p* <0.05); vWF, von Willebrand factor.

We compared the serum proteome of 200 representative patients with ALF (discovery cohort) (Table 1) with 30 liver-healthy controls (Table S1). The PCA (Fig. 1A) revealed marked differences between the groups with 117 proteins (*i.e.* 82% of the identified proteome) being significantly altered (FDR <0.05, Fig. 1B, Table S2). Out of those, 57 were elevated and 60 were decreased in patients with ALF *vs.* healthy controls (FDR <0.05). A mapping to publicly available liver single-cell sequencing data (Fig. 1C, Table S2) revealed that most of the altered proteins were hepatocyte-derived (68%). The 60 decreased proteins were primarily secreted proteins (*e.g.* apolipoprotein C-III [APOC3] or apolipoprotein C–I [APOC1], Fig. 1C and D), likely reflecting a loss of synthetic function. In contrast, intracellular-derived proteins (*e.g.* aldolase B [ALDOB] or fumarylacetoacetase [FAH], Fig. 1C and D) were uniformly elevated in ALF, likely reflecting the massive hepatocyte injury. von Willebrand factor (vWF), an endothelial-cell-derived factor, was the protein that was most significantly increased in patients with ALF *vs.* controls (Fig. 1B and D).

Although the untargeted proteomics uncovered only a modest number of proteins related to immune cells, many were higher in patients with ALF compared with controls. A further assessment of inflammatory reaction with cytokine profiling revealed 36 elevated proteins (*i.e.* 80% of the 45 assessed markers) in patients with ALF (Fig. 1E). Among them, the hepatocyte growth factor (HGF), a potential surrogate of liver dysfunction,^32^ and the proinflammatory interleukins IL6 and IL15 were most profoundly altered (Table S3).

### Patients with APAP and non-APAP ALF have unique proteomic patterns

**Fig. 2:**
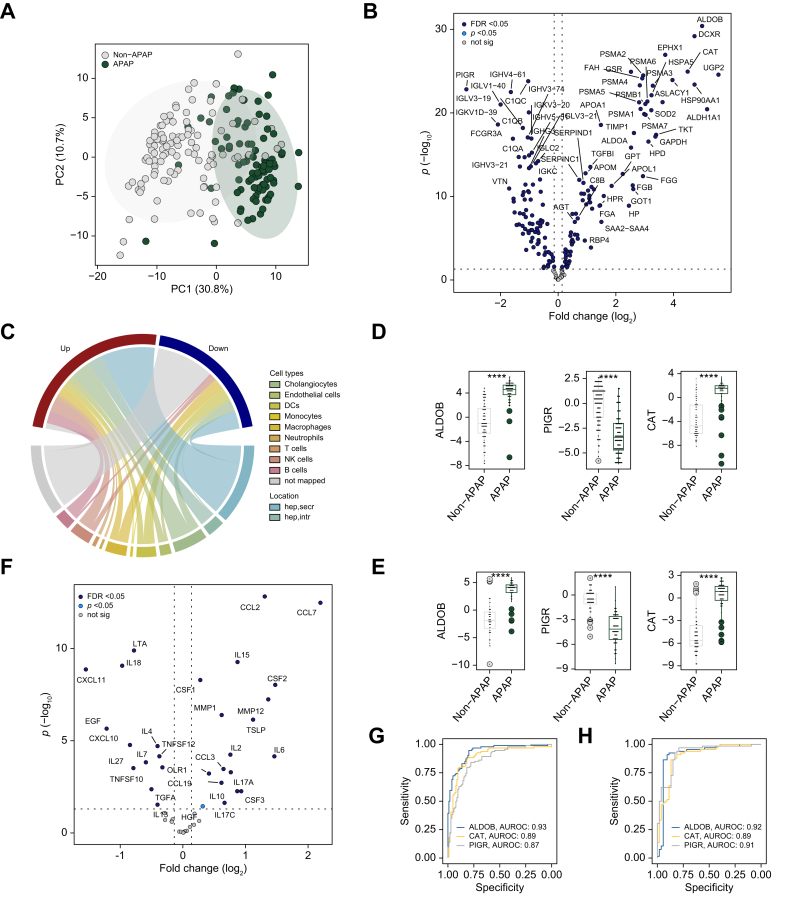
Serum proteomic alterations in patients with APAP *vs.* non-APAP ALF. (A) The first two dimensions of the principal component analysis highlight profound differences in proteomic signatures of patients with APAP and non-APAP ALF (discovery cohort). (B) The corresponding volcano plot depicts 167 differentially abundant proteins (83 elevated and 84 diminished in APAP; Bayesian linear regression). A log_2_ fold-change >0 indicates proteins elevated in APAP *vs.* non-APAP (discovery cohort). (C) Chord diagram representing the mapping of features altered between patients with APAP and non-APAP ALF to publicly available liver single-cell RNAseq data (discovery cohort). Up/down refers to features increased/decreased in patients with APAP ALF. (D/E) Serum levels of the top three discriminating features (ALDOB, CAT, and PIGR) are depicted via dot plots for both the discovery (D) and the validation (E) cohort. Significance levels are indicated as follows: ∗*p* <0.05, ∗∗*p* <0.01, ∗∗∗*p* <0.001 (Bayesian linear regression). (F) Volcano plot representing the results of the differential abundance analysis of the cytokine profiling between patients with APAP and non-APAP ALF (discovery cohort). Dotted horizontal lines in panels (B) and (F) depict FDR <0.05 (Bayesian linear regression). (G/H): Discriminative performances for the three proteins were assessed in univariable logistic regression models, results are depicted via receiver operating curves for both the discovery (G) and validation (H) cohort. ALDOB, aldolase B; ALF, acute liver failure; APAP, acetaminophen; APOC1, apolipoprotein C–I; APOC3, apolipoprotein C-III; FAH, fumarylacetoacetase; FDR, false discovery rate; NA, not available; not sig, features not reaching statistical significance in differential abundance analysis (*p* <0.05); vWF, von Willebrand factor.

When comparing patients with APAP *vs.* non-APAP as the major ALF etiologies, major differences in routine parameters (Table 2) and serum proteome (Fig. 2A) were noted. Among the 167 altered proteins, 47% were derived from hepatocytes including intracellular proteins (*e.g.* ALDOB or catalase [CAT]) likely reflecting the more pronounced hepatocellular injury of patients with APAP ALF (Fig. 2B–D, Table S4). In the cytokine profiling (Fig. 2E), patients with APAP ALF displayed increased levels of CC-chemokines (*i.e.* CCL2, CCL7), whereas tumor necrosis factor family and C-X-C family cytokines were typically diminished. Hepatocellular injury markers (ALDOB, CAT) were among the top discriminators between patients with APAP and non-APAP ALF, all reaching c-statistics of ∼0.9 in a univariable logistic regression model (Fig. 2F) thereby surpassing the abilities of routine parameters (c-statistics ≤0.86) and the assessed cytokines (c-statistics ≤0.81, Table S5). Notably, the major findings were reproducible in the validation cohort (Fig. 2D and F).

**Table 2:** Characteristics of patients with acetaminophen- and non-APAP-induced acute liver failure.

Variables	Discovery cohort				Validation cohort			
	n	APAP (n = 95)	Non-APAP (n = 105)	*p* value	n	APAP (n = 66)	Non-APAP (n = 53)	*p* value
Age, years	200	40 (31–51)	44 (31–56)	0.174	119	36 (28–42)	46 (37–57)	<0.001
Females, n (%)	200	64 (67.4)	64 (61.0)	0.345	119	55 (83.0)	33 (62.3)	0.009
Body mass index	189	27 (23–31)	28 (24–33)	0.123	119	25 (22–30)	29 (25–31)	0.037
Diabetes, n (%)	200	13 (13.7)	21 (20)	0.235	119	10 (15.2)	12 (22.6)	0.296
Caucasians, n (%)	200	74 (77.9)	65 (61.9)	<0.001	119	59 (89.3)	35 (66.0)	0.002
Ethnicity (% Hispanic or Latino)	200	9 (9.5)	10 (9.5)	0.990	119	2 (3.0)	7 (13.2)	0.076

**21-day outcome, n (%)**								
Spontaneous survival	198	49 (52.1)	51 (49.0)	0.664	119	37 (56.1)	20 (37.7)	0.047
Death	198	23 (24.0)	28 (27.0)	0.693	119	15 (23.0)	16 (30.0)	0.357
LTX	198	22 (23.0)	25 (24.0)	0.917	119	15 (23.0)	17 (32.0)	0.253

**Admission labs**								
ALT (IU/L)	193	3,532 (2,140–5,937)	847 (352–2,251)	<0.001	117	3,550 (2,177–6,406)	632 (311–1,458)	<0.001
AST (IU/L)	192	2,901 (1,792–7,784)	836 (326–1,914)	<0.001	118	4,717 (1,663–8,560)	522 (302–1,376)	<0.001
Alkaline phosphate (IU/L)	191	130 (101–183)	146 (110–203)	0.135	118	121 (104–160)	177 (124–226)	<0.001
Bilirubin (mg/dl)	192	5 (3–7)	18 (9–25)	<0.001	118	4 (3–6)	18 (13–25)	<0.001
Creatinine (mg/dl)	199	1.90 (1.00–3.00)	1.10 (0.70–2.03)	0.002	119	1.55 (1.00–2.90)	1.02 (0.70–2.40)	0.007
Hemoglobin (g/dl)	196	10.30 (8.45–11.88)	11.60 (9.95–13.08)	<0.001	117	10.60 (9.30–12.23)	11.50 (10.00–13.20)	0.063
INR	190	3.50 (2.40–5.30)	2.65 (2.10–4.60)	0.050	119	3.25 (2.30–4.95)	2.50 (2.00–3.40)	0.007
MELD score	184	32 (25–40)	33 (27–37)	0.807	118	32 (26–36)	30 (27–39)	0.970
Platelet count ( × 10^9^/L)	196	114 (65–166)	152 (107–220)	<0.001	117	32 (26–36)	30 (27–39)	0.360
Venous ammonia (μmol/L)	77	134 (77–269)	99 (61–140)	0.058	73	103 (73–139)	82 (53–124)	0.113
Leukocyte count ( × 10^9^/L)	196	9.8 (5.7–14.5)	10.2 (6.9–13.8)	0.74	117	9.5 (6.5–15.0)	8.9 (6.7–12.1)	0.14

**Clinical parameters at admission, n (%)**								
Admission HE grade 3/4	190	53 (56.7)	40 (41.2)	0.030	119	42 (63.6)	16 (30.2)	<0.001
Need for pressors	200	32 (33.7)	14 (13.3)	<0.001	119	13 (19.7)	6 (11.3)	0.215
Need for RRT	200	39 (41.1)	11 (10.5)	<0.001	119	14 (21.2)	10 (18.9)	0.751
Need for ventilator	200	62 (65.3)	40 (38.1)	<0.001	119	44 (66.7)	14 (26.4)	<0.001

**Etiology, n (%)**								
APAP		95 (100.0)	0 (0)			66 (100.0)	0 (0)	
Autoimmune		0 (0)	28 (26.7)			0 (0)	17 (32.1)	
Hepatitis B		0 (0)	34 (32.4)			0 (0)	10 (18.9)	
DILI		0 (0)	7 (6.7)			0 (0)	14 (26.4)	
Indeterminate		0 (0)	33 (31.4)			0 (0)	12 (22.6)	
Other		0 (0)	2 (1.9)			0 (0)	0 (0)	

Data are expressed as median (25th-75th percentile) for continuous variables and n (%) for categorical variables. The *p* values were calculated using the Wilcoxon rank-sum test for continuous variables or Fisher’s exact test for categorical variables. Admission HE grade 3/4, grade of hepatic encephalopathy at admission equal to or larger than 3.

ALT, alanine aminotransferase; APAP, acetaminophen; AST, aspartate aminotransferase; DILI, drug-induced liver injury; INR, international normalized ratio; LTX, liver transplantation; MELD, model for end-stage liver disease; RRT, renal replacement therapy.

### Admission levels of several serum proteins are associated with 21-day transplant-free survival

**Fig. 3:**
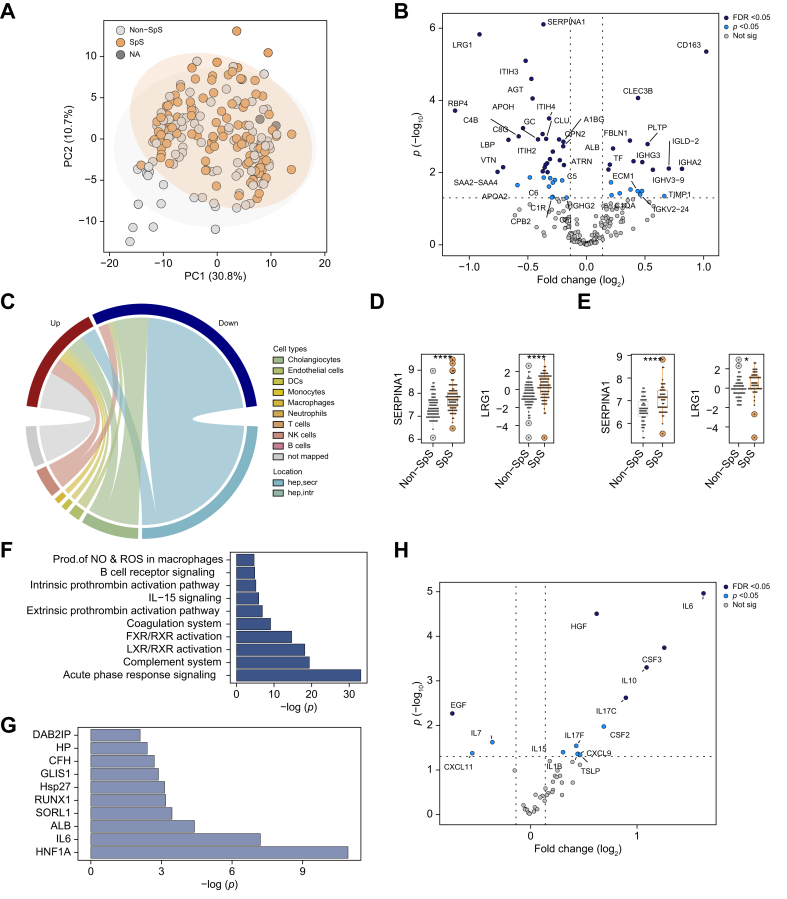
Serum proteomic alterations in patients with ALF with *vs.* without spontaneous survival. (A/B) The first two dimensions of the principal component analysis (A) show no obvious separation between patients passing away or receiving liver transplantation within 21 days after admission (non-SpS) and spontaneous survivors (SpS). A 39-protein signature (12 elevated and 27 diminished in SpS) discriminated both groups as seen in the corresponding volcano plot (B) (FDR <0.05, Bayesian linear regression). A log_2_ fold-change >0 indicates proteins elevated in non-SpS *vs.* SpS (discovery cohort). (C) The signature of altered proteins was mapped to publicly available liver single-cell RNAseq data (discovery cohort). (D/E) SERPINA1 and LRG1 are robustly associated with 21-day outcome in both discovery (D) and validation (E) cohort. Significance levels are indicated as follows: ∗*p* <0.05, ∗∗*p* <0.01, ∗∗∗*p* <0.001 (Bayesian linear regression). (F/G) Ingenuity Pathway Core Analysis (IPA) depicts 10 pathways (F) and upstream regulator networks (G) that were most significantly altered in the 21-day outcome dataset (full results, see Tables S9 and S10) (Fisher’s exact test) (discovery cohort). (H) Results of the cytokine profiling comparing non-SpS and SpS are depicted in a volcano plot; six features were differentially abundant (FDR <0.05, Bayesian linear regression) (discovery cohort). 21-day outcome, spontaneous survival *vs.* liver transplantation or death during the first 21 days post study admission; FDR, false discovery rate; hep, intr, hepatocellular intracellular features; hep, secr, hepatocellular secreted features; LRG1: leucine-rich alpha-2-glycoprotein; MELD: model for end-stage liver disease; NA, not available; not sig, features not reaching statistical significance in differential abundance analysis (*p* <0.05); SERPINA1: alpha1-antitrypsin.

In the discovery cohort, 51 patients (26%) died and 47 (24%) received LTX within 21 days after admission (non-SpS), while 100 patients (50%) were SpS. Patients who were non-SpS displayed higher international normalized ratio (INR), ammonia levels, hepatic encephalopathy grades, and MELD scores (Table 3). Although no obvious separation was detected between the SpS group and non-SpS group in the PCA (Fig. 3A), 39 proteins significantly differed between the groups (FDR <0.05, Fig. 3B). The majority of these proteins were secreted by hepatocytes (27; 69%) that were nearly uniformly decreased in patients who were non-SpS (Fig. 3C, Table S6), for example alpha1-antitrypsin (SERPINA1) or leucine-rich alpha-2-glycoprotein (LRG1) (Fig. 3D). In contrast, immune-related proteins such as scavenger receptor cysteine-rich type 1 protein M130 (CD163) or various immunoglobulin components were often elevated in patients who were non-SpS (Fig. 3B and C, Table S6).

**Table 3:** Characteristics of spontaneous survivors (SpS) and non-spontaneous survivors (non-SpS).

Variables	Discovery cohort				Validation cohort			
	n	SpS (n = 100)	Non-SpS (n = 98)	*p* value	n	SpS (n = 57)	Non-SpS (n = 62)	*p* value
Age	198	42 (30–53)	42 (32–56)	0.588	119	38 (31–52)	41 (32–49)	0.770
Sex (% female)	198	67 (68.4)	60 (60)	0.220	119	48 (84.2)	40 (64.5)	0.014
Body mass index (kg/m^2^)	187	27 (24–32)	27 (24–31)	0.805	119	28 (23–32)	26 (22–30)	0.557
Diabetes, n (%)	198	18 (18.4)	16 (16.0)	0.659	119	11 (19.3)	11 (17.7)	0.827
Caucasian race, n (%)	198	67 (68.4)	70 (70.0)	0.869	119	45 (79.0)	49 (79.0)	0.950
Hispanic/Latino ethnicity, n (%)	198	10 (10.2)	9 (9.0)	0.774	119	5 (8.8)	4 (6.5)	0.736

**Admission labs**								
ALT (IU/L)	191	1,911 (690–4,074)	2,179 (637–4,507)	0.961	117	2,333 (896–5,277)	1,630 (504–3,300)	0.218
AST (IU/L)	190	1,715 (544–2,893)	1,911 (594–5,515)	0.339	118	2,043 (538–6,550)	1,524 (375–4,769)	0.546
Alkaline phosphate (IU/L)	189	135 (102–203)	139 (106–174)	0.582	118	143 (105–207)	136 (110–193)	0.951
Bilirubin (mg/dl)	190	8 (4–17)	9 (5–22)	0.064	118	5 (3–10)	11 (6–20)	<0.001
Creatinine (mg/dl)	197	1.30 (0.70–2.65)	1.40 (0.90–2.75)	0.490	119	1.16 (0.80–2.50)	1.43 (0.90–2.60)	0.375
Hemoglobin (g/dl)	194	11.15 (9.90–12.80)	10.55 (9.08–12.30)	0.088	117	10.70 (9.50–12.20)	11.25 (9.48–12.40)	0.766
INR	188	2.60 (1.90–3.73)	3.95 (2.60–5.80)	<0.001	119	2.30 (1.90–4.20)	3.20 (2.50–5.08)	0.003
MELD score	182	29 (24–35)	35 (30–41)	<0.001	118	27 (23–33)	35 (30–39)	<0.001
Platelet count ( × 10^9^/L)	194	142 (92–204)	126 (75–189)	0.249	117	160 (104–220)	92 (64–165)	0.001
Venous ammonia (μmol/l)	77	92 (57–124)	144 (79–264)	0.004	73	92 (69–114)	97 (63–139)	0.544
Leukocyte count ( × 10^9^/L)	194	9.6 (5.8–14.4)	10.7 (7.1–14.1)	0.321	117	8.8 (6.5–13.7)	9.5 (6.5–14.1)	0.717

**Clinical parameters at admission, n (%)**								
Admission HE grade 3/4	188	34.0 (35.8)	57.0 (61.3)	<0.001	119	38 (66.7)	23. (37.1)	0.001
Need for pressors	198	16 (16.0)	29 (29.6)	0.023	119	2 (3.5)	17 (27.4)	<0.001
Need for RRT	198	22 (22.0)	28 (28.6)	0.287	119	7 (12.3)	17 (27.4)	0.04
Need for ventilator	198	38 (38.0)	63 (64.3)	<0.001	119	21 (36.8)	37 (59.7)	0.013

**Etiology, n (%)**								
APAP		49 (49.0)	45 (45.9)			37 (64.9)	29 (46.8)	
Autoimmune		13 (13.0)	15 (15.3)			8 (14.0)	9 (14.5)	
Hepatitis B		17 (17.0)	16 (16.3)			0 (0)	10 (16.1)	
DILI		4 (4.0)	2 (2.0)			9 (15.8)	5 (8.1)	
Indeterminate		17 (17.0)	16.0 (16.3)			3 (5.3)	9 (14.5)	
Other		0 (0)	3 (3.0)			0 (0)	0 (0)	

Data are expressed as median (25th-75th percentile) for continuous variables and n (%) for categorical variables. The *p* values were calculated using the Wilcoxon rank-sum test for continuous variables or Fisher’s exact test for categorical variables. Non-SpS and SpS refer to patients who died or required liver transplant within 21 days of enrolment and those who did not, respectively. Admission HE grade 3/4, grade of hepatic encephalopathy at admission equal to or larger than 3.

ALT, alanine aminotransferase; APAP, acetaminophen; AST, aspartate aminotransferase; Autoimmune, autoimmune hepatitis; DILI, drug-induced liver injury; INR, international normalized ratio; MELD: model for end-stage liver disease.

To account for the heterogeneity of our cohort regarding etiology, disease severity, and treatment modalities, the Bayes-corrected linear regression model was adjusted for different covariates (*e.g.* APAP yes/no, MELD, fulfillment of King’s College Criteria (KCC), the need for dialysis or vasopressor therapy at baseline). Notably, the adjusted models yielded comparable results and the most prominent predictors remained significant across all adjusted models (Fig. S1). To obtain further insights into the processes associated with 21-day outcome, we used Ingenuity Pathway Analysis (IPA, Qiagen, Hilden, Germany). The core analysis identified a significant (*p* <0.05) overlap with 36 pathways (Table S7), with the APR being the most significant (Fig. 3F). Hepatocyte nuclear factor 1A (HNF1A), a key liver-enriched transcription factor driving production of multiple secreted hepatocellular proteins and IL6, a master APR regulator were the top upstream regulators (Fig. 3G, Table S8). Notably, well-established IL6 targets include SERPINA1^6^ and LRG1.^33^ Accordingly, a targeted cytokine profiling revealed, that most proinflammatory cytokines were higher in non-SpS individuals and IL6 was the most significantly altered marker (Fig. 3H, Table S9).

**Fig. 4:**
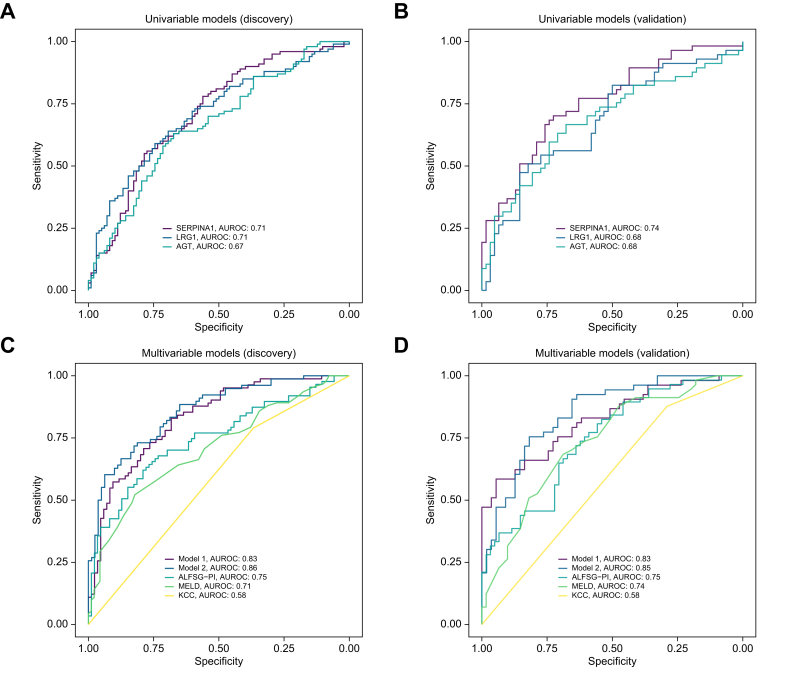
Ability of selected proteins and models to predict 21-day outcome in patients with ALF. (A/B) Areas under receiver operating curves (AUROCs) delineate the ability of the top three biomarkers to predict ALF outcome in the discovery (A) and validation (B) cohort (univariable logistic regression). (C/D) AUROCs for best-performing five-feature models (model 1 comprising SERPINA1, IL6, EGF, ATRN, and serum bilirubin; model 2 comprising SERPINA1, INR, the need for mechanical ventilation, EGF and serum bilirubin) are compared to the ALFSG-PI, the model for end-stage liver disease (MELD) and the King’s College Criteria (KCC) in the discovery (C) and validation (D) cohort (multivariable logistic regression). ALFSG-PI: ALFSG prognostic index; ATRN: attractin; EGF, epidermal growth factor; INR: international normalized ratio; SERPINA1: alpha1-antitrypsin.

SERPINA1 and LRG1 displayed a moderate correlation (*R*_*s*_ = 0.65, *p* <0.0001) whereas neither one markedly correlated with other established hepatic secretory proteins such as transthyretin or transferrin or their upstream regulators (*e.g.* IL6) (Fig. S2). LRG1, SERPINA1, and angiotensinogen (AGT) constituted the best discriminators for 21-day transplant-free survival with c-statistics ∼0.7 in a univariable logistic regression analysis that was similar in performance to the composite MELD score and was confirmed in the validation cohort (Fig. 4A–D, Table S10). In contrast, inflammation-related parameters did not display comparable discriminative properties with c-statistics ≤0.67 in the discovery cohort and an even worse performance in the validation cohort (Table S10).

**Fig. 5:**
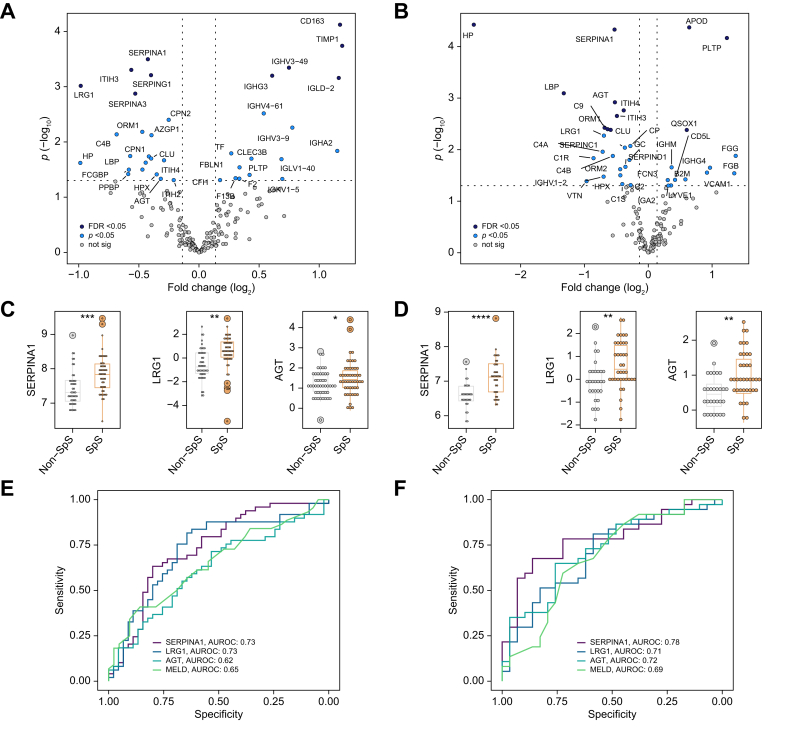
Proteins associated with spontaneous survival among patients with APAP-induced ALF. (A/B) Volcano plots depicting differential abundance analysis results in patients with APAP in the discovery (A) and validation cohort (B) (Bayesian linear regression). A log_2_ fold-change >0 indicates proteins elevated in non-SpS *vs.* SpS. (C/D) Dot plots visualizing protein levels of features associated with 21-day outcome (SERPINA1, LRG1, AGT) in APAP cases (discovery: C, validation: D). Significance levels are indicated as follows: ∗*p* <0.05, ∗∗*p* <0.01, ∗∗∗*p* <0.001 (Bayesian linear regression). (E/F) Predictive performances as determined via logistic regression (discovery: E, validation: F) of SERPINA1, LRG1, AGT, and MELD in patients with APAP. AGT, angiotensinogen; LRG1, leucine-rich alpha-2-glycoprotein; MELD, model for end-stage liver disease; not sig, features not reaching statistical significance in differential abundance analysis (*p* <0.05); SERPINA1, alpha1-antitrypsin.

As APAP is the leading etiology in both cohorts and clearly differs from patients with non-APAP ALF we investigated the behavior of the detected proteins in this more homogeneous subgroup. Although the volcano plots visualizing proteins that are significantly altered in patients who were SpS *vs.* non-SpS differed somewhat between the discovery and validation cohorts (Fig. 5A and B), SERPINA1 and LRG1 (Fig. 5C and D) emerged as the most robust prognostic markers reaching c-statistics 0.73/0.78 and 0.73/0.71 in the discovery and validation cohorts, respectively (Fig. 5E and F).

To predict the ALF course, we first assessed whether the addition of a proteomics-based parameter can improve the prognostic value of ALFSG-PI, however, only minor improvements were achieved (*e.g.* AUROC 0.77/0.79, *p* <0.0001 [likelihood ratio test] for SERPINA1 and 0.78/0.77, *p* <0.0001 [likelihood ratio test] for AGT in the discovery and validation cohort, respectively) (Table S11).

Next, we conducted a multivariable analysis that first considered all variables significantly associated with 21-day outcome in univariable analysis and displayed an AUROC >0.55 to be included in a potential model. We used the established ALFSG-PI score as a comparator and focused on the identification of alternative five-feature combinations. For model 1, we limited the search to continuous parameters. Among them, a combination of two secreted proteins (SERPINA1, attractin [ATRN]), two cytokines (IL6, epidermal growth factor [EGF]), and bilirubin displayed a reproducible performance (AUROCs 0.83 in both cohorts) numerically surpassing the AUROC of the ALFSG-PI score (AUROCs 0.75 in both cohorts), but the difference did not reach statistical significance. Consideration of categorical parameters yielded model 2 (encompassing SERPINA1, EGF, the INR, bilirubin, and the need for ventilation), which significantly improved the prediction (AUROC 0.86 in the discovery cohort and 0.85 in the validation cohort; *p* = 0.008, DeLong’s test; Fig. 4C and D; Table S12, Figs S3 and S4). Both models also outperformed the MELD score (AUROC 0.71 in the discovery cohort and 0.74 in the validation cohort) and the KCC (AUROCs 0.58 in both cohorts) (Fig. 4C and D).

## Discussion

In our study, we used two complementary proteomic techniques to analyze admission samples from a large multicenter observational cohort with a centralized study protocol.^1^^,^^8^^,^^9^ By mapping the evaluated proteins to the corresponding liver cell populations, we demonstrated an increase in several endothelial markers and a decrease in many secreted proteins. Among the former, vWF was the protein most significantly upregulated in patients with ALF *vs.* controls. This is in line with previous results, both in ALF^34^ and chronic liver disease,^35^ as well as the fact that its secretion is promoted by inflammation and endothelial damage.^34^^,^^35^ Regarding ALF etiologies, patients with APAP and non-APAP ALF displayed unique proteomic patterns and several hepatocellular injury markers constituted robust discriminators. This is not surprising as APAP constitutes the more acute ALF subtype displaying higher levels of routine hepatocellular damage markers.^1^ Among them, ALDOB was the best analyte to diagnose APAP ALF, and its usefulness was confirmed in the validation cohort. Our finding is in line with a recent proteomic study that uncovered ALDOB as a robust marker of hepatocellular drug-induced liver injury.^36^ Although ALT and ALDOB are both found in the cytoplasm and have a similar serum half-life,^37^ the latter is produced primarily in mid-lobule hepatocytes whereas ALT is more periportal.^36^ Because of that, ALDOB might be more sensitive to hepatocellular damage that occurs in the central area.^36^ Notably, a reliable detection of APAP-related ALF is of obvious clinical importance as its course can be ameliorated by timely administration of N-acetyl cysteine and the etiology might be difficult to determine because of impaired mental status or lack of cooperation.^1^^,^^2^

A combination of pathway analysis and single-cell mapping revealed a decrease in HNF1A-induced secreted proteins as a major indicator of poor prognosis. HNFs are key liver-enriched transcription factors orchestrating the synthesis of secreted hepatocellular proteins. In line with that, a defective expression of HNF4α was suggested to drive hepatocellular failure in alcoholic hepatitis,^38^ whereas its forced re-expression reversed experimental ALF.^38^ An impaired APR was the key process associated with poor prognosis. This is unsurprising as APR is believed to protect the body from stresses and is involved in microbial defense.^6^ For example, genetic inhibition of APR resulted in a significantly increased mortality in a mouse model of microbial infection.^39^ Among the assessed APPs, the levels of SERPINA1 and LRG1 were particularly good markers of a poor prognosis. The fact that serum levels of both proteins are poorly correlated with levels of their upstream regulators further suggests that the inability of the hepatocytes to adjust protein production to the organismal needs is linked to dismal outcomes.

This observation is biologically plausible as both proteins have multiple beneficial functions. For example, LRG1 promotes angiogenesis^40^ whereas SERPINA1 is a major protease inhibitor with anti-inflammatory and tissue-protective properties.^41^ Among the APR regulators, a strong overlap between the outcome dataset and IL6 signaling was revealed. This finding is in line with the important role of IL6 in liver regeneration and the demonstrated impaired regeneration of IL6-deficient mice.^42^^,^^43^

It is also consistent with a previous report that uncovered hepcidin, that is another well-established APP, as a good predictor of ALF outcome.^9^ In contrast to secreted proteins, cytokines and inflammatory proteins such as CD163 seem to be less robust indicators of prognosis but might be useful in disease subgroups or as adjuncts in prognostic scores.^44^^,^^45^ In our modeling effort, we obtained AUROC values comparable to or even superior to previously published models^4^^,^^8^ and in a direct comparison seemed to be even superior. In addition, model 1 relied only on continuous laboratory parameters, whereas the other score included variables such as coma grade that might be observer-dependent.

Although the mass spectrometry technique is robust and widely available, it misses less abundant proteins such as some intracellular proteins (e.g. lactate dehydrogenase or carbamoyl phosphate synthetase 1) which were previously suggested to be of prognostic relevance in ALF^46^^,^^47^ but were detected only in a subset of our patients (data not shown). Such proteins can be detected after depletion of the most abundant serum proteins,^46^ however, this approach is known also to introduce artifacts and is too laborious for routine use. A further limitation of our work is that we were not able to compare patients with ALF with patients who had acute liver injury without ALF, that we did not assess longitudinal samples, and were not able to fully account for temporal biases and changing treatments. Such analyses may yield important additional insights and warrant further studies.

In conclusion, our study assessed the unique ALFSG cohort to detect alterations in levels of serum proteins and cyto-/chemokines in individuals with ALF of different etiologies. These findings provide novel etiopathogenic insights and uncover attractive diagnostic biomarkers as well as predictors of disease outcome. The fact that several of the identified biomarkers (*i.e.* SERPINA1, IL6) are readily available in the clinical routine should facilitate their translation. Given the limited cohort size, our study was not sufficiently powered to develop and validate a novel clinically relevant score, but rather aimed at obtaining insight into the pathophysiology in ALF. Our findings will need to be validated in independent cohorts with different non-APAP ALF etiologies.

## Abbreviations

APOC1, apolipoprotein C–I; APOC3, apolipoprotein C-III; APP, acute-phase protein; APR, acute-phase response; AST, aspartate aminotransferase; ATRN, attractin; AUROC, area under the receiver operating curve; CAT, catalase; CCL2, C–C motif chemokine ligand 2; CCL7, C–C motif chemokine ligand 7; CD160, scavenger receptor cysteine-rich type 1 protein; CSF, colony-stimulating factor; DILI, drug-induced liver injury; EGF, epidermal growth factor; FAH, fumarylacetoacetase; FDR, false discovery rate; HGF, hepatocyte growth factor; HNF1A, hepatocyte nuclear factor 1A; INR, international normalized ratio; IPA, Ingenuity Pathway Analysis; KCC, King’s College Criteria; LRG1, leucine-rich alpha-2-glycoprotein; LTX, liver transplantation; MELD, model for end-stage liver disease; non-SpS, non-spontaneous survivors; PCA, principal component analysis; PIGR, polymeric immunoglobulin receptor; SERPINA1, alpha1-antitrypsin; SpS, 21-day transplant-free spontaneous survivors; vWF, von Willebrand factor.

## Financial support

PS is supported by the German Research Foundation (DFG) consortium SFB 1382 (ID 403224013) “Gut-liver axis” and DFG grant STR 1095/6-1 (Heisenberg professorship).

## Authors’ contributions

Conceptualization (lead): RJF, PS. Conceptualization (equal): KR. Visualization (lead): KR.

Visualization (supporting): ASK. Methodology (lead): PS. Data curation (lead): KR. Data curation (supporting): JAR. Formal analysis (lead): KR. Formal analysis (supporting): F-MH, PT. Writing – original draft (lead): KR. Writing – review and editing (lead): KR. Writing – review and editing (supporting): VD-M, WML, CJK, ZS, JAR, KS, MvB, FT, NZ, PS. Resources (lead): PS. Resources (equal): LK, IK, KS, MvB, SL, NZ, AK, BE, RT, RJF. Supervision (lead): PS. Funding acquisition (lead): PS. Full access to all the data and approved the final version of this manuscript: all authors.

## Data availability statement

Data are available upon reasonable request to the corresponding author.

## Conflicts of interest

PS reports receiving grant support and lecture fees from Grifols and CSL Behring, grant support and advisory board fees from Arrowhead Pharmaceuticals, grant support from Vertex Pharmaceuticals, advisory board fees from Dicerna Pharmaceuticals and Ono Pharmaceuticals, and lecture fees from Alnylam Pharmaceuticals. RJF has received research support from Kezar Pharmaceuticals, Takeda Pharmaceuticals, and the NIH (ALFSG and DILIN). WML consults for Genentech, SeaGen, GSK, and Veristat and receives research support from Gilead, Alexion, Vivet, Camurus, and Lipocine, none related to the current article. BE was supported by the PRACTIS – Clinician Scientist program of Hannover Medical School, funded by the German Research Foundation (DFG, ME 3696/3). All other authors report no conflicts of interest.

Please refer to the accompanying ICMJE disclosure forms for further details.
