# Supplementary material for: Serum proteomics of adults with acute liver failure provides mechanistic insights and attractive prognostic biomarkers
Source: JHEP Rep. 2025 Jan 30;7(5):101338. doi: 10.1016/j.jhepr.2025.101338 (PMC11998117; doi:10.1016/j.jhepr.2025.101338)
Supplement: Multimedia component 1 [file mmc1.pdf]

Serum proteomics of adults with acute liver failure provide mechanistic insights and attractive prognostic biomarkers

Katharina Remih, Franziska-Maria Hufnagel, Anna Sophie Karl, Valerie Durkalski-Mauldin, William Martens Lee, Constantine J. Karvellas, Zemin Su, Jody A. Rule, Petra Tomanová, Laura Krieg, Isabel Karkossa, Kristin Schubert, Martin von Bergen, Frank Tacke, Sonja Luckhardt, Nicole Ziegler, Aimo Kannt, Bastian Engel, Richard Taubert, Robert John Fontana, Pavel Strnad, and the US Acute Liver Failure Study Group

Table of contents

Supplementary Methods 2

Supplementary Tables 4

Supplementary Figures 31

Supplementary References 35

Supplementary Methods

Serum proteomics

Serum samples were diluted 1:10 in lysis buffer (8 M urea and 10 mM Dithiothreitol, both Sigma-Aldrich, Taufkirchen, Germany) and enzymatically cleaved with trypsin (Promega, Walldorf, Germany) using a paramagnetic bead approach as described previously [1]. Peptides were eluted in 2% dimethyl sulfoxide (Sigma-Aldrich, Taufkirchen, Germany), resulting in one fraction that was analysed by LC-MS/MS using an Ultimate 3000 nano ultra-performance liquid chromatography system (UPLC, Dionex, USA) coupled to a Q Exactive HF (Thermo Fisher Scientific, Waltham, USA). First, peptides were separated with a trapping column (flow rate 5ml/min, Acclaim PepMap 100 C18, 3μM, nanoViper, 75 μm x 5 cm, Thermo Scientific, Germany) and an analytical column (Acclaim PepMap 100 C18, 3 μm, nanoViper, 75 μm × 25 cm, Thermo Scientific, Germany) using a 80-minute non-linear gradient of hydrophilic solution A (0.1% formic acid (*v/v)* in ddH_2O) and hydrophobic solution B (80% acetonitrile (ACN; Merck, Darmstadt, Germany) and 0.1% ammonium formate (FA; Sigma Aldrich, Taufkirchen, Germany) in ddH_2O, *v/v*) as described previously [2]. The raw data were processed against the UniProtKB reference proteome of *Homo sapiens* (4 March 2022), using MaxQuant 1.6.2.10 default settings with label-free quantification and match between runs allowed.

Olink proteomics

Serum cytokine levels were analyzed with the proximity extension assay technology using the Olink® Target 48 Cytokine panel. Measurements were performed following the manufacturer’s instructions. Quality control parameters were applied and monitored as recommended by Olink®. A total of 45 proteins were reported in relative intensities (normalized protein expression, NPX). Measurements were conducted at the Fraunhofer Institute for Translational Medicine and Pharmacology (ITMP) in Frankfurt am Main (Germany).

Downstream analysis of serum proteomic datasets

The signatures of altered features were mapped to publicly available human single-cell RNAseq data of liver tissue. Guilliams et al performed scRNAseq and CITEseq on 19 human liver biopsies. Of these, most were histologically healthy, with only 5 patients showing >10% hepatic steatosis in the absence of any significant fibrosis [3,4]. The data was accessed via www.livercellatlas.org.

Functional annotation of regulated proteins and prediction of enriched pathways and their corresponding upstream regulators were performed using Ingenuity Pathway Analysis (IPA, Qiagen). The displayed p-values reflect the overlap between the dataset and the corresponding pathways and were calculated via Fisher's exact test.

Supplementary Tables

Supp. Table 1. Characteristics of healthy controls derived from the Aachen Alpha1-registry. Data are expressed as median (IQR) for continuous variables and n (%) for categorical variables. P-values are calculated by the Wilcoxon rank sum test for continuous variables or Fisher's exact test for categorical variables. *ALT: alanine aminotransferase; AST: aspartate aminotransferase; INR: international normalized ratio.*

	Controls (n = 30)		
**variables**	**Female (n = 22)**	**Male (n = 8)**	**p**
**Age**	27.0 (33.00)	45.5 (17.0)	0.1
**Body mass index**	22.1 (3.5)	25.5 (7.1)	0.06
**admission labs**			
**ALT (IU/L)**	19.5 (10.8)	31.0 (11.8)	0.02
**AST (IU/L)**	24.0 (9.3)	25.0 (10.0)	0.67
**Alkaline phosphate (IU/L)**	63.5 (29.0)	66.0 (18.0)	0.73
**Bilirubin (mg/dL)**	0.4 (0.2)	0.6 (0.1)	0.01
**Creatinine (mg/dL)**	0.8 (0.2)	1.0 (0.2)	0.005
**INR**	0.9 (0.1)	1.0(0.1)	0.11
**Platelet count (x10^9 / l)**	240.0 (101.0)	233.5 (89.0)	0.39

Supp. Table 2. Comparison of mean protein abundancies between acute liver failure (ALF) subjects [discovery cohort] and healthy controls. A log fold change > 0 indicates proteins elevated in ALF. Data are sorted according to false discovery rate adjusted p-value (FDR). Features with an FDR < 0.05 were mapped to publicly available single-cell RNAseq data (livercellatlas.org). For hepatocellular proteins, information on their localization (i.e., secreted vs. intracellular) was added. *logFC: log2 fold change; p: p-value; FDR: false discovery adjusted p-value uniprot: uniprot identifier; gene: gene symbol.*

logFC	p	FDR	uniprot	Gene	celltypes	secreted/intracellular
5.44	2.81274E-67	3.99409E-65	P04275	VWF	Endothelial cells	
2.64	7.72508E-31	5.4848E-29	P01619	IGKV3-20		
-2.13	1.53378E-30	7.25991E-29	P02743	APCS	Hepatocytes	secreted
-2.24	2.77013E-29	8.73294E-28	P05546	SERPIND1	Hepatocytes	secreted
2.1	3.07498E-29	8.73294E-28	O00391	QSOX1		
3.76	2.26288E-27	5.35548E-26	P55058	PLTP	Endothelial cells	
-0.95	4.97842E-27	9.45924E-26	P04217	A1BG	Hepatocytes	secreted

-1.57	5.32915E-27	9.45924E-26	P10909	CLU	Hepatocytes, Cholangiocytes	secreted
2.08	8.02039E-26	1.26544E-24	P02763	ORM1	Hepatocytes	secreted
-0.97	9.06211E-26	1.28682E-24	P08185	SERPINA6	Hepatocytes	secreted
1.9	1.04017E-24	1.34277E-23	P10643	C7		
-5.91	7.16213E-24	8.47519E-23	B0YIW2	APOC3	Hepatocytes, NK cells, Cholangiocytes	secreted
-3.16	9.33522E-23	1.01969E-21	P35858	IGFALS	Hepatocytes	secreted
3.26	4.8012E-22	4.86979E-21	Q9Y6R7	FCGBP		
-2.44	1.32855E-20	1.2577E-19	P02647	APOA1	Hepatocytes, NK cells, Cholangiocytes	secreted
-1.31	2.04803E-20	1.81763E-19	P19823	ITIH2	Hepatocytes	secreted
-4.19	4.4261E-20	3.69709E-19	K7ER74	APOC4-APOC2	Hepatocytes	secreted
6.92	1.01862E-19	8.03579E-19	P05062	ALDOB	Hepatocytes, NK cells, Cholangiocytes	intracellular
-0.87	1.95021E-19	1.45753E-18	P01031	C5	Hepatocytes	secreted
-4.31	3.17783E-19	2.25626E-18	K7ERI9	APOC1	Hepatocytes, NK cells	secreted
1.62	1.28611E-18	8.69652E-18	A0A0C4DH38	IGHV5-51		
1.02	1.5397E-18	9.93809E-18	CON__P02768-1	ALB	Hepatocytes, NK cells, Cholangiocytes	secreted
-3.06	4.88613E-18	3.01665E-17	V9GYM3	APOA2	Hepatocytes, NK cells, Cholangiocytes	secreted
-3.21	1.80225E-17	1.06633E-16	P04004	VTN	Hepatocytes	secreted
-1.03	6.17464E-17	3.5072E-16	G3XAM2	CFI	Hepatocytes	secreted
-2.62	9.70833E-17	5.30224E-16	P00747	PLG	Hepatocytes, NK cells, Cholangiocytes	secreted
2.47	1.45585E-16	7.65667E-16	P0DP06	IGHV4-30-4		
-1.09	1.86387E-16	9.45247E-16	P01024	C3	Hepatocytes, NK cells, Cholangiocytes	secreted
-1.95	3.30902E-16	1.62028E-15	P0C0L5	C4B	Hepatocytes	secreted
-1.11	1.97771E-15	9.36115E-15	A0A3B3ISJ1	PROS1	Hepatocytes	secreted
1.27	2.19559E-15	1.00572E-14	P19652	ORM2	Hepatocytes	secreted
4.65	3.70658E-15	1.6448E-14	P02675	FGB	Hepatocytes, NK cells, Cholangiocytes	secreted
-1	4.20391E-15	1.80896E-14	P19827	ITIH1	Hepatocytes, Cholangiocytes	secreted
-1.21	5.08607E-15	2.12418E-14	P22792	CPN2	Hepatocytes	secreted
-1.48	9.25382E-15	3.75441E-14	P07360	C8G	Hepatocytes	secreted

1.66	9.98269E-15	3.93762E-14	Q08380	LGALS3BP	Endothelial cells	
-1.26	1.28298E-14	4.92388E-14	P02749	APOH	Hepatocytes, Cholangiocytes	secreted
-2.43	2.29371E-14	8.57124E-14	P80108	GPLD1	Hepatocytes	secreted
-1.37	4.88575E-14	1.77891E-13	P07358	C8B	Hepatocytes	secreted
4.56	5.61152E-14	1.99209E-13	P02679	FGG	Hepatocytes	secreted
1.02	5.99555E-14	2.07651E-13	P0DOY2	IGHV3-30-3		
2.29	6.87383E-14	2.32401E-13	P01714	IGLV3-19		
2.07	7.93331E-14	2.61984E-13	P12955	PEPD	Hepatocytes	intracellular
1.58	9.08226E-14	2.93109E-13	A0A4W9A917	IGHG3		
2.35	1.02545E-13	3.23587E-13	P18428	LBP	Hepatocytes	secreted
-2.73	1.64868E-13	5.08939E-13	Q5VY30	RBP4	Hepatocytes, NK cells, Cholangiocytes	secreted
-1.55	5.59395E-13	1.69009E-12	P13671	C6	Hepatocytes, Cholangiocytes	secreted
-1.97	2.57395E-12	7.61459E-12	P29622	SERPINA4	Hepatocytes	secreted
-0.63	4.12404E-12	1.19513E-11	P01042	KNG1	Hepatocytes	secreted
3.05	6.08442E-12	1.72798E-11	P16930	FAH	Hepatocytes	intracellular
-0.62	1.94825E-11	5.42453E-11	P36955	SERPINF1	B cells, Hepatocytes	secreted
0.63	2.24501E-11	6.13061E-11	P00450	CP	Hepatocytes, NK cells, Cholangiocytes	secreted
1.48	2.37859E-10	6.37282E-10	P01717	IGLV3-25		
-1.28	3.01749E-10	7.93488E-10	P02774	GC	Hepatocytes, Cholangiocytes	secreted
0.89	4.97413E-10	1.26589E-09	P01857	IGHG1		
-0.92	4.99223E-10	1.26589E-09	P01008	SERPINC1	Hepatocytes	secreted
1.27	6.11076E-10	1.52233E-09	P01876	IGHA1		
0.61	6.71624E-10	1.64432E-09	P01023	A2M	Hepatocytes, Endothelial cells	secreted
-0.69	1.16459E-09	2.80292E-09	Q14624	ITIH4	Hepatocytes	secreted
-1.72	1.39691E-09	3.30601E-09	H0YAC1	KLKB1	Hepatocytes	secreted
-1.69	1.56504E-09	3.64322E-09	Q14520	HABP2	Hepatocytes	secreted
-0.94	1.61937E-09	3.70887E-09	P25311	AZGP1	Hepatocytes, Cholangiocytes	secreted
3.38	3.43927E-09	7.75201E-09	P04424	ASL	Hepatocytes	intracellular
-1.03	5.14803E-09	1.14222E-08	P09871	C1S	Hepatocytes, Cholangiocytes	secreted
-1.92	8.90424E-09	1.94523E-08	A0A096LPE2	SAA2-SAA4	Hepatocytes	secreted
-1.12	9.31018E-09	2.0031E-08	P07357	C8A	Hepatocytes	secreted
-0.58	1.73726E-08	3.64994E-08	B4E1Z4	CFB	Hepatocytes	secreted
2.11	1.74786E-08	3.64994E-08	P04432	IGKV1D-39		
0.75	2.1984E-08	4.52424E-08	P01834	IGKC		

2.12	2.52724E-08	5.12668E-08	P02671	FGA	Hepatocytes, NK cells, Cholangiocytes	secreted
1.34	2.77143E-08	5.54287E-08	A0A0A0MS15	IGHV3-49		
2.73	3.47816E-08	6.8597E-08	P01833	PIGR		
-1.39	4.70316E-08	9.14862E-08	P02750	LRG1	Hepatocytes	secreted
2	9.30015E-08	1.78462E-07	A0A0B4J1V1	IGHV3-21		
0.77	1.31467E-07	2.48911E-07	A0A087X1J7	GPX3		
-0.87	1.45728E-07	2.72281E-07	P04196	HRG	Hepatocytes	secreted
1.17	1.65106E-07	3.04482E-07	P01591	IGJ	B cells	
-0.83	3.85093E-07	7.01067E-07	P04003	C4BPA	Hepatocytes	secreted
-3.02	4.4699E-07	8.0345E-07	P00738	HP	Hepatocytes	secreted
1	5.05502E-07	8.97267E-07	P80748	IGLV3-21		
-0.8	7.41647E-07	1.30017E-06	C9JF17	APOD		
1.01	8.74756E-07	1.51482E-06	O43866	CD5L	Macrophages, DCs	
2.78	1.24682E-06	2.13311E-06	Q03154	ACY1	Hepatocytes	secreted
3.08	1.47859E-06	2.49952E-06	P04040	CAT	Hepatocytes, Erythroblasts, Platelets	intracellular
-1.04	1.6628E-06	2.77785E-06	P06727	APOA4		
0.48	2.8457E-06	4.69676E-06	P01009	SERPINA1	Hepatocytes	secreted
-1.38	2.87759E-06	4.69676E-06	P0C0L4	C4A	Hepatocytes	secreted
-1.34	4.01996E-06	6.48675E-06	P27169	PON1	Hepatocytes	secreted
-1.68	5.59654E-06	8.92931E-06	O14791	APOL1	Hepatocytes	secreted
-1.08	1.37298E-05	2.16626E-05	P02790	HPX	Hepatocytes, NK cells, Cholangiocytes	secreted
0.87	1.53258E-05	2.3915E-05	P02745	C1QA	Macrophages, DCs	
1.9	2.50458E-05	3.86577E-05	P24298	GPT	Hepatocytes	intracellular
-0.38	2.77541E-05	4.23773E-05	A0A7I2V2D2	SERPING1	Hepatocytes, Endothelial cells	secreted
-0.64	3.06918E-05	4.63643E-05	P00734	F2	Hepatocytes	secreted
2.36	0.00016456	0.000245974	Q7Z4W1	DCXR	Hepatocytes	intracellular
-0.55	0.000215421	0.000318643	A0A7P0T8D1	AGT	Hepatocytes, Cholangiocytes	secreted
0.27	0.000389032	0.00056951	P08603	CFH	Hepatocytes, NK cells, Cholangiocytes	secreted
-0.64	0.000394123	0.000571076	P02748	C9	Hepatocytes	secreted
-0.51	0.000408736	0.000586268	P04114	APOB	Hepatocytes, NK cells, Cholangiocytes	secreted
1.2	0.000507429	0.000713973	P01861	IGHG4		
2.84	0.000507826	0.000713973	P00352	ALDH1A1	Hepatocytes	intracellular
1.95	0.000604835	0.000842025	P32754	HPD	Hepatocytes, NK cells, Cholangiocytes	secreted

1.73	0.000847021	0.001167738	P29401	TKT	Macrophages, DCs	
-0.61	0.000880793	0.001202621	A0A3B3ISR2	C1R	Hepatocytes, Cholangiocytes	secreted
0.37	0.001136604	0.001537121	P51884	LUM		
0.73	0.00157569	0.00211083	P23142	FBLN1		
0.79	0.001848221	0.00245278	P63261	ACTG1	T cells, B cells	
-0.56	0.00247251	0.003250892	P02751	FN1	Hepatocytes, Cholangiocytes	secreted
0.58	0.003923617	0.005111501	P01871	IGHM		
1.89	0.004889676	0.006312127	P07900	HSP90AA1	T cells, B cells	
0.63	0.006067625	0.007762187	P04433	IGKV3D-11		
0.82	0.006259799	0.007936531	P01700	IGLV1-47		
-0.86	0.008284096	0.010410103	P07996	THBS1	Monocytes	
0.27	0.009787909	0.012191956	P02787	TF	Hepatocytes, NK cells, Cholangiocytes	secreted
0.45	0.012968705	0.016013532	O75636	FCN3	Hepatocytes, Endothelial cells	secreted
-0.31	0.014686437	0.017978224	P05543	SERPINA7	Hepatocytes	secreted
-0.2	0.03329387	0.040407945	P08697	SERPINF2	Hepatocytes, NK cells, Cholangiocytes	secreted
0.47	0.05276064	0.063491618	P06312	IGKV4-1		
0.42	0.085908649	0.102512841	P04211	IGLV7-43		
0.68	0.091692424	0.108502702	P04075	ALDOA		
-0.32	0.105516716	0.123829535	A0A087WT59	TTR		
0.27	0.133521433	0.155410192	A0A0A0MSV6	C1QB		
-0.29	0.162600301	0.187717421	P00748	F12		
0.19	0.19382011	0.221955287	P01011	SERPINA3		
-0.49	0.238088266	0.27046827	P01594	IGKV1-33		
-0.14	0.273265677	0.307966081	A0A4W8ZXM2	IGHV3-72		
-0.18	0.28042084	0.313541411	Q96PD5	PGLYRP2		
0.14	0.364173859	0.404005374	P02747	C1QC		
-0.15	0.368575218	0.405718457	A0A075B6S2	IGKV2D-29		
-0.08	0.464151906	0.504363894	P02765	AHSG		
0.1	0.465293452	0.504363894	A0A0B4J231	IGLL5		
0.21	0.540759058	0.581725654	A0A0B4J1U7	IGHV6-1		
0.15	0.567844233	0.603171365	P01624	IGKV3-15		
0.2	0.56918988	0.603171365	P00739	HPR		
0.08	0.580748575	0.607720984	P43652	AFM		
0.13	0.582042633	0.607720984	Q16610	ECM1		
0.15	0.750839596	0.778242501	P01602	IGKV1-5		
0.05	0.782133871	0.804804418	Q06033	ITIH3		
0.03	0.824597581	0.842394651	P02760	AMBP		

-0.04	0.839370993	0.851362008	P02649	APOE		
-0.02	0.867995359	0.874151354	P01859	IGHG2		
-0.01	0.91317593	0.91317593	O75882	ATRN		

Supp. Table 3. Comparison of mean cytokine levels between acute liver failure cases (ALF) [discovery cohort] and healthy controls. Data are sorted according to false discovery rate adjusted p-value (FDR). *logFC: fold change of the log-transformed cytokine abundancies.*

LogFC	p	FDR	uniprot ID	gene symbol
3.9400	4.12E-53	1.86E-51	P14210	HGF
2.4624	3.27E-30	7.35E-29	P40933	IL15
5.7436	2.42E-26	3.62E-25	P05231	IL6
2.0981	1.15E-19	1.29E-18	Q14116	IL18
0.6236	1.17E-18	1.06E-17	P09603	CSF1
2.5108	3.35E-18	2.51E-17	Q99731	CCL19
2.9987	1.64E-17	1.05E-16	P39900	MMP12
3.5357	6.21E-17	3.49E-16	P22301	IL10
2.8158	2.83E-15	1.41E-14	P04141	CSF2
2.3964	6.76E-15	3.04E-14	Q16552	IL17A
-2.3584	1.54E-11	6.32E-11	P01133	EGF
2.5691	1.93E-11	7.25E-11	Q9P0M4	IL17C
1.9404	2.33E-11	8.07E-11	Q07325	CXCL9
1.6882	1.18E-10	3.78E-10	P02778	CXCL10
1.5349	2.13E-09	6.39E-09	Q96PD4	IL17F
1.0868	1.49E-08	4.19E-08	P01375	TNF
1.4105	1.73E-08	4.58E-08	P13500	CCL2
1.3730	2.98E-08	7.45E-08	P10147	CCL3
1.3872	3.24E-08	7.66E-08	P60568	IL2
1.5857	1.96E-07	4.42E-07	Q969D9	TSLP
1.6667	8.13E-07	1.68E-06	P10145	CXCL8
1.0048	8.21E-07	1.68E-06	O95760	IL33
-0.5841	6.43E-06	1.21E-05	P05112	IL4
-0.5668	6.47E-06	1.21E-05	O43508	TNFSF12
1.9054	7.49E-06	1.35E-05	P09919	CSF3
0.6738	9.12E-06	1.58E-05	P51671	CCL11
0.7084	2.24E-05	3.73E-05	P03956	MMP1
0.7805	3.66E-04	5.88E-04	P48061	CXCL12
1.6512	4.29E-04	6.65E-04	P80098	CCL7
0.6484	1.28E-03	1.92E-03	P13236	CCL4
0.9066	2.53E-03	3.68E-03	Q8NEV9_Q14213	IL27
-0.3344	3.73E-03	5.25E-03	P50591	TNFSF10
-0.4887	1.48E-02	2.02E-02	P13232	IL7
0.7752	2.05E-02	2.71E-02	O14625	CXCL11
-0.5136	2.42E-02	3.12E-02	P13725	OSM

-0.3324	3.94E-02	4.93E-02	P01374	LTA
0.4348	4.30E-02	5.24E-02	P80075	CCL8
-0.2802	7.00E-02	8.29E-02	P49771	FLT3LG
0.2385	1.37E-01	1.59E-01	P78380	OLR1
-0.2600	2.39E-01	2.69E-01	P01135	TGFA
0.3380	3.71E-01	4.07E-01	P01579	IFNG
0.1157	5.63E-01	6.03E-01	Q99616	CCL13
0.0739	7.71E-01	8.07E-01	P35225	IL13
0.0398	8.90E-01	9.10E-01	P01584	IL1B
0.0004	9.98E-01	9.98E-01	P15692	VEGFA

Supp. Table 4. Comparison of mean protein abundancies between acetaminophen (APAP)- and non-APAP-induced acute liver failure cases [discovery cohort]. A log fold change > 0 indicates proteins elevated in APAP subjects. Data are sorted according to false discovery rate adjusted p-value (FDR). Features with an FDR < 0.05 were mapped to publicly available single cell RNAseq data (livercellatlas.org). For hepatocellular proteins, information on their behavior (i.e., secreted vs. intracellular) was added. *logFC: log fold change; p: p-value; FDR: false discovery adjusted p-value; uniprot: uniprot identifier; gene: gene symbol.*

logFC	P	FDR	uniprot	gene	celltypes	secreted/intracellular
4.99	3.97708E-31	7.47691E-29	P05062	ALDOB	Hepatocytes, NK cells, Cholangiocytes	intracellular
4.73	6.57044E-30	6.17621E-28	Q7Z4W1	DCXR	Hepatocytes	intracellular
3.71	1.12654E-27	7.05964E-26	P07099	EPHX1	Hepatocytes	intracellular
4.49	1.13721E-25	4.5237E-24	P04040	CAT	Hepatocytes, Erythroblasts, Platelets	intracellular
2.52	1.20311E-25	4.5237E-24	P00390	GSR		
5.56	2.65873E-25	8.33069E-24	Q16851	UGP2	T cells, Hepatocytes	intracellular
2.95	3.26205E-25	8.76093E-24	A0A024RA52	PSMA2		
2.92	7.38275E-25	1.73495E-23	P16930	FAH	Hepatocytes	intracellular
3.96	1.16774E-24	2.43928E-23	Q03154	ACY1	Hepatocytes	secreted
-1.04	1.61494E-24	3.03609E-23	P02747	C1QC	Macrophages, DCs	
4.73	4.07298E-24	6.9611E-23	P07900	HSP90AA1	T cells, B cells	
2.83	5.03868E-24	7.89392E-23	H0YMZ1	PSMA4	B cells	
3.28	5.55991E-24	8.04049E-23	P25788	PSMA3	Endothelial cells, B cells	
-3.18	1.5078E-23	2.02476E-22	P01833	PIGR		
-1.64	3.20525E-23	4.01725E-22	A0A0C4DH41	IGHV4-61		
3.24	7.65237E-23	8.99153E-22	P11021	HSPA5	B cells, Endothelial cells	
3.09	4.2939E-22	4.74855E-21	G3V5Z7	PSMA6		
3.62	5.18873E-22	5.33636E-21	P04424	ASL	Hepatocytes	intracellular
2.81	5.39313E-22	5.33636E-21	P28066	PSMA5	B cells	
3.02	8.58026E-22	8.06544E-21	P20618	PSMB1	Macrophages, DCs, Endothelial cells	
-1.99	9.93231E-22	8.89178E-21	P01703	IGLV1-40		
2.86	3.36758E-21	2.87775E-20	A0A7P0TAE1	HSP90B1	Macrophages, DCs, B cells	
5.16	3.63564E-21	2.97174E-20	P00352	ALDH1A1	Hepatocytes	intracellular
3.23	4.78887E-21	3.75128E-20	P04179	SOD2	Neutrophils	

-1.01	8.71818E-21	6.55607E-20	A0A0A0MSV6	C1QB	Macrophages, DCs	
2.99	1.3676E-20	9.88882E-20	P25786	PSMA1	Hepatocytes, B cells	intracellular
3.03	1.70671E-20	1.18837E-19	O14818	PSMA7	Macrophages, DCs, Endothelial cells	
-2.09	2.44909E-19	1.64439E-18	P04432	IGKV1D-39		
1.48	2.77914E-19	1.80165E-18	P02647	APOA1	Hepatocytes, NK cells, Cholangiocytes	secreted
-1.22	6.37517E-19	3.99511E-18	P01714	IGLV3-19		
2.63	2.54755E-18	1.54496E-17	Q5H9A7	TIMP1	Macrophages, DCs, Endothelial cells	
3.4	4.21454E-18	2.47605E-17	P29401	TKT	Macrophages, DCs	
3.37	6.86274E-18	3.90968E-17	P04406	GAPDH	B cells, Hepatocytes	intracellular
-1.05	8.70364E-18	4.8126E-17	A0A0B4J1X5	IGHV3-74		
-0.93	1.18836E-17	6.3832E-17	P01619	IGKV3-20		
-1.57	1.24301E-17	6.49125E-17	H0Y755	FCGR3A	Macrophages, DCs	
3.13	2.72222E-17	1.38318E-16	P32754	HPD	Hepatocytes, NK cells, Cholangiocytes	secreted
2.53	1.43129E-16	7.08114E-16	P04075	ALDOA	Monocytes	
-0.91	5.76981E-16	2.78135E-15	A0A0C4DH38	IGHV5-51		
-1.01	1.23161E-15	5.78857E-15	P02745	C1QA	Macrophages, DCs	
-1.37	1.9129E-15	8.77134E-15	P01624	IGKV3OR2-268		
-1.16	2.02407E-15	9.06013E-15	P01700	IGLV1-47		
-0.69	5.85995E-15	2.56203E-14	P0DOY2	IGLC2		
-0.77	1.07651E-14	4.59963E-14	P01857	IGHG1		
-0.95	2.5504E-14	1.0655E-13	P80748	IGLV3-21		
-1.32	2.64535E-14	1.08114E-13	A0A0B4J1V1	IGHV3-21		
1.12	2.94487E-14	1.17795E-13	Q15582	TGFBI	Macrophages, DCs, B cells	
-1.01	4.12618E-14	1.61609E-13	A0A4W9A917	IGHG3		
0.95	1.69827E-13	6.51581E-13	O95445	APOM	Hepatocytes	secreted
2.24	2.02712E-13	7.62197E-13	P24298	GPT	Hepatocytes	intracellular
2.94	3.78153E-13	1.39398E-12	P02679	FGG	Hepatocytes	secreted
-0.63	9.35607E-13	3.38258E-12	P01834	IGKC		
0.73	1.04673E-12	3.71294E-12	P01008	SERPINC1	Hepatocytes	secreted
0.88	2.38962E-12	8.31943E-12	P05546	SERPIND1	Hepatocytes	secreted
-1.13	3.83205E-12	1.2907E-11	A0A0A0MS15	IGHV3-49		
-0.87	3.84464E-12	1.2907E-11	P01717	IGLV3-25		
2.59	4.73263E-12	1.56094E-11	P02675	FGB	Hepatocytes, NK cells, Cholangiocytes	secreted

1.86	5.48507E-12	1.77792E-11	O14791	APOL1	Hepatocytes	secreted
1.16	7.25324E-12	2.3112E-11	I3L4N8	ACTG1	T cells, B cells	
-1.7	1.12756E-11	3.53302E-11	P04004	VTN	Hepatocytes	secreted
2.61	1.32726E-11	4.09058E-11	P17174	GOT1	Hepatocytes	intracellular
1.24	1.44095E-11	4.36935E-11	P27169	PON1	Hepatocytes	secreted
1.04	1.98244E-11	5.91585E-11	P12955	PEPD	Hepatocytes	intracellular
-1.04	2.398E-11	7.04413E-11	P06312	IGKV4-1		
-0.6	2.76716E-11	8.00348E-11	A0A0B4J231	IGLL5	B cells	
-1.19	3.03312E-11	8.63981E-11	P01599	IGKV1-17		
-1.01	3.14296E-11	8.81905E-11	A0A0C4DH67	IGKV1-8		
0.81	4.60121E-11	1.2721E-10	Q96PD5	PGLYRP2	Hepatocytes	secreted
0.9	4.87806E-11	1.3291E-10	A0A087WT59	TTR	Hepatocytes, NK cells, Cholangiocytes	secreted
-1.22	6.99576E-11	1.87886E-10	P01742	IGHV1-69		
1.08	8.12614E-11	2.15171E-10	H0YAC1	KLKB1	Hepatocytes	secreted
1.58	8.87559E-11	2.31751E-10	V9GYM3	APOA2	Hepatocytes, NK cells, Cholangiocytes	secreted
1.08	1.69503E-10	4.36527E-10	P29622	SERPINA4	Hepatocytes	secreted
-1.55	1.86652E-10	4.74198E-10	A0A0B4J1U7	IGHV6-1		
-1.16	2.38514E-10	5.94531E-10	Q15848	ADIPOQ		
-0.95	2.40342E-10	5.94531E-10	P01780	IGHV3-7		
0.85	2.87314E-10	7.01494E-10	P13671	C6	Hepatocytes, Cholangiocytes	secreted
-1.54	4.07776E-10	9.82845E-10	A0A0B4J1V2	IGHV2-26		
1.11	5.19482E-10	1.23624E-09	P02790	HPX	Hepatocytes, NK cells, Cholangiocytes	secreted
0.97	8.96334E-10	2.10639E-09	P55058	PLTP	Endothelial cells	
-0.53	9.84045E-10	2.28396E-09	A0A4W8ZXM2	IGHV3-72		
1.46	1.16616E-09	2.67363E-09	P00739	HPR	Hepatocytes	
2.45	1.2972E-09	2.91408E-09	P00738	HP	Hepatocytes	secreted
1.44	1.30203E-09	2.91408E-09	P02671	FGA	Hepatocytes, NK cells, Cholangiocytes	secreted
1.18	2.93864E-09	6.49958E-09	P18428	LBP	Hepatocytes	secreted
-1.11	4.29559E-09	9.39037E-09	P02775	PPBP		
-1.51	8.25175E-09	1.78314E-08	P04211	IGLV7-43		
-1.33	1.03813E-08	2.21783E-08	P01602	IGKV1-5		
0.62	1.17823E-08	2.48884E-08	P04196	HRG	Hepatocytes	secreted
0.5	1.25678E-08	2.62527E-08	P19827	ITIH1	Hepatocytes, Cholangiocytes	secreted
-1.2	1.27873E-08	2.64178E-08	P04433	IGKV3D-11		
-0.9	1.87555E-08	3.83264E-08	P01591	IGJ	B cells	
-1.12	2.98699E-08	6.03822E-08	P00747	PLG	Hepatocytes, NK cells, Cholangiocytes	secreted
0.68	4.27623E-08	8.55245E-08	P07358	C8B	Hepatocytes	secreted

-0.6	7.0474E-08	1.39464E-07	P23142	FBLN1		
-0.54	8.87896E-08	1.7388E-07	P01859	IGHG2		
1.5	1.11536E-07	2.16173E-07	A0A096LPE2	SAA2-SAA4	Hepatocytes	secreted
0.58	1.12903E-07	2.1659E-07	A0A7P0T8D1	AGT	Hepatocytes, Cholangiocytes	secreted
-0.56	1.32949E-07	2.52469E-07	P04114	APOB	Hepatocytes, NK cells, Cholangiocytes	secreted
-0.7	1.44855E-07	2.72328E-07	Q08380	LGALS3BP	Endothelial cells	
-0.98	1.72887E-07	3.21809E-07	Q9Y6R7	FCGBP		
-0.37	1.9678E-07	3.62692E-07	P05543	SERPINA7	Hepatocytes	secreted
-0.89	2.01815E-07	3.68361E-07	P0C0L5	C4B	Hepatocytes	secreted
-1.36	2.24229E-07	4.05337E-07	P02776	PF4		
-1.44	2.6349E-07	4.71773E-07	A0A0C4DH34	IGHV4-28		
-1.23	3.36227E-07	5.96326E-07	P01701	IGLV1-51		
-0.62	3.64442E-07	6.40328E-07	P02649	APOE	Macrophages, DCs, Hepatocytes	secreted
-0.74	6.7062E-07	1.16737E-06	P01876	IGHA1		
-1.14	7.9125E-07	1.36473E-06	A0A0B4J1Y8	IGLV9-49		
-0.93	1.05021E-06	1.79018E-06	P0C0L4	C4A	Hepatocytes	secreted
-0.9	1.05697E-06	1.79018E-06	A0A075B6K4	IGLV3-10		
-0.71	1.2796E-06	2.1479E-06	P01871	IGHM		
-1.03	1.48609E-06	2.47243E-06	P01782	IGHV3-9		
-0.8	1.81739E-06	2.9971E-06	P10643	C7		
0.44	2.0344E-06	3.3258E-06	P19823	ITIH2	Hepatocytes	secreted
-0.68	2.38445E-06	3.86445E-06	O43866	CD5L	Macrophages, DCs	
-1.02	2.40826E-06	3.86968E-06	A0A0C4DH68	IGKV2-24		
-1.22	2.9453E-06	4.6925E-06	A0A0J9YY99	IGLD-2		
0.58	3.70078E-06	5.84662E-06	P07360	C8G	Hepatocytes	secreted
0.67	4.07043E-06	6.377E-06	P07357	C8A	Hepatocytes	secreted
0.42	5.16907E-06	8.03128E-06	P43251	BTD		
-0.46	8.9495E-06	1.37107E-05	A0A087X1J7	GPX3		
-0.89	8.97033E-06	1.37107E-05	A0A182DWH7	SEPP1	Macrophages, DCs, Hepatocytes	
0.68	1.14606E-05	1.73758E-05	P02774	GC	Hepatocytes, Cholangiocytes	secreted
0.49	1.56852E-05	2.35906E-05	C9JF17	APOD		
-0.68	1.71454E-05	2.5582E-05	A0A075B6S2	IGKV2D-29		
0.92	1.91816E-05	2.83948E-05	P35858	IGFALS	Hepatocytes	secreted
-0.93	2.5001E-05	3.67203E-05	F5GZZ9	CD163	Macrophages, DCs, NK cells	
0.46	2.70301E-05	3.93927E-05	P01011	SERPINA3	Hepatocytes	secreted
0.52	2.81495E-05	4.07085E-05	P02763	ORM1	Hepatocytes	secreted
-0.68	3.22567E-05	4.62921E-05	P04003	C4BPA	Hepatocytes	secreted
-0.71	5.02305E-05	7.15404E-05	P20851	C4BPB	Hepatocytes	

0.48	5.60156E-05	7.918E-05	P25311	AZGP1	Hepatocytes, Cholangiocytes	secreted
-0.85	0.000121418	0.000170347	P01861	IGHG4		
-0.46	0.000130694	0.000181514	P09871	C1S	Hepatocytes, Cholangiocytes	secreted
1.14	0.000131308	0.000181514	Q5VY30	RBP4	Hepatocytes, NK cells, Cholangiocytes	secreted
0.26	0.000224148	0.00030759	P01042	KNG1	Hepatocytes	secreted
-1.11	0.000251884	0.000343146	A0A0G2JMB2	IGHA2		
0.33	0.000404603	0.000547233	P02760	AMBP	Hepatocytes, NK cells, Cholangiocytes	secreted
0.44	0.000410727	0.000551548	P00748	F12	Hepatocytes, NK cells, Cholangiocytes	secreted
-0.34	0.000459537	0.000612716	O00391	QSOX1		
-0.43	0.000487019	0.000644785	P19320	VCAM1	Macrophages, DCs	
-0.31	0.000508999	0.000666491	A0A3B3ISJ1	PROS1	Hepatocytes	secreted
0.24	0.000510504	0.000666491	P08185	SERPINA6	Hepatocytes	secreted
-0.68	0.000762196	0.000988227	Q16610	ECM1	Endothelial cells	
-0.23	0.000854832	0.001100742	P01023	A2M	Hepatocytes, Endothelial cells	secreted
-0.47	0.000980736	0.001254275	A0A3B3ISR2	C1R	Hepatocytes, Cholangiocytes	secreted
0.41	0.001168048	0.001483737	P02743	APCS	Hepatocytes	secreted
0.28	0.001208231	0.00152448	G3XAM2	CFI	Hepatocytes	secreted
-0.18	0.002050403	0.002569838	P08603	CFH	Hepatocytes, NK cells, Cholangiocytes	secreted
0.26	0.002425278	0.003019551	P01031	C5	Hepatocytes	secreted
-0.18	0.005084798	0.006289092	P00450	CP	Hepatocytes, NK cells, Cholangiocytes	secreted
-0.31	0.005372673	0.006601716	E9PHK0	CLEC3B		
0.39	0.005736855	0.007003433	P15169	CPN1	Hepatocytes	secreted
0.24	0.00674552	0.008181663	Q14624	ITIH4	Hepatocytes	secreted
0.32	0.007420557	0.008942722	P02749	APOH	Hepatocytes, Cholangiocytes	secreted
-0.28	0.007579048	0.009075548	P10909	CLU	Hepatocytes, Cholangiocytes	secreted
0.36	0.010908239	0.012979423	P13796	LCP1	Macrophages, Neutrophils, B cells	
-0.35	0.015659771	0.018515955	Q96RL7	VPS13A	Hepatocytes, Cholangiocytes	
0.27	0.020448688	0.024027209	Q06033	ITIH3	Hepatocytes, NK cells, Cholangiocytes	secreted
0.32	0.022296758	0.026035966	P02751	FN1	Hepatocytes, Cholangiocytes	secreted
-0.35	0.024358174	0.028267511	Q9Y5Y7	LYVE1	Macrophages, DCs, Hepatocytes	intracellular
-0.49	0.025453121	0.029356973	P07996	THBS1	Monocytes	
-0.14	0.025835578	0.029616394	P04217	A1BG	Hepatocytes	secreted
0.14	0.028639902	0.032632131	P22792	CPN2	Hepatocytes	secreted

0.31	0.029072167	0.032925104	P19652	ORM2	Hepatocytes	secreted
-0.51	0.036775887	0.0414004	A0A0C4DH43	IGHV2-70D		
-0.15	0.06730001	0.075311916	B4E1Z4	CFB		
0.14	0.074023305	0.082345451	P36955	SERPINF1		
-0.18	0.077915835	0.086165747	P01024	C3		
0.13	0.0977423	0.107459371	P01009	SERPINA1		
0.12	0.108667783	0.118776414	P08697	SERPINF2		
-0.12	0.119733716	0.130115253	P02765	AHSG		
0.17	0.162521433	0.175597871	P04275	VWF		
-0.09	0.223740041	0.24036073	O75882	ATRN		
0.18	0.245023676	0.261729836	P08571	CD14		
0.22	0.262761486	0.279091295	P02750	LRG1		
0.11	0.269027241	0.284141131	P05160	F13B		
0.1	0.347830509	0.365319194	P00734	F2		
-0.07	0.353076075	0.368768345	A0A7I2V2D2	SERPING1		
0.13	0.405115072	0.420782505	P06727	APOA4		
0.12	0.414305035	0.427963443	A0A087WSY5	CPB2		
-0.06	0.550208959	0.565241991	P43652	AFM		
0.06	0.613433623	0.626769137	P02748	C9		
-0.02	0.829390302	0.842839875	A0A0C4DGB6	ALB		
-0.01	0.924335988	0.928631374	P51884	LUM		
0.01	0.926771277	0.928631374	P02787	TF		
0.01	0.928631374	0.928631374	O75636	FCN3		

Supp. Table 5. Univariable logistic regression analysis modeling the probability of the acute liver failure case being related to acetaminophen or not. Model parameters obtained in the discovery cohort were then assessed in the validation cohort. Performances were evaluated via the c-statistic and are depicted for both discovery and validation cohort. Depicted are the 10 best-performing variables from the discovery cohort.*95 % CI: 95 % confidence interval; ALDOB: fructose-bisphosphate aldolase B; CAT: catalase; PIGR: polymeric immunoglobulin receptor; CCL7: CC-chemokine ligand 7; CCL2: CC-chemokine ligand 2; ALT: alanine aminotransferase; AST: aspartate aminotransferase; CXCL11: C-X-C motif chemokine 11; IL15: interleukin 15.*

	Discovery cohort			Validation cohort
**Variable**	**estimate**	**p**	**C-statistic (95%CI)**	**C-statistic (95%CI)**
ALDOB	0.84	1.23E-12	0.93 (0.89 - 0.96)	0.92 (0.85 - 0.98)
CAT	0.61	4.75E-14	0.9 (0.85 - 0.94)	0.89 (0.83 - 0.95)
Bilirubin	-0.23	3.46E-11	0.86 (0.8 - 0.91)	0.93 (0.87-0.98)
PIGR	-0.69	7.69E-14	0.86 (0.81 - 0.91)	0.92 (0.87 - 0.97)
CCL7	0.61	1.47E-09	0.81 (0.75 - 0.88)	0.88 (0.81 - 0.95)
CCL2	0.97	4.17E-10	0.8 (0.73 - 0.87)	0.88 (0.81 - 0.95)
ALT	0	5.4E-09	0.8 (0.74 - 0.86)	0.87 (0.81 - 0.94)
AST	0	5.59E-07	0.8 (0.74 - 0.86)	0.84 (0.77 - 0.92)
CXCL11	-0.59	6.58E-08	0.76 (0.69 - 0.83)	0.80 (0.7 - 0.9)
IL15	1.11	4.76E-08	0.76 (0.69 - 0.83)	0.88 (0.82 - 0.95)

Supp. Table 6. Comparison of mean protein abundancies between individuals surviving the first 21 days post study admission (SpS) and those who either received liver transplantation or died (non-SpS) [discovery cohort]. A log fold change > 0 indicates proteins elevated in the non-SpS group. Data are sorted according to false discovery rate adjusted p-value (FDR). Features with an FDR < 0.05 were mapped to publicly available single cell RNAseq data (livercellatlas.org). For hepatocellular proteins, information on their behavior (i.e., secreted vs. intracellular) was added. *logFC: log fold change; p: p-value; FDR: false discovery adjusted p-value; uniprot: uniprot identifier; gene: gene symbol.*

logFC	p	FDR	uniprot	Gene	Celltypes	secreted/intracellular
-0.37	7.8199E-07	0.00013929	P01009	SERPINA1	Hepatocytes	secreted
-0.91	1.4818E-06	0.00013929	P02750	LRG1	Hepatocytes	secreted
1.02	4.4435E-06	0.00027846	F5GZZ9	CD163	Macrophages, DCs, NK cells	
-0.52	7.9972E-06	0.00037587	Q06033	ITIH3	Hepatocytes, NK cells, Cholangiocytes	secreted
-0.47	2.5298E-05	0.00095121	A0A7P0T8D1	AGT	Hepatocytes, Cholangiocytes	secreted
0.44	8.5759E-05	0.00237305	E9PHK0	CLEC3B		
-0.46	8.8358E-05	0.00237305	P02749	APOH	Hepatocytes, Cholangiocytes	secreted
-1.12	0.00019195	0.0045108	Q5VY30	RBP4	Hepatocytes, NK cells, Cholangiocytes	secreted
-0.32	0.00031575	0.00659572	Q14624	ITIH4	Hepatocytes	secreted
-0.54	0.00058487	0.01099556	P02774	GC	Hepatocytes, Cholangiocytes	secreted
-0.37	0.00085422	0.01433652	P01011	SERPINA3	Hepatocytes	secreted
-0.58	0.00098634	0.01433652	P0C0L5	C4B	Hepatocytes	secreted
-0.34	0.00116213	0.01433652	P10909	CLU	Hepatocytes, Cholangiocytes	secreted
-0.41	0.00120042	0.01433652	P07360	C8G	Hepatocytes	secreted
-0.23	0.00120374	0.01433652	A0A7I2V2D2	SERPING1	Hepatocytes, Endothelial cells	secreted
-0.67	0.00123469	0.01433652	P18428	LBP	Hepatocytes	secreted
0.37	0.00129639	0.01433652	P23142	FBLN1		
-0.2	0.00139622	0.01458271	P04217	A1BG	Hepatocytes	secreted
0.52	0.00161907	0.01602023	P55058	PLTP	Endothelial cells	
-0.2	0.00186173	0.01750026	P22792	CPN2	Hepatocytes	secreted
0.23	0.00214728	0.01922325	A0A0C4DGB6	ALB	Hepatocytes, NK cells, Cholangiocytes	secreted

-0.29	0.00261528	0.02234874	P19823	ITIH2	Hepatocytes	secreted
-0.31	0.00422282	0.03451695	P01008	SERPINC1	Hepatocytes	secreted
-0.23	0.00452496	0.0354455	B4E1Z4	CFB	Hepatocytes	secreted
0.4	0.0048291	0.03631486	A0A4W9A917	IGHG3		
0.48	0.00510648	0.03692378	A0A0A0MS15	IGHV3-49		
-0.34	0.00550674	0.03834323	P25311	AZGP1	Hepatocytes, Cholangiocytes	secreted
0.2	0.00599917	0.03866879	P02787	TF	Hepatocytes, NK cells, Cholangiocytes	secreted
-0.35	0.00603139	0.03866879	P02763	ORM1	Hepatocytes	secreted
-0.19	0.00617055	0.03866879	P01042	KNG1	Hepatocytes	secreted
-0.71	0.0070687	0.04224382	P04004	VTN	Hepatocytes	secreted
-0.36	0.00719044	0.04224382	P05546	SERPIND1	Hepatocytes	secreted
0.7	0.00768664	0.04336761	A0A0J9YY99	IGLD-2		
0.82	0.00784308	0.04336761	A0A0G2JMB2	IGHA2		
0.19	0.00821402	0.04347611	O75882	ATRN	Hepatocytes, Cholangiocytes	secreted
0.57	0.00832521	0.04347611	P01782	IGHV3-9		
-0.37	0.00919998	0.04674583	P15169	CPN1	Hepatocytes	secreted
-0.76	0.00951609	0.04675962	A0A096LPE2	SAA2-SAA4	Hepatocytes	secreted
-0.33	0.00970013	0.04675962	P02743	APCS	Hepatocytes	secreted
-0.48	0.01358963	0.06295411	P0C0L4	C4A		
-0.36	0.01372935	0.06295411	P07357	C8A		
-0.31	0.0142004	0.0635637	P02748	C9		
-0.27	0.01597505	0.0698444	P04196	HRG		
-0.21	0.01639168	0.07003716	P01031	C5		
0.21	0.01858396	0.07728511	A0A4W8ZXM2	IGHV3-72		
-0.29	0.01891019	0.07728511	P09871	C1S		
-0.59	0.02212988	0.08851951	V9GYM3	APOA2		
-0.32	0.02430794	0.09520609	P13671	C6		
0.38	0.02930183	0.11242334	P10643	C7		
0.47	0.03228122	0.11980822	P07996	THBS1		
0.44	0.03250116	0.11980822	Q16610	ECM1		
0.28	0.03739679	0.13520379	P02745	C1QA		
0.46	0.04037614	0.14322103	A0A0C4DH68	IGKV2-24		
0.21	0.04224971	0.14709159	P01859	IGHG2		

0.66	0.04467688	0.15271371	Q5H9A7	TIMP1		
-0.29	0.04690918	0.15748082	A0A3B3ISR2	C1R		
-0.3	0.04864209	0.15913517	A0A087WSY5	CPB2		
-0.17	0.04909489	0.15913517	G3XAM2	CFI		
-0.25	0.05367646	0.16858831	P07358	C8B		
-0.19	0.05423359	0.16858831	P01024	C3		
0.4	0.05470153	0.16858831	P04433	IGKV3D-11		
0.35	0.05836482	0.1769772	A0A0C4DH41	IGHV4-61		
-0.23	0.06302342	0.18806988	P02649	APOE		
0.54	0.06465498	0.18992401	A0A0C4DH34	IGHV4-28		
-0.14	0.07089632	0.20357591	P08697	SERPINF2		
0.21	0.07146814	0.20357591	P02747	C1QC		
-0.48	0.07584067	0.21280664	P02776	PF4		
0.17	0.07961959	0.22012474	P43652	AFM		
0.33	0.0812826	0.22052632	A0A0B4J1V1	IGHV3-21		
0.26	0.08312012	0.22052632	O43866	CD5L		
0.14	0.08328388	0.22052632	O75636	FCN3		
0.57	0.08527283	0.2206671	P04075	ALDOA		
-0.16	0.08568457	0.2206671	P02760	AMBP		
0.2	0.08837893	0.22379092	P04275	VWF		
0.17	0.08927829	0.22379092	P51884	LUM		
0.17	0.09068842	0.22433452	O00391	QSOX1		
0.31	0.09557456	0.23335087	Q15848	ADIPOQ		
0.25	0.09684265	0.23341562	P01876	IGHA1		
0.26	0.10108278	0.24055142	P01780	IGHV3-7		
0.17	0.10391714	0.24187525	A0A087X1J7	GPX3		
-0.57	0.10535087	0.24187525	P20618	PSMB1		
-0.3	0.1063067	0.24187525	P02647	APOA1		
0.22	0.10678535	0.24187525	Q96RL7	VPS13A		
-0.2	0.11738272	0.2627137	P00748	F12		
-0.32	0.12192153	0.26966174	A0A182DWH7	SEPP1		
0.31	0.12874937	0.28145211	H0Y755	FCGR3A		
-0.24	0.13112876	0.2833587	Q15582	TGFBI		

-0.61	0.15133104	0.32329812	P00738	HP		
0.57	0.15548106	0.32838562	P02675	FGB		
0.26	0.15809437	0.32838562	P01624	IGKV3OR2-268		
0.08	0.16018645	0.32838562	P08603	CFH		
0.17	0.16069934	0.32838562	A0A0A0MSV6	C1QB		
-0.15	0.17134231	0.34636941	P04114	APOB		
0.18	0.17688396	0.35376792	A0A0B4J1X5	IGHV3-74		
0.14	0.18015416	0.35651561	P05160	F13B		
0.31	0.18363394	0.3577491	P01703	IGLV1-40		
-0.26	0.18458331	0.3577491	P02775	PPBP		
0.17	0.18764554	0.35997308	P19320	VCAM1		
0.21	0.19232165	0.36521686	A0A075B6S2	IGKV2D-29		
-0.19	0.19669726	0.36979085	A0A087WT59	TTR		
-0.08	0.19923243	0.37084849	P00450	CP		
0.52	0.20354954	0.37516974	P17174	GOT1		
-0.18	0.20719949	0.37818937	P02751	FN1		
0.3	0.21477852	0.38825349	P01602	IGKV1-5		
0.18	0.22387551	0.39826349	P01871	IGHM		
0.31	0.2246096	0.39826349	P04432	IGKV1D-39		
-0.17	0.22667124	0.39826349	O95445	APOM		
0.29	0.23180689	0.40082592	A0A0C4DH43	IGHV2-70D		
0.2	0.23239375	0.40082592	P04003	C4BPA		
0.12	0.24305357	0.41540064	P01857	IGHG1		
0.2	0.25699331	0.43526795	I3L4N8	ACTG1		
0.16	0.26180888	0.4394649	P13796	LCP1		
-0.15	0.2657811	0.44064027	P80748	IGLV3-21		
-0.08	0.26719676	0.44064027	P08185	SERPINA6		
0.13	0.29141385	0.4763983	A0A0C4DH38	IGHV5-51		
0.07	0.29887577	0.48438487	P01023	A2M		
0.2	0.30588871	0.49044631	P01599	IGKV1-17		
-0.15	0.30783332	0.49044631	P19652	ORM2		
-0.16	0.31803648	0.50111188	P08571	CD14		
-0.27	0.31985865	0.50111188	P04211	IGLV7-43		

-0.43	0.32316158	0.50210229	P29401	TKT		
0.15	0.33906189	0.51927318	A0A0C4DH67	IGKV1-8		
-0.18	0.33973724	0.51927318	P02790	HPX		
-0.18	0.34662934	0.52553481	P27169	PON1		
0.4	0.35863341	0.53938465	P02679	FGG		
-0.07	0.43365059	0.64359362	P19827	ITIH1		
0.18	0.43476803	0.64359362	P01861	IGHG4		
0.07	0.44704205	0.65647986	P01834	IGKC		
0.25	0.45045693	0.65647986	P24298	GPT		
-0.37	0.45456331	0.65736847	P04040	CAT		
-0.1	0.45932843	0.65918889	Q96PD5	PGLYRP2		
0.29	0.46894791	0.66789551	P32754	HPD		
-0.13	0.47286029	0.66840401	P29622	SERPINA4		
-0.14	0.48275761	0.67730172	P01742	IGHV1-69		
0.09	0.49701184	0.69213501	P01717	IGLV3-25		
-0.13	0.50864305	0.70312422	Q9Y6R7	FCGBP		
0.05	0.53985092	0.73702258	P05543	SERPINA7		
0.26	0.54242449	0.73702258	P04424	ASL		
0.09	0.55131767	0.73702258	Q9Y5Y7	LYVE1		
-0.06	0.55487952	0.73702258	P43251	BTD		
0.21	0.55624817	0.73702258	P01833	PIGR		
-0.36	0.55668727	0.73702258	Q16851	UGP2		
-0.15	0.5639339	0.74139562	A0A0B4J1V2	IGHV2-26		
-0.1	0.58264322	0.7606731	P20851	C4BPB		
0.13	0.60720846	0.78277589	A0A0B4J1U7	IGHV6-1		
0.08	0.60790043	0.78277589	P01700	IGLV1-47		
-0.15	0.63916873	0.81744028	P28066	PSMA5		
0.11	0.65040836	0.82179017	P01701	IGLV1-51		
0.07	0.65131242	0.82179017	P06312	IGKV4-1		
-0.12	0.67856417	0.8504671	O14791	APOL1		
0.07	0.69390145	0.86393028	P01591	IGJ		
0.14	0.71549389	0.88403685	P04179	SOD2		
-0.09	0.71945552	0.88403685	P02671	FGA		

-0.12	0.7318639	0.89060063	G3V5Z7	PSMA6		
-0.07	0.73851347	0.89060063	P35858	IGFALS		
-0.06	0.73900904	0.89060063	H0YAC1	KLKB1		
-0.03	0.75133555	0.89968843	P02765	AHSG		
-0.06	0.75900258	0.90311699	A0A075B6K4	IGLV3-10		
0.04	0.7833226	0.92619276	P06727	APOA4		
-0.11	0.78949081	0.9276517	P04406	GAPDH		
-0.08	0.79810403	0.93194756	A0A024RA52	PSMA2		
0.04	0.81896726	0.94499354	P12955	PEPD		
-0.06	0.8193295	0.94499354	P00390	GSR		
0.09	0.8292555	0.94583996	P07099	EPHX1		
-0.11	0.83505861	0.94583996	P05062	ALDOB		
-0.08	0.83515656	0.94583996	P25788	PSMA3		
0.04	0.85177524	0.95732224	P00747	PLG		
-0.06	0.85705316	0.95732224	P16930	FAH		
0.09	0.86057159	0.95732224	Q7Z4W1	DCXR		
0.01	0.87364115	0.95841957	A0A3B3ISJ1	PROS1		
0.07	0.87530925	0.95841957	Q03154	ACY1		
0.02	0.88206901	0.95841957	P01619	IGKV3-20		
0.07	0.8895085	0.95841957	P07900	HSP90AA1		
-0.01	0.89130167	0.95841957	A0A0B4J231	IGLL5		
-0.02	0.8935229	0.95841957	Q08380	LGALS3BP		
0.02	0.89724385	0.95841957	P01714	IGLV3-19		
-0.04	0.90258152	0.95867415	P25786	PSMA1		
0.04	0.91346737	0.96478576	A0A7P0TAE1	HSP90B1		
-0.05	0.93344943	0.97228725	P00352	ALDH1A1		
-0.01	0.94001698	0.97228725	P36955	SERPINF1		
-0.01	0.94974333	0.97228725	P0DOY2	IGLC2		
0.01	0.95528569	0.97228725	P00734	F2		
0.02	0.95574294	0.97228725	H0YMZ1	PSMA4		
0.02	0.95981711	0.97228725	O14818	PSMA7		
-0.01	0.96140765	0.97228725	A0A0B4J1Y8	IGLV9-49		
0.02	0.96194377	0.97228725	P11021	HSPA5		

0	0.98136807	0.98661602	C9JF17	APOD		
0	0.99442401	0.99442401	P00739	HPR		

Supp. Table 7. Prediction of canonical pathways to be related to the signature of serum proteins associated with 21-day outcome [discovery cohort]. Presented are all pathways that are significantly associated with the observed proteomic changes (-log10 of the overlap p-value < 1.3).

Ingenuity Canonical Pathways	-log(p)	Associated proteins in dataset
**Acute Phase Response Signaling**	33.10	AGT, AHSG, ALB, APCS, C1QA, C1QC, C1R, C4A/C4B, C4BPA, CFB, CRP, FGA, FGB, FGG, HRG, ITIH2, ITIH3, ITIH4, LBP, RBP4, SERPINA1, SERPINA3, SERPINF2, SERPING1
**Complement System**	19.40	C1QA, C1QC, C1R, C4A/C4B, C4BPA, C6, C8B, CFB, CFH, CFI, SERPING1
**LXR/RXR Activation**	18.20	AGT, AHSG, ALB, APOA4, C4A/C4B, CD14, CLU, FGA, ITIH4, LBP, PLTP, RBP4, SERPINA1, SERPINF2
**FXR/RXR Activation**	14.70	AGT, AHSG, ALB, APOA4, C4A/C4B, CLU, FGA, ITIH4, PLTP, RBP4, SERPINA1, SERPINF2
**Coagulation System**	9.09	FGA, FGB, FGG, SERPINA1, SERPINC1, SERPINF2
**Extrinsic Prothrombin Activation Pathway**	6.90	FGA, FGB, FGG, SERPINC1
**IL-15 Signaling**	5.94	IGHA2, IGHG3, IGHV1-69, IGHV3-72, IGHV6-1, IGKC, IGKV2D-29, IGKV4-1
**Intrinsic Prothrombin Activation Pathway**	5.18	FGA, FGB, FGG, SERPINC1
**B Cell Receptor Signaling**	4.88	IGHA2, IGHG3, IGHV1-69, IGHV3-72, IGHV6-1, IGKC, IGKV2D-29, IGKV4-1
**Production of Nitric Oxide and Reactive Oxygen Species in Macrophages**	4.70	ALB, APOA4, CAT, CLU, RBP4, SERPINA1
**Atherosclerosis Signaling**	4.44	ALB, APOA4, CLU, RBP4, SERPINA1
**IL-12 Signaling and Production in Macrophages**	4.31	ALB, APOA4, CLU, RBP4, SERPINA1
**Systemic Lupus Erythematosus In B Cell Signaling Pathway**	4.27	IGHA2, IGHG3, IGHV1-69, IGHV3-72, IGHV6-1, IGKC, IGKV2D-29, IGKV4-1
**Communication between Innate and Adaptive Immune Cells**	4.18	IGHA2, IGHG3, IGHV1-69, IGHV3-72, IGHV6-1, IGKC, IGKV2D-29, IGKV4-1
**Clathrin-mediated Endocytosis Signaling**	3.59	ALB, APOA4, CLU, RBP4, SERPINA1
**Maturity Onset Diabetes of Young (MODY) Signaling**	2.80	ADIPOQ, ALDOB, APOA4
**Multiple Sclerosis Signaling Pathway**	2.45	C1QA, C1QC, C6, C8B
**LPS/IL-1 Mediated Inhibition of RXR Function**	2.37	CAT, CD14, LBP, PLTP
**Neuroprotective Role of THOP1 in Alzheimer's Disease**	2.34	AGT, C1R, SERPINA3

**Role of Tissue Factor in Cancer**	2.31	FGA, FGB, FGG
**GP6 Signaling Pathway**	2.22	FGA, FGB, FGG
**IL-6 Signaling**	2.18	CD14, CRP, LBP
**iNOS Signaling**	2.08	CD14, LBP
**Primary Immunodeficiency Signaling**	2.00	IGHG3, IGKC
**Tyrosine Degradation I**	1.83	FAH
**Pathogen Induced Cytokine Storm Signaling Pathway**	1.73	CRP, FGA, FGB, FGG
**Hepatic Fibrosis / Hepatic Stellate Cell Activation**	1.72	AGT, CD14, LBP
**IL-10 Signaling**	1.71	CD14, LBP
**Macropinocytosis Signaling**	1.67	CD14, CSF1R
**Toll-like Receptor Signaling**	1.66	CD14, LBP
**Sucrose Degradation V (Mammalian)**	1.63	ALDOB
**Superoxide Radicals Degradation**	1.63	CAT
**LPS-stimulated MAPK Signaling**	1.58	CD14, LBP
**Apelin Adipocyte Signaling Pathway**	1.57	CAT, GPX3
**Systemic Lupus Erythematosus Signaling**	1.46	C6, C8B, IGHG3, IGKC
**Actin Cytoskeleton Signaling**	1.46	CD14, GSN, LBP

Supp. Table 8. Prediction of upstream regulators to be associated with signature of serum proteins associated with 21-day outcome [discovery cohort]. Presented are all upstream regulators that are significantly associated with the observed proteomic changes (-log10 of the overlap p-value < 1.3).

Upstream Regulator	-log(p)	target proteins in dataset
**HNF1A**	10.9281	ALB, APOH, CPB2, CRP, ITIH4, SERPINA1, SERPING1, VTN
**IL6**	7.2055	CD163, CRP, LBP, ORM1, SERPINA3
**ALB**	4.4056	AGT, ALB
**SORL1**	3.4486	AGT, AZGP1, FBLN1, PLTP
**RUNX1**	3.1798	ALB, CSF1R
**Hsp27**	3.1349	CD163, CSF1R
**GLIS1**	2.8729	EFEMP1, FBLN1
**CFH**	2.6968	CRP
**HP**	2.3958	CD163
**DAB2IP**	2.0958	CLU
**TCF**	2.0232	SERPINA1, SERPINA3
**IL17A**	1.9914	CD163, CRP
**Mmp**	1.9208	CSF1R
**TNF**	1.8928	AGT, CD163, CFB, CRP
**IFNG**	1.8729	AGT, CD163, CSF1R
**HFE**	1.8539	TF
**HSF1**	1.8327	EFEMP1, FBLN1

**TREX1**	1.8239	CFB, GSN
**STAT1**	1.7799	C1R, C4A/C4B
**TGFB1**	1.7545	ALB, CD163, SERPINA1
**CTNNB1**	1.7144	SERPINA1, SERPINA3
**Hemoglobin**	1.7011	CD163
**Pde4**	1.6596	CD163
**CG**	1.6478	C1R, C4A/C4B, CFB
**TNFSF11**	1.5884	CSF1R
**TXN**	1.5884	C5
**FOXA2**	1.5560	ALB
**IL1**	1.5258	CRP
**LEP**	1.5258	CRP
**C1Q (family)**	1.4989	CD163
**ATF3**	1.4989	GSN
**ARID1A**	1.4855	C4A/C4B, FBLN1
**EPO**	1.3830	TF
**PPARA**	1.3635	RBP4
**mir-155**	1.3261	CD163
**CSF1**	1.3089	CD163

Supp. Table 9. Comparison of mean cytokine levels between individuals surviving the first 21 days post study admission and those either receiving liver transplantation or passing away [discovery cohort]. Data are sorted according to false discovery rate adjusted p-value (FDR). *logFC: fold change of the log2-transformed cytokine abundancies.*

LogFC	p	FDR	uniprot ID	gene symbol
-1.6250	1.09E-05	4.89E-04	P05231	IL6
-0.6207	3.12E-05	7.01E-04	P14210	HGF
-1.2563	1.80E-04	2.71E-03	P09919	CSF3
-1.0899	4.99E-04	5.62E-03	P22301	IL10
-0.8949	2.40E-03	2.16E-02	Q9P0M4	IL17C
0.7356	5.40E-03	4.05E-02	P01133	EGF
-0.6874	1.06E-02	6.81E-02	P04141	CSF2
0.3593	2.38E-02	1.34E-01	P13232	IL7
-0.4287	2.87E-02	1.44E-01	Q96PD4	IL17F
-0.3049	3.99E-02	1.48E-01	P40933	IL15
0.5482	4.21E-02	1.48E-01	O14625	CXCL11
-0.4438	4.35E-02	1.48E-01	Q07325	CXCL9
-0.4413	4.37E-02	1.48E-01	P01584	IL1B
-0.4656	4.59E-02	1.48E-01	Q969D9	TSLP
-0.1802	6.31E-02	1.86E-01	O43508	TNFSF12
-0.3930	6.60E-02	1.86E-01	P10145	CXCL8
-0.4629	7.66E-02	2.03E-01	P39900	MMP12

-0.2524	1.02E-01	2.43E-01	P13236	CCL4
0.1496	1.03E-01	2.43E-01	P50591	TNFSF10
-0.2772	1.35E-01	2.89E-01	P10147	CCL3
-0.2286	1.35E-01	2.89E-01	O95760	IL33
-0.2453	1.44E-01	2.95E-01	P13725	OSM
-0.2101	1.84E-01	3.46E-01	Q99616	CCL13
-0.3958	1.92E-01	3.46E-01	P01579	IFNG
-0.2939	1.92E-01	3.46E-01	Q16552	IL17A
-0.1398	2.60E-01	4.50E-01	P78380	OLR1
-0.2097	2.81E-01	4.68E-01	P60568	IL2
-0.2038	3.14E-01	5.04E-01	P02778	CXCL10
-0.1657	3.32E-01	5.14E-01	P80075	CCL8
-0.0464	3.61E-01	5.14E-01	P09603	CSF1
-0.2955	3.65E-01	5.14E-01	P80098	CCL7
-0.1521	3.65E-01	5.14E-01	Q14116	IL18
-0.1113	3.81E-01	5.20E-01	P03956	MMP1
-0.0910	4.13E-01	5.46E-01	P51671	CCL11
-0.1318	4.92E-01	6.32E-01	P13500	CCL2
0.0665	6.20E-01	7.74E-01	P15692	VEGFA
-0.0393	6.88E-01	8.37E-01	P05112	IL4
0.0480	7.50E-01	8.59E-01	P01375	TNF
-0.0624	7.59E-01	8.59E-01	Q99731	CCL19
0.0538	7.64E-01	8.59E-01	P01135	TGFA
0.0331	7.89E-01	8.66E-01	P49771	FLT3LG
-0.0314	8.56E-01	9.17E-01	P48061	CXCL12
0.0224	9.05E-01	9.47E-01	P35225	IL13
0.0086	9.47E-01	9.62E-01	P01374	LTA
0.0108	9.62E-01	9.62E-01	Q8NEV9_Q14213	IL27

Supp. Table 10. Univariable logistic regression analysis modeling the probability of death or transplant within 21 days. Depicted are the three best-performing proteomic variables, several features derived from the cytokine profiling as well as routine clinical parameters. Model parameters obtained in the discovery cohort were then assessed in the validation cohort. Performances were evaluated via the c-statistic and are depicted for both discovery and validation cohort. *95 % CI: 95 % confidence interval; SERPINA1: alpha1 antitrypsin; MELD: model for end-stage liver disease; LRG1: leucine-rich alpha-2-glycoprotein; IL6: interleukin 6; AGT: angiotensinogen; HGF: hepatocyte growth factor; CSF3: colony stimulating factor 3; IL10: interleukin 10.*

	Discovery cohort			Validation cohort
**Variable**	**estimate**	**p**	**C-statistic (95%CI)**	**C-statistic (95%CI)**
SERPINA1	1.54	4.5E-06	0.71 (0.64 - 0.78)	0.76 (0.67 - 0.84)
MELD	-0.1	5.43E-06	0.71 (0.63 - 0.78)	0.74 (0.65 - 0.83)
LRG1	0.57	9.65E-06	0.71 (0.64 - 0.78)	0.68 (0.58 - 0.78)
IL6	-0.28	4.37E-05	0.67 (0.59 - 0.75)	0.62 (0.51 - 0.73)
AGT	0.84	7.49E-05	0.67 (0.59 - 0.74)	0.68 (0.59 - 0.78)
HGF	-0.71	8.81E-05	0.66 (0.58 - 0.74)	0.7 (0.6 - 0.81)
CSF3	-0.27	0.00047	0.65 (0.57 - 0.73)	0.57 (0.47 - 0.68)
IL10	-0.26	0.00092	0.64 (0.56 - 0.72)	0.45 (0.34 - 0.56)
Bilirubin	-0.03	0.061	0.58 (0.5 - 0.66)	0.72 (0.63 - 0.81)
Creatinine	-0.01	0.88	0.53 (0.45 - 0.61)	0.55 (0.44 - 0.65)

Supp.Table 11: Results of the multivariable logistic regression analysis combining the ALFSG-prognostic index (ALFSG-PI) and single proteomic features to model the probability of death or transplant within 21 days. For each model, results are presented via their respective areas under the receiver operating curve (AUROCs) in the discovery and validation cohort, as well as the difference between the two. Models numerically outperforming ALFSG-PI in both cohorts are highlighted. *SERPINA1: alpha1 antitrypsin; LRG1: leucine rich alpha-2-glycoprotein 1; IL6: interleukin 6; AGT: angiotensinogen; HGF: hepatocyte growth factor; CSF3: colony stimulating factor 3; IL10: interleukin 10.*

model	AUC (discovery)	AUC (validation)	AUC (validation) – AUC (discovery)
**ALFSG-PI**	0.7465	0.746	-0.0005
**SERPINA1 + ALFSG-PI**	0.7681	0.8001	0.032
**LRG1 + ALFSG-PI**	0.7839	0.7475	-0.0364
**IL6 + ALFSG-PI**	0.7418	0.7345	-0.0073
**AGT + ALFSG-PI**	0.78	0.7659	-0.0141
**HGF + ALFSG-PI**	0.7478	0.7633	0.0155
**CSF3 + ALFSG-PI**	0.7512	0.7331	-0.0181
**IL10 + ALFSG-PI**	0.7544	0.7328	-0.0217

Supp. Table 12. Composition of the selected prognostic models (modeling probability of death or liver transplant within 21 days). *SERPINA1: alpha1 antitrypsin; IL6: interleukin 6; EGF: epidermal growth factor; ATRN: attractin; INR: international normalized ratio; HE grade: degree of hepatic encephalopathy; VIF: variable inflation factor.*

	estimate	standard error	z value	p	VIF
**Model 1**					
(Intercept)	-10.4835	3.8682	-2.7102	0.0067	
**SERPINA1**	1.6242	0.4188	3.8783	0.0001	1.0642
**IL6**	-0.3735	0.0955	-3.9098	0.0001	1.2924
**EGF**	0.2892	0.128	2.2599	0.0238	1.2775
**ATRN**	-0.8711	0.4413	-1.9737	0.0484	1.086
**Bilirubin**	-0.0719	0.0218	-3.2987	0.001	1.2573
**Model 2**					
	**estimate**	**standard error**	**z value**	**p**	**VIF**
(Intercept)	-8.8775	4.0165	-2.2102	0.0271	
**SERPINA1**	1.0815	0.4454	2.428	0.0152	1.0566
**INR**	-0.4147	0.1275	-3.2539	0.0011	1.1953
**need for ventilation (yes)**	-1.7583	0.467	-3.7654	0.0002	1.3317
**EGF**	0.4016	0.1387	2.8964	0.0038	1.263
**Bilirubin**	-0.1041	0.0263	-3.9631	0.0001	1.6811
**ALFSG-PI**					
	**estimate**	**standard error**	**z value**	**p**	**VIF**
(Intercept)	3.9343	0.8916	4.4127	0.00001	
**HE grade (3/4)**	-1.2381	0.3691	-3.3547	0.0008	1.1145
**favorable etiology (yes)**	-0.0944	0.4571	-0.2065	0.8364	1.7045
**need for vasopressor therapy (yes)**	-0.3198	0.4656	-0.6867	0.4923	1.1647
**ln(Bilirubin)**	-0.6274	0.2465	-2.5448	0.0109	1.6779
**ln(INR)**	-1.5322	0.3733	-4.1042	0.00004	1.0696

Supplementary Figures

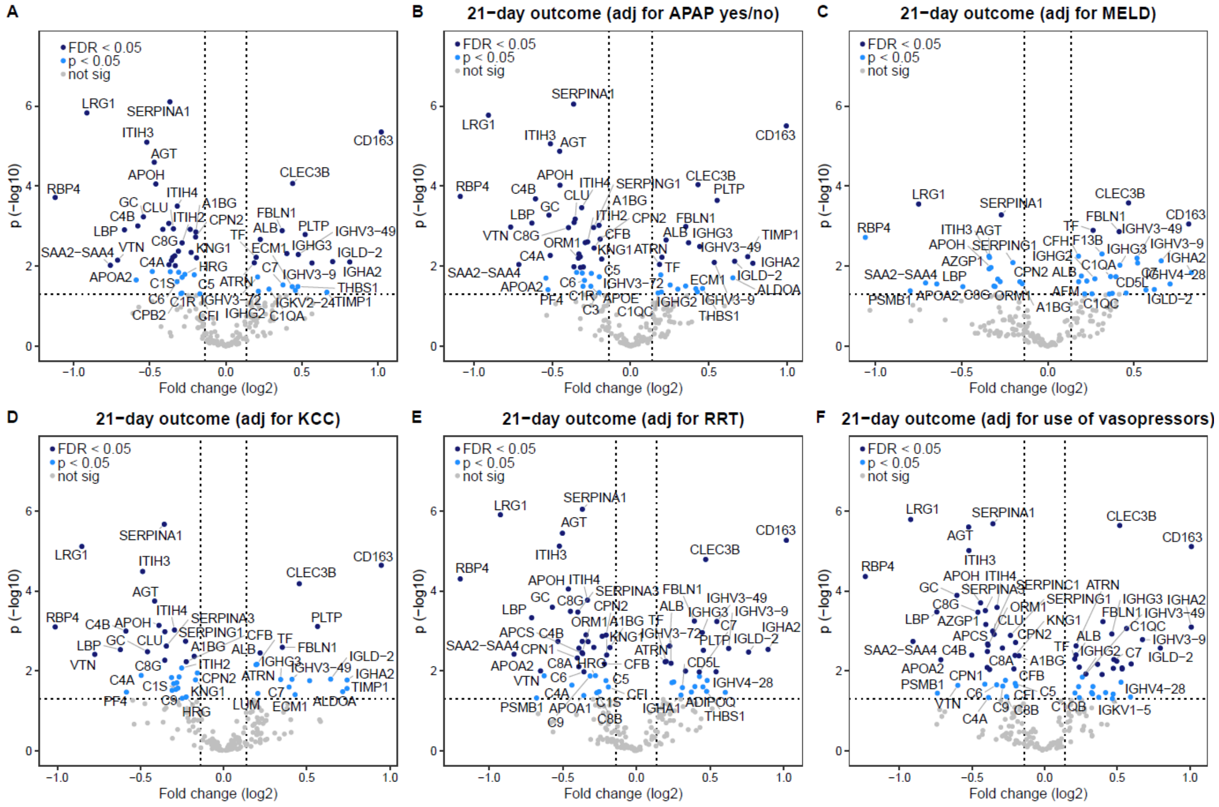

Supp. Fig. 1: Adjustment of 21-day outcome differential abundance analysis for different parameters of disease progression/severity. Volcano plots depicting proteins that are differentially abundant between spontaneous survivors and non-survivors are presented in the original form as stated in Figure 3 of the main manuscript (no covariates added) [A] and after the addition of covariates representing either disease etiology (APAP yes/no) [B] or surrogates of disease severity (MELD [C], King's College Criteria [KCC] [D], need for renal replacement therapy [RRT] [E] or need for the use of vasopressors [F].

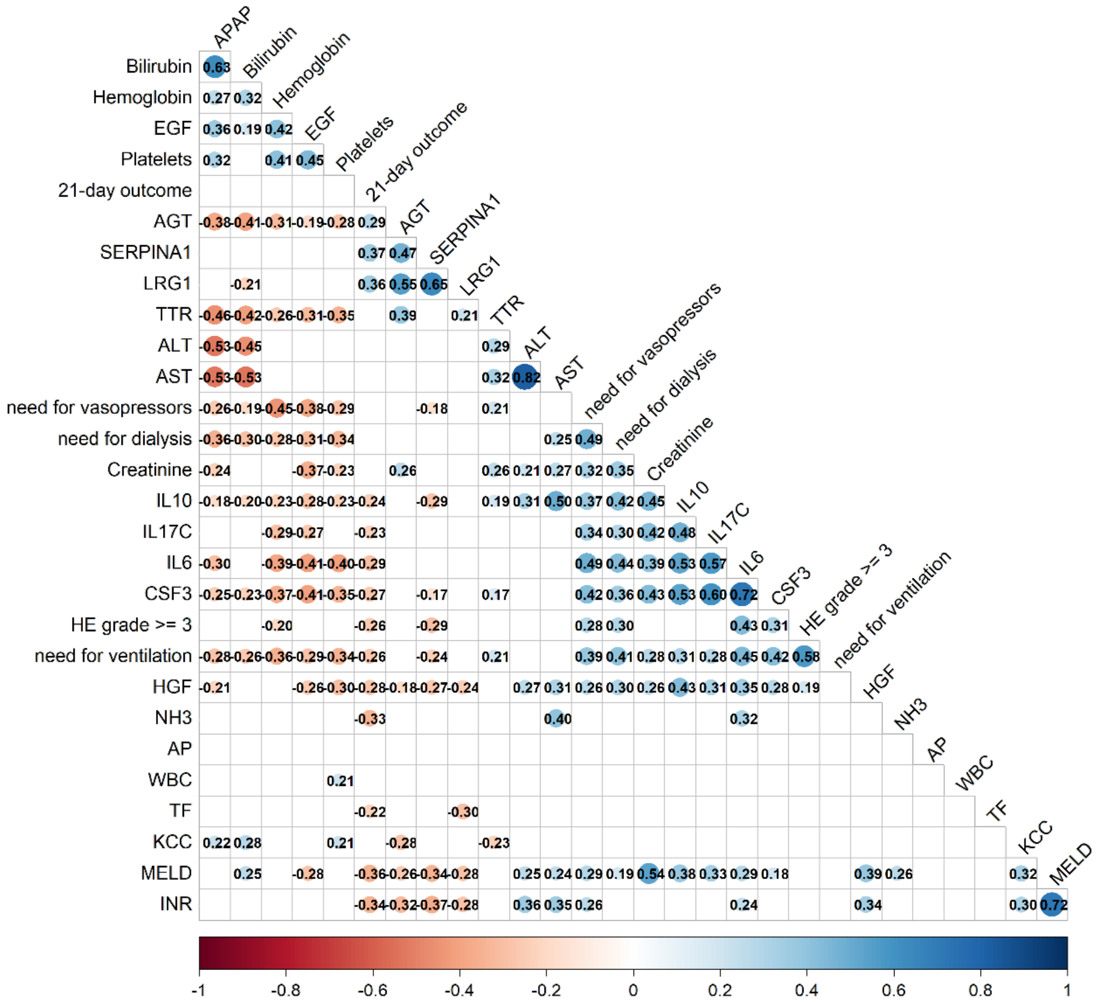

Supp. Fig. 2. Correlation between selected parameters in the discovery cohort. Spearman rank coefficients that remained statistically significant after correction for multiple testing via false-discovery rate adjustment (FDR < 0.05) are displayed. *EGF: epidermal growth factor; 21-day outcome: spontaneous survival vs liver transplantation or death during first 21 days post study admission; AGT: angiotensinogen; SERPINA1: alpha1-antitrypsin; LRG1: leucine-rich alpha-2-glycoprotein; TTR: transthyretin; ALT: alanine aminotransferase; AST: aspartate aminotransferase; IL10: interleukin 10; IL17C: interleukin 17C, IL6: interleukin 6; CSF3: colony-stimulating factor 3; HE grade >= 3: degree of hepatic encephalopathy >= 3; HGF: hepatocyte growth factor; NH3: venous ammonia levels; AP: alkaline phosphatase; WBC: white blood cell count; TF: transferrin; KCC: fulfilling King's College Criteria yes/no; MELD: model for end-stage liver disease; INR: international normalized ratio.*

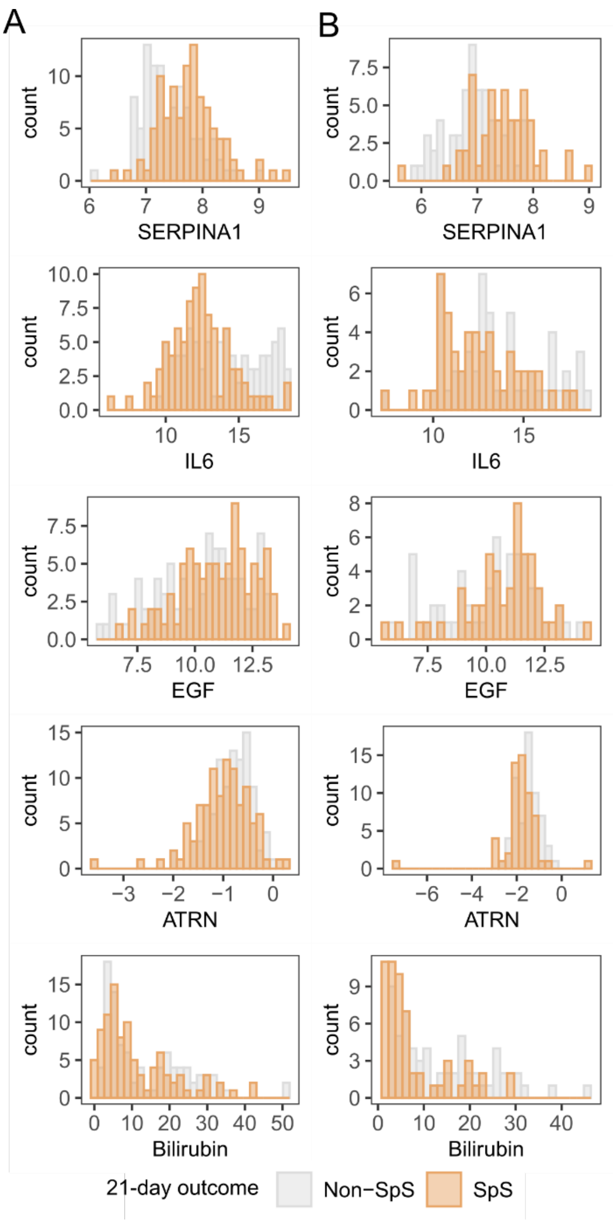

Supp. Fig. 3. Distribution of continuous parameters assessed in multivariable logistic regression. Histograms displaying the distribution of continuous parameters included in models 1 and 2 in the discovery [A] and validation [B] cohort. *SERPINA1: alpha1 antitrypsin; IL6: interleukin 6; EGF: epidermal growth factor; ATRN: attractin.*

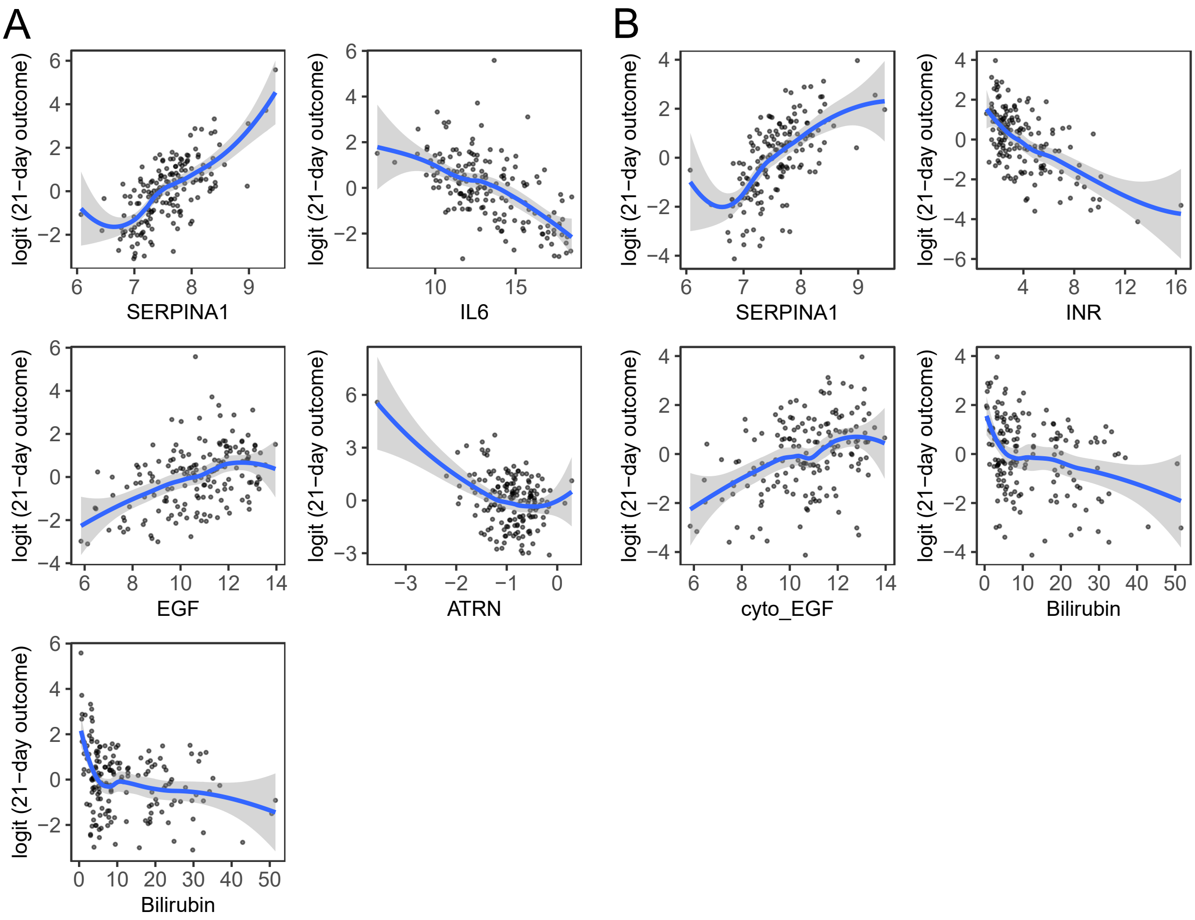

Supp. Fig. 4. Assessment of relationship between continuous modeling parameters and logit of the 21-day outcome. Plots visualizing the relationship between the continuous parameters chosen for models 1 and 2 and the logit of the 21-day outcome [A: model 1, B: model 2]. *SERPINA1: alpha1 antitrypsin; IL6: interleukin 6; EGF: epidermal growth factor; ATRN: attractin; INR: international normalized ratio.*

Supplementary References

1 **Wang Z, Karkossa I**, Großkopf H, *et al.* Comparison of quantitation methods in proteomics to define relevant toxicological information on AhR activation of HepG2 cells by BaP. *Toxicology*. 2021;448. doi: 10.1016/j.tox.2020.152652

2 **Schmidt JR, Geurtzen K**, von Bergen M, *et al.* Glucocorticoid Treatment Leads to Aberrant Ion and Macromolecular Transport in Regenerating Zebrafish Fins. *Front Endocrinol (Lausanne)*. 2019;10:1–17.

3 **Guilliams M, Bonnardel J**, Haest B, *et al.* Spatial proteogenomics reveals distinct and evolutionarily conserved hepatic macrophage niches. *Cell*. 2022;185:379-396.e38.

4 **Guilliams M, Bonnardel J**, Haest B, *et al.* [dataset] Spatial proteogenomics reveals distinct and evolutionarily-conserved hepatic macrophage niches (single cells). *Gene Expr Omnibus*. 2022;GSE192740.

Author names in bold designate shared co-first authorship.
