# Supplementary material for: Serum proteomics of adults with acute liver failure provides mechanistic insights and attractive prognostic biomarkers
Source: JHEP Rep. 2025 Jan 30;7(5):101338. doi: 10.1016/j.jhepr.2025.101338 (PMC11998117; doi:10.1016/j.jhepr.2025.101338)
Supplement: Multimedia component 2 [file mmc2.docx]

**JHEP Reports**

**CTAT methods**

Tables for a “Complete, Transparent, Accurate and Timely account” (CTAT) are now mandatory for all revised submissions. The aim is to enhance the reproducibility of methods.

- Only include the parts relevant to your study
- Refer to the CTAT in the main text as ‘Supplementary CTAT Table’
- Do not add subheadings
- Add as many rows as needed to include all information
- Only include one item per row

**If the CTAT form is not relevant to your study, please outline the reasons why:**

| Not applicable |
| --- |

- 1. **Antibodies (not applicable)**
  2. **Cell lines (not applicable)**
  3. **Organisms (not applicable)**
  4. **Sequence based reagents (not applicable)**
  5. **Biological samples**

| **Description** | **Source** | **Identifier** |
| --- | --- | --- |
| **Patient sera (patients with acute liver failure)** | Acute Liver Failure Study Group (ALFSG) registry | Not applicable |
| **Patient sera (liver-healthy individuals)** | University Hospital RWTH Aachen, collected as part of Alph1 liver initiative | Not applicable |

- 1. **Deposited data**

| **Name of repository** | **Identifier** | **Link** |
| --- | --- | --- |
| **Livercellatlas.org** | Human liver single cell RNAseq | <https://livercellatlas.org> |

- 1. **Software**

| **Software name** | **Manufacturer** | **Version** |
| --- | --- | --- |
| **R** | R Foundation, Vienna | 4.3.1 |
| **RStudio** | Posit PBC | 2023.06.1+524 |
| **imputeLCMD** |  | 2.1 |
| **SummarizedExperiment** |  | 1.34.0 |
| **limma** |  | 3.62.1 |
| **ComplexHeatmap** |  | 2.22.0 |
| **circlize** |  | 0.4.16 |
| **glmnet** |  | 4.1-8 |
| **pROC** |  | 1.18.5 |
| **corrplot** |  | 0.95 |
| **MaxQuant** | Max Planck Institute of Biochemistry, Cox group | 1.6.2.10 |
| **Ingenuity Pathway Analysis** | Qiagen | 2023 version |
| **Olink NPX Signature** | Olink | 1.7.0.0 |

- 1. **Other (*e.g*. drugs, proteins, vectors etc.)**

| **Urea** | Sigma-Aldrich | Taufkirchen, Germany |
| --- | --- | --- |
| **Dithiothreitol** | Sigma-Aldrich | Taufkirchen, Germany |
| **Trypsin** | Promega | Walldorf, Germany |
| **Dimethyl sulfoxide** | Sigma-Aldrich | Taufkirchen, Germany |
| **Formic acid** | Sigma-Aldrich | Taufkichen, Germany |
| **Acetonitrile** | Merck | Darmstadt, Germany |
| **Ammonium formate** | Sigma-Aldrich | Taufkirchen, Germany |
|  |  |  |

- 1. **Please provide the details of the corresponding methods author for the manuscript:**

| Professor Pavel Strnad  Medical Clinic III, Gastroenterology, Metabolic Diseases and Intensive Care, University Hospital RWTH Aachen, Aachen, Germany  Pauwelsstraße 30  52074 Aachen  [pstrnad@ukaachen.de](mailto:pstrnad@ukaachen.de) |
| --- |

**2.0 Please confirm for randomised controlled trials all versions of the clinical protocol are included in the submission. These will be published online as supplementary information.**

| **Not applicable** |
| --- |
