# Supplementary material for: Serum proteomics of adults with acute liver failure provides mechanistic insights and attractive prognostic biomarkers
Source: JHEP Rep. 2025 Jan 30;7(5):101338. doi: 10.1016/j.jhepr.2025.101338 (PMC11998117; doi:10.1016/j.jhepr.2025.101338)
Supplement: Multimedia component 5 [file mmc5.pdf]

Serum proteomics of adults with acute liver failure provides mechanistic insights and attractive prognostic biomarkers

Katharina Remih[1], Franziska-Maria Hufnagel[1], Anna Sophie Karl[1], Valerie Durkalski-Mauldin[2], William Martens Lee[3], Constantine J. Karvellas[4], Zemin Su[2], Jody A. Rule[3], Petra Tomanová[5], Laura Krieg[6], Isabel Karkossa[6], Kristin Schubert[6], Martin von Bergen[6], Frank Tacke[7], Sonja Luckhardt[8], Nicole Ziegler[8], Aimo Kannt[8,9], Bastian Engel[10], Richard Taubert[10], Robert John Fontana[11], Pavel Strnad[1,*], the US Acute Liver Failure Study Group

JHEP Reports **2025**. vol. 7 | 1–13

Check for updates

Background & Aims: Acute liver failure (ALF) is defined as rapid onset coagulopathy and encephalopathy in patients without a prior history of liver disease. We performed untargeted and targeted serum proteomics to delineate processes occurring in adult patients with ALF and to identify potential biomarkers.

Methods: Sera of 319 adult patients with ALF (~50% acetaminophen [APAP]-related cases) were randomly selected from admission samples of the multicenter USA Acute Liver Failure Study Group consortium and subdivided into discovery/validation cohorts. They were analyzed using untargeted proteomics with mass spectroscopy and a serum cytokine profiling and compared with 30 healthy controls. The primary clinical outcome was 21-day transplant-free survival. Single-cell RNAseq data mapped biomarkers to cells of origin; functional enrichment analysis provided mechanistic insights. Novel prognostic scores were compared with the model for end-stage liver disease and ALFSG prognostic index scores.

Results: In the discovery cohort, 117 proteins differed between patients with ALF and healthy controls. There were 167 proteins associated with APAP-related ALF, with the majority being hepatocyte-derived. Three hepatocellular proteins (ALDOB, CAT, and PIGR) robustly and reproducibly discriminated APAP from non-APAP cases (AUROCs ~0.9). In the discovery cohort, 37 proteins were related to 21-day outcome. The key processes associated with survival were acute-phase response and hepatocyte nuclear factor 1α signaling. SERPINA1 and LRG1 were the best individual discriminators of 21-day transplant-free survival in both cohorts. Two models of blood-based proteomic biomarkers outperformed the model for end-stage liver disease and ALFSG prognostic index and were reproduced in the validation cohort (AUROCs 0.83-0.86) for 21-day transplant-free survival.

Conclusions: Proteomics and cytokine profiling identified new, reproducible biomarkers associated with APAP etiology and 21-day outcome. These biomarkers may improve prognostication and understanding of the etiopathogenesis of ALF but need to be independently validated.

© 2025 The Author(s). Published by Elsevier B.V. on behalf of European Association for the Study of the Liver (EASL). This is an open access article under the CC BY license (http://creativecommons.org/licenses/by/4.0/).

Introduction

Acute liver failure (ALF) is a rare condition defined by a rapid loss of liver function within 26 weeks in the absence of pre-existing liver disease.[1] In the USA, acetaminophen (APAP) toxicity is responsible for ~50% of patients with ALF, whereas viral infection, other drug-induced liver injuries, autoimmune hepatitis, and indeterminate etiologies are other established causes.[1] ALF is characterized by severe acute injury that can rapidly evolve into multi-organ failure. It is a common indication for urgent liver transplantation (LTX), but a decision for/against liver LTX is challenging because of its rapid development, scarcity of organs, and the need for life-long immunosuppression.[2]

To facilitate the clinical management of patients with ALF, several prognostic scores have been evaluated such as the model for end-stage liver disease (MELD) and the Acute Liver Failure Study Group Prognostic Index (ALFSG-PI). However, the former showed only a moderate ability to detect a potentially lethal ALF,[3] whereas the ALFSG-PI performed less well in patients with favorable etiology and higher coma grades.[4] Although there is a constant search for novel biomarkers, a better understanding of the complex changes occurring during ALF is essential for both prediction and treatment. Therefore, large-scale assessments such as the recently published miRNA panel[5] are attractive approaches to improving the understanding of ALF pathogenesis and uncovering novel prognostic biomarkers. Serum proteomic analyses are equally promising since hepatocytes are responsible for the synthesis of the majority of serum proteins.[6] In addition to changes in secreted proteins, hepatocyte injury is characterized by an increased release of

* Corresponding author. Address: Medical Clinic III, Gastroenterology, Metabolic Diseases, and Intensive Care, University Hospital Aachen, Pauwelsstr. 30, 52074 Aachen, Germany. Tel.: +49 241 80-35324.
E-mail address: pstrnad@ukaachen.de (P. Strnad).
https://doi.org/10.1016/j.jhepr.2025.101338

various intracellular proteins such as aspartate/alanine aminotransferases (AST/ALT) that serve as liver injury markers.[6] Moreover, the synthesis of liver-derived proteins is orchestrated by various liver-enriched transcription factors and adjusted to organismal needs. For example, the body responds to inflammation via the so-called acute-phase response (APR) that leads to increased production of acute-phase proteins (APPs) and diminished synthesis of anti-APP.[7]

Although several studies demonstrated that proteins indicative of hepatocellular injury such as cytokeratin 18 – M30 (M30)[8] or hepatic synthesis of serum proteins such as factor V or hepcidin[1,9] constitute attractive ALF prognostic markers, a large-scale analysis of proteomic changes occurring during ALF is currently lacking. Therefore, we performed untargeted serum proteomics of admission samples from a large, multicenter cohort of patients with ALF who had been prospectively enrolled in the ALFSG registry study. To account for the presence of inflammation and the corresponding changes in protein synthesis, a targeted cytokine profiling was carried out. The performance of assessed proteins was compared with the multivariable ALFSG-PI and MELD scores. Network analyses were undertaken to provide mechanistic insights.

Materials and methods

Patient cohort

The patients were randomly selected from a pool of 2,244 adult patients prospectively identified and recruited at 28 tertiary North American centers participating in the Acute Liver Failure Study Group (ALFSG) between 1999 and 2019. In total, 319 patients were randomly selected from the ALFSG registry and were subdivided into a discovery cohort (200 individuals) and validation cohort (119 individuals) by approximating the distribution of major ALF etiologies found in the overall registry (Table 1). All samples were drawn on Days 1 or 2 after admission to the hospital.

All individuals met the previously defined ALF criteria.[1,8,9] The selection of patients was carried out by ALFSG staff not directly involved in the project. The institutional review boards of all participating centers approved the research, and the clinical investigation was conducted according to the principles of the 1975 Declaration of Helsinki. As the patients with ALF present with an altered mental status, written informed consent was obtained from their legal next of kin. Monitoring and therapeutic interventions were according to local institutional standards of care. Demographic, clinical, laboratory, radiologic, and 21-day outcome data were recorded prospectively.

Sera of healthy controls were collected at the University Hospital Aachen (Germany) as part of the Alpha1 liver initiative as described.[10] All cohorts were matched by age and sex.

Pre-processing of serum proteomic datasets

Before processing the data, 716 proteins were identified in the discovery cohort, and 480 proteins in the validation cohort. All reverse and 'only identified by site' entries and potential contaminants were removed. The proteomic data were then $\log_2$-transformed and filtered for parameters reaching at least 70% valid values within at least one defined subgroup (healthy controls, APAP, or non-APAP cases of ALF). To further identify outliers, group-wise coefficients of variation were calculated and features with a coefficient of variation of maximum 0.3 within at least one defined subgroup were considered for downstream analysis (188 proteins in the discovery cohort and 137 proteins in the validation cohort). The remaining missing values were imputed column-wise with random draws from a distribution of the leftmost tail of the data (based on a quantile regression) [quantile regression imputation of left-censored data (QRILC) algorithm].[11] Subsequently, the values were median-centered.

Differential abundance analysis for proteins and cytokines was performed by fitting the intensities in linear models with empirical Bayes moderation, no covariates were added. The *p* values were two-tailed and false discovery rate (FDR)-adjusted. Features were considered significantly differentially abundant across a given condition if the FDR was <0.05. Results are presented as volcano plots, plotting $\log_2$-fold changes on the x-axis and the obtained -$\log_{10}$ *p* values on the y-axis.

Sample size calculation

Sample size estimation was based on prior human -omics studies.[12–14] Further, using statistical power analysis principles outlined by Cohen *et al.*,[15] we estimated that at least 34 individuals in each subgroup are required to detect small effect sizes with a 0.8 statistical power at the 0.05 significance level. To further increase statistical power and adjust for multiple testing, we decided to include 200 individuals in the discovery cohort (sizes of subgroups: 100 spontaneous survivors, 98 death/LTX, two with missing information on outcome). The 2:1 split between the discovery and validation cohort is based on published recommendations.[16]

Statistical analyses

All analyses were conducted using the R environment[17] (R Foundation, Vienna, Austria, version 4.3.1) and R Studio[18] (version 2023.06.1+524) using the following software packages: imputeLCMD,[19] SummarizedExperiment,[20] limma,[21] ComplexHeatmap,[22,23] circlize,[24] glmnet,[25,26] pROC,[27] and corrplot.[28] For the clinical data, continuous variables were displayed as median (IQR) and compared with the Wilcoxon rank-sum test, whereas categorical variables were presented as numbers (percentage), and comparison for proportion was done using the X^2 test or Fisher's exact test. Correlations between selected biomarkers were assessed in a pairwise manner using Spearman's rank correlation test. Missing values in the clinical information were not imputed and not considered for any statistical calculations.

Principal component analysis (PCA) was carried out on the pre-processed proteomic data using the prcomp function with default parameters. Aiding the visualization, 95% CI ellipses were generated assuming a multivariate t-distribution.

Dot plots were used to display serum protein levels as determined via mass spectrometry across different conditions or etiologies. They display the median with first and third quartiles, their whiskers indicate the smallest and largest non-outlier observations.

The ability of parameters to discriminate between APAP and non-APAP etiology or between 21-day transplant-free spontaneous survivors (SpS) *vs.* non-survivors (non-SpS) was assessed using the discovery cohort via univariate logistic regression models. For developing the multivariable models, all variables that differed between SpS and non-SpS were assessed, appropriate transformations were performed, and variables were analyzed for collinearity. Variables with an area

Table 1. Comparison of study cohorts and the overall ALFSG registry data.

Parameters	Discovery (n = 200)	Validation (n = 119)	Entire registry (N = 2,244)
Age, years	42 (31–53)	39 (32–51)	40 (24)
Females, n (%)	128 (64.0)	88 (74.0)	1,550 (69.1)
Body mass index	27 (24–32)	27 (23–31)	27.04 (6.62)
Diabetes, n (%)	34 (17.0)	22 (18.5)	372 (16.6)
Caucasians, n (%)	139 (69.5)	94 (79.0)	1,675 (74.6)
Ethnicity (% Hispanic or Latino)	19 (9.5)	9 (7.6)	209 (9.3)
21-day outcome			
Spontaneous survival, n (%)	100 (51.0)	57 (47.9)	1,059 (47.2)
Death, n (%)	51 (26.0)	31 (26.0)	693 (31.9)
LTX, n (%)	47 (24.0)	32 (27.0)	532 (23.8)
**Admission labs**			
ALT (IU/L)	2,024 (656–4,507)	1,865 (632–4,500)	1,898.5 (629, 4,411)
AST (IU/L)	1,783.5 (571–4,430)	1,809 (417–6,321)	1,561 (452, 4,961)
Alkaline phosphate (IU/L)	138 (104–189)	141 (107–198)	134 (99, 186.5)
Bilirubin (mg/dl)	8.16 (4–19)	7 (4–18)	7.3 (3.7, 19.2)
Creatinine (mg/dl)	1.3 (0.80–2.65)	1.40 (0.90–2.59)	1.6 (0.9, 3.0)
Hemoglobin (g/dl)	10.85 (9.58–16.60)	10.70 (9.50–12.40)	10.9 (9.5, 12.7)
INR	3 (2.20–4.98)	2.80 (2.05–4.40)	2.8 (2.0, 4.1)
MELD score	32.5 (26–38)	31 (26–37)	33.27 (26, 38)
Platelet count ($\times 10^9$/L)	136 (86–195)	127 (73–198)	126 (83, 190)
Venous ammonia (µmol/L)	110 (70–186)	92 (63–129)	94.5 (62, 144)
Leukocyte count ($\times 10^9$/L)	10 (6.5–14.2)	9.1 (6.5–13.9)	10.25 (6.7–14.8)
Clinical parameters at admission			
Admission HE grade 3/4, n (%)	93 (48.9)	58.00 (48.7)	1,030 (47.3)
Need for pressors, n (%)	46 (23.0)	19 (16.0)	464 (20.7)
Need for RRT, n (%)	50 (25.0)	24 (20.2)	505 (22.5)
Need for ventilator, n (%)	102 (51.0)	58 (48.7)	1,062 (47.4)
Etiology			
APAP, n (%)	95 (47.5)	66 (55.5)	989 (44.1)
Autoimmune, n (%)	28 (14.0)	17 (14.3)	159 (7.1)
Hepatitis B, n (%)	34 (17.0)	10 (8.4)	166 (7.4)
Indeterminate, n (%)	33 (16.5)	12 (10.1)	276 (12.3)
Other, n (%)	10 (5.0)	14 (11.8)	654 (29.1)

Data are expressed as median (25th-75th percentile) for continuous variables and n (%) for categorical variables. Admission HE grade 3/4, grade of hepatic encephalopathy at admission equal to or larger than 3.
ALT, alanine aminotransferase; APAP, acetaminophen; AST, aspartate aminotransferase; DILI, drug-induced liver injury; INR, international normalized ratio; LTX, liver transplantation; MELD, model for end-stage liver disease; pressors, vasopressors; RRT, renal replacement therapy.

under the receiver operating characteristic (AUROC) >0.55 and *p* value <0.05 in a univariable analysis were included. Variables with sample correlations greater than 0.7 or less than -0.7 were not included in the same model to mitigate collinearity and increase the stability and accuracy of estimated coefficients. Further, variable inflation factors were assessed for the selected models. Goodness of fit was assessed using the Hosmer–Lemeshow test. Performances of uni- and multivariable logistic regression models were evaluated using the AUROC, the DeLong test[29] for correlated models, or the likelihood ratio test[30] for nested models. The linearity between assessed variables and the logit of the 21-day outcome was assessed both graphically and using the Box–Tidwell test. Influential data points were assessed via Cook's distance.

Data and results were reported using the TRIPOD reporting guidelines.[31]

For further methods, see the Supplementary information.

Results

Patients with ALF display substantial alterations in serum proteome

We compared the serum proteome of 200 representative patients with ALF (discovery cohort) (Table 1) with 30 liver-healthy controls (Table S1). The PCA (Fig. 1A) revealed marked differences between the groups with 117 proteins (*i.e.* 82% of the identified proteome) being significantly altered (FDR <0.05, Fig. 1B, Table S2). Out of those, 57 were elevated and 60 were decreased in patients with ALF *vs.* healthy controls (FDR <0.05). A mapping to publicly available liver single-cell sequencing data (Fig. 1C, Table S2) revealed that most of the altered proteins were hepatocyte-derived (68%). The 60 decreased proteins were primarily secreted proteins (*e.g.* apolipoprotein C-III [APOC3] or apolipoprotein C–I [APOC1], Fig. 1C and D), likely reflecting a loss of synthetic function. In contrast, intracellular-derived proteins (*e.g.* aldolase B [ALDOB] or fumarylacetoacetase [FAH], Fig. 1C and D) were uniformly elevated in ALF, likely reflecting the massive hepatocyte injury. von Willebrand factor (vWF), an endothelial-cell-derived factor, was the protein that was most significantly increased in patients with ALF *vs.* controls (Fig. 1B and D).

Although the untargeted proteomics uncovered only a modest number of proteins related to immune cells, many were higher in patients with ALF compared with controls. A further assessment of inflammatory reaction with cytokine profiling revealed 36 elevated proteins (*i.e.* 80% of the 45 assessed markers) in patients with ALF (Fig. 1E). Among them, the hepatocyte growth factor (HGF), a potential surrogate of liver

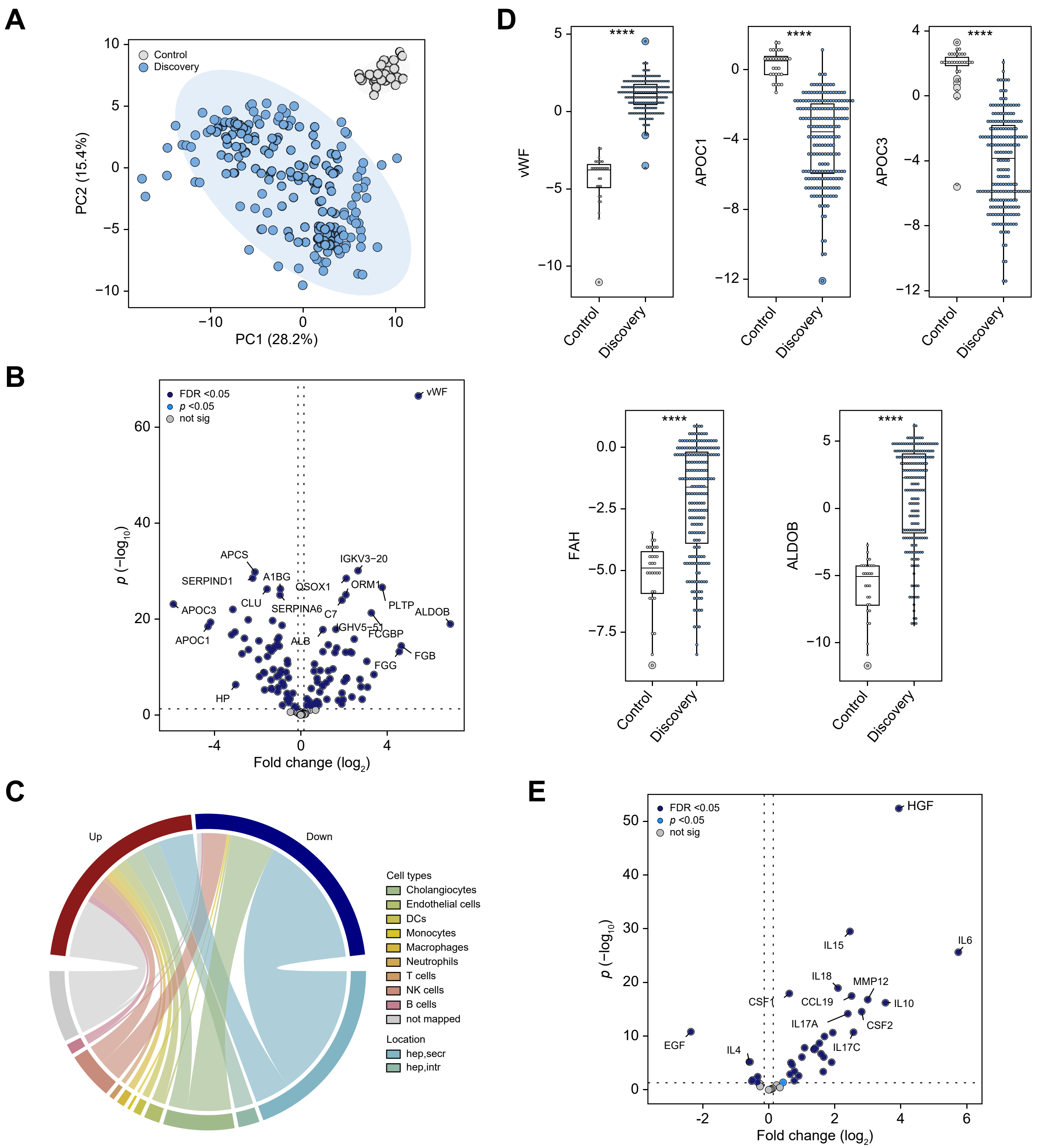

Fig. 1. Serum proteomic alterations in patients with ALF *vs.* healthy controls. (A) The first two dimensions of the principal component analysis highlight marked differences in proteomic signatures of patients with ALF (discovery cohort) and healthy controls. (B) The corresponding volcano plot depicts the 187 differentially abundant proteins (98 elevated and 89 diminished in ALF, Bayesian linear regression). A $\log_2$ fold-change >0 indicates proteins elevated in patients with ALF *vs.* healthy controls (discovery cohort). (C) Chord diagram assigning the proteins altered between patients with ALF and healthy controls to publicly available liver single-cell RNAseq data (discovery cohort). Up/down refers to features increased/decreased in patients with ALF. (D) Serum levels of characteristic proteins that differ between patients with ALF and healthy controls, displayed via dot plots. Significance levels are as follows: **p* <0.05, ***p* <0.01, ****p* <0.001 (Bayesian linear regression). (E) Volcano plot visualizing the results of the differential abundance analysis of a cytokine profiling comparing serum levels between patients with ALF and healthy controls (discovery cohort). The dotted horizontal lines in (B) and (E) depict an FDR <0.05. ALDOB, aldolase B; ALF, acute liver failure; APOC1, apolipoprotein C–I; APOC3, apolipoprotein C-III; FAH, fumarylacetoacetase, FDR, false discovery rate; NA, not available; not sig, features not reaching statistical significance in differential abundance analysis (*p* <0.05); vWF, von Willebrand factor.

Table 2. Characteristics of patients with acetaminophen- and non-APAP-induced acute liver failure.

	Discovery cohort				Validation cohort			
**Variables**	**n**	**APAP (n = 95)**	**Non-APAP (n = 105)**	***p* value**	**n**	**APAP (n = 66)**	**Non-APAP (n = 53)**	***p* value**
Age, years	200	40 (31–51)	44 (31–56)	0.174	119	36 (28–42)	46 (37–57)	<0.001
Females, n (%)	200	64 (67.4)	64 (61.0)	0.345	119	55 (83.0)	33 (62.3)	0.009
Body mass index	189	27 (23–31)	28 (24–33)	0.123	119	25 (22–30)	29 (25–31)	0.037
Diabetes, n (%)	200	13 (13.7)	21 (20)	0.235	119	10 (15.2)	12 (22.6)	0.296
Caucasians, n (%)	200	74 (77.9)	65 (61.9)	<0.001	119	59 (89.3)	35 (66.0)	0.002
Ethnicity (% Hispanic or Latino)	200	9 (9.5)	10 (9.5)	0.990	119	2 (3.0)	7 (13.2)	0.076
**21-day outcome, n (%)**								
Spontaneous survival	198	49 (52.1)	51 (49.0)	0.664	119	37 (56.1)	20 (37.7)	0.047
Death	198	23 (24.0)	28 (27.0)	0.693	119	15 (23.0)	16 (30.0)	0.357
LTX	198	22 (23.0)	25 (24.0)	0.917	119	15 (23.0)	17 (32.0)	0.253
**Admission labs**								
ALT (IU/L)	193	3,532 (2,140–5,937)	847 (352–2,251)	<0.001	117	3,550 (2,177–6,406)	632 (311–1,458)	<0.001
AST (IU/L)	192	2,901 (1,792–7,784)	836 (326–1,914)	<0.001	118	4,717 (1,663–8,560)	522 (302–1,376)	<0.001
Alkaline phosphate (IU/L)	191	130 (101–183)	146 (110–203)	0.135	118	121 (104–160)	177 (124–226)	<0.001
Bilirubin (mg/dl)	192	5 (3–7)	18 (9–25)	<0.001	118	4 (3–6)	18 (13–25)	<0.001
Creatinine (mg/dl)	199	1.90 (1.00–3.00)	1.10 (0.70–2.03)	0.002	119	1.55 (1.00–2.90)	1.02 (0.70–2.40)	0.007
Hemoglobin (g/dl)	196	10.30 (8.45–11.88)	11.60 (9.95–13.08)	<0.001	117	10.60 (9.30–12.23)	11.50 (10.00–13.20)	0.063
INR	190	3.50 (2.40–5.30)	2.65 (2.10–4.60)	0.050	119	3.25 (2.30–4.95)	2.50 (2.00–3.40)	0.007
MELD score	184	32 (25–40)	33 (27–37)	0.807	118	32 (26–36)	30 (27–39)	0.970
Platelet count (× 10^9/L)	196	114 (65–166)	152 (107–220)	<0.001	117	32 (26–36)	30 (27–39)	0.360
Venous ammonia (μmol/L)	77	134 (77–269)	99 (61–140)	0.058	73	103 (73–139)	82 (53–124)	0.113
Leukocyte count (× 10^9/L)	196	9.8 (5.7–14.5)	10.2 (6.9–13.8)	0.74	117	9.5 (6.5–15.0)	8.9 (6.7–12.1)	0.14
**Clinical parameters at admission, n (%)**								
Admission HE grade 3/4	190	53 (56.7)	40 (41.2)	0.030	119	42 (63.6)	16 (30.2)	<0.001
Need for pressors	200	32 (33.7)	14 (13.3)	<0.001	119	13 (19.7)	6 (11.3)	0.215
Need for RRT	200	39 (41.1)	11 (10.5)	<0.001	119	14 (21.2)	10 (18.9)	0.751
Need for ventilator	200	62 (65.3)	40 (38.1)	<0.001	119	44 (66.7)	14 (26.4)	<0.001
**Etiology, n (%)**								
APAP		95 (100.0)	0 (0)			66 (100.0)	0 (0)	
Autoimmune		0 (0)	28 (26.7)			0 (0)	17 (32.1)	
Hepatitis B		0 (0)	34 (32.4)			0 (0)	10 (18.9)	
DILI		0 (0)	7 (6.7)			0 (0)	14 (26.4)	
Indeterminate		0 (0)	33 (31.4)			0 (0)	12 (22.6)	
Other		0 (0)	2 (1.9)			0 (0)	0 (0)	

Data are expressed as median (25th-75th percentile) for continuous variables and n (%) for categorical variables. The *p* values were calculated using the Wilcoxon rank-sum test for continuous variables or Fisher's exact test for categorical variables. Admission HE grade 3/4, grade of hepatic encephalopathy at admission equal to or larger than 3.

ALT, alanine aminotransferase; APAP, acetaminophen; AST, aspartate aminotransferase; DILI, drug-induced liver injury; INR, international normalized ratio; LTX, liver transplantation; MELD, model for end-stage liver disease; RRT, renal replacement therapy.

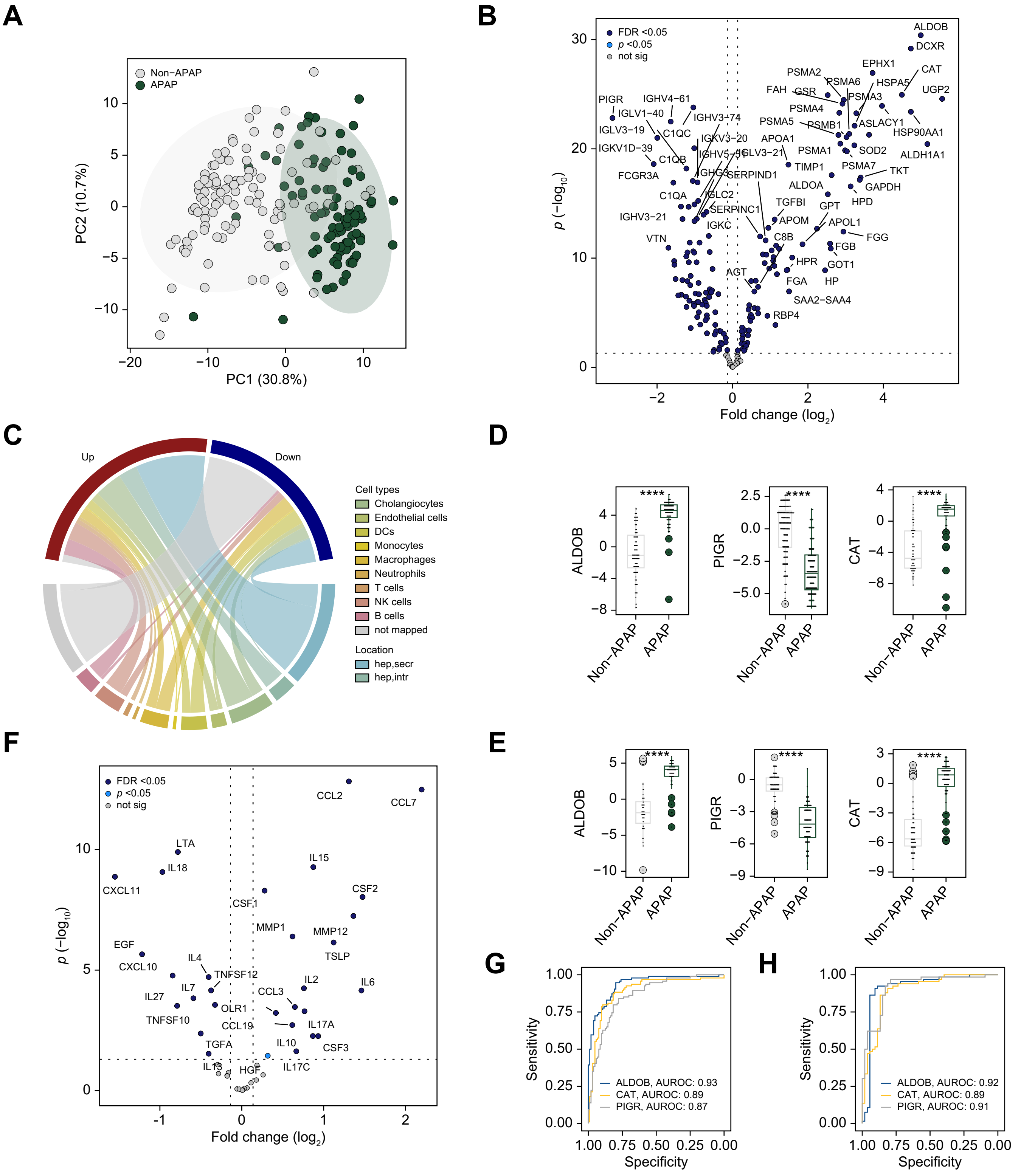

Fig. 2. Serum proteomic alterations in patients with APAP *vs.* non-APAP ALF. (A) The first two dimensions of the principal component analysis highlight profound differences in proteomic signatures of patients with APAP and non-APAP ALF (discovery cohort). (B) The corresponding volcano plot depicts 167 differentially abundant proteins (83 elevated and 84 diminished in APAP; Bayesian linear regression). A $\log_2$ fold-change >0 indicates proteins elevated in APAP *vs.* non-APAP (discovery cohort). (C) Chord diagram representing the mapping of features altered between patients with APAP and non-APAP ALF to publicly available liver single-cell RNAseq data (discovery cohort). Up/down refers to features increased/decreased in patients with APAP ALF. (D/E) Serum levels of the top three discriminating features (ALDOB, CAT, and PIGR) are depicted via dot plots for both the discovery (D) and the validation (E) cohort. Significance levels are indicated as follows: *p <0.05, **p <0.01, ***p <0.001 (Bayesian linear regression). (F) Volcano plot representing the results of the differential abundance analysis of the cytokine profiling between patients with APAP and non-APAP ALF (discovery cohort). Dotted horizontal lines in panels (B) and (F) depict FDR <0.05 (Bayesian linear regression).

Table 3. Characteristics of spontaneous survivors (SpS) and non-spontaneous survivors (non-SpS).

	Discovery cohort				Validation cohort			
**Variables**	**n**	**SpS (n = 100)**	**Non-SpS (n = 98)**	***p* value**	**n**	**SpS (n = 57)**	**Non-SpS (n = 62)**	***p* value**
Age	198	42 (30–53)	42 (32–56)	0.588	119	38 (31–52)	41 (32–49)	0.770
Sex (% female)	198	67 (68.4)	60 (60)	0.220	119	48 (84.2)	40 (64.5)	0.014
Body mass index (kg/m^2)	187	27 (24–32)	27 (24–31)	0.805	119	28 (23–32)	26 (22–30)	0.557
Diabetes, n (%)	198	18 (18.4)	16 (16.0)	0.659	119	11 (19.3)	11 (17.7)	0.827
Caucasian race, n (%)	198	67 (68.4)	70 (70.0)	0.869	119	45 (79.0)	49 (79.0)	0.950
Hispanic/Latino ethnicity, n (%)	198	10 (10.2)	9 (9.0)	0.774	119	5 (8.8)	4 (6.5)	0.736
**Admission labs**								
ALT (IU/L)	191	1,911 (690–4,074)	2,179 (637–4,507)	0.961	117	2,333 (896–5,277)	1,630 (504–3,300)	0.218
AST (IU/L)	190	1,715 (544–2,893)	1,911 (594–5,515)	0.339	118	2,043 (538–6,550)	1,524 (375–4,769)	0.546
Alkaline phosphate (IU/L)	189	135 (102–203)	139 (106–174)	0.582	118	143 (105–207)	136 (110–193)	0.951
Bilirubin (mg/dl)	190	8 (4–17)	9 (5–22)	0.064	118	5 (3–10)	11 (6–20)	<0.001
Creatinine (mg/dl)	197	1.30 (0.70–2.65)	1.40 (0.90–2.75)	0.490	119	1.16 (0.80–2.50)	1.43 (0.90–2.60)	0.375
Hemoglobin (g/dl)	194	11.15 (9.90–12.80)	10.55 (9.08–12.30)	0.088	117	10.70 (9.50–12.20)	11.25 (9.48–12.40)	0.766
INR	188	2.60 (1.90–3.73)	3.95 (2.60–5.80)	<0.001	119	2.30 (1.90–4.20)	3.20 (2.50–5.08)	0.003
MELD score	182	29 (24–35)	35 (30–41)	<0.001	118	27 (23–33)	35 (30–39)	<0.001
Platelet count (× 10^9/L)	194	142 (92–204)	126 (75–189)	0.249	117	160 (104–220)	92 (64–165)	0.001
Venous ammonia (μmol/l)	77	92 (57–124)	144 (79–264)	0.004	73	92 (69–114)	97 (63–139)	0.544
Leukocyte count (× 10^9/L)	194	9.6 (5.8–14.4)	10.7 (7.1–14.1)	0.321	117	8.8 (6.5–13.7)	9.5 (6.5–14.1)	0.717
**Clinical parameters at admission, n (%)**								
Admission HE grade 3/4	188	34.0 (35.8)	57.0 (61.3)	<0.001	119	38 (66.7)	23. (37.1)	0.001
Need for pressors	198	16 (16.0)	29 (29.6)	0.023	119	2 (3.5)	17 (27.4)	<0.001
Need for RRT	198	22 (22.0)	28 (28.6)	0.287	119	7 (12.3)	17 (27.4)	0.04
Need for ventilator	198	38 (38.0)	63 (64.3)	<0.001	119	21 (36.8)	37 (59.7)	0.013
**Etiology, n (%)**								
APAP		49 (49.0)	45 (45.9)			37 (64.9)	29 (46.8)	
Autoimmune		13 (13.0)	15 (15.3)			8 (14.0)	9 (14.5)	
Hepatitis B		17 (17.0)	16 (16.3)			0 (0)	10 (16.1)	
DILI		4 (4.0)	2 (2.0)			9 (15.8)	5 (8.1)	
Indeterminate		17 (17.0)	16.0 (16.3)			3 (5.3)	9 (14.5)	
Other		0 (0)	3 (3.0)			0 (0)	0 (0)	

Data are expressed as median (25th-75th percentile) for continuous variables and n (%) for categorical variables. The *p* values were calculated using the Wilcoxon rank-sum test for continuous variables or Fisher's exact test for categorical variables. Non-SpS and SpS refer to patients who died or required liver transplant within 21 days of enrolment and those who did not, respectively. Admission HE grade 3/4, grade of hepatic encephalopathy at admission equal to or larger than 3.
ALT, alanine aminotransferase; APAP, acetaminophen; AST, aspartate aminotransferase; Autoimmune, autoimmune hepatitis; DILI, drug-induced liver injury; INR, international normalized ratio; MELD: model for end-stage liver disease.

dysfunction,[32] and the proinflammatory interleukins IL6 and IL15 were most profoundly altered (Table S3).

Patients with APAP and non-APAP ALF have unique proteomic patterns

When comparing patients with APAP *vs.* non-APAP as the major ALF etiologies, major differences in routine parameters (Table 2) and serum proteome (Fig. 2A) were noted. Among the 167 altered proteins, 47% were derived from hepatocytes including intracellular proteins (*e.g.* ALDOB or catalase [CAT]) likely reflecting the more pronounced hepatocellular injury of patients with APAP ALF (Fig. 2B–D, Table S4). In the cytokine profiling (Fig. 2E), patients with APAP ALF displayed increased levels of CC-chemokines (*i.e.* CCL2, CCL7), whereas tumor necrosis factor family and C-X-C family cytokines were typically diminished. Hepatocellular injury markers (ALDOB, CAT) were among the top discriminators between patients with APAP and non-APAP ALF, all reaching c-statistics of ~0.9 in a univariable logistic regression model (Fig. 2F) thereby surpassing the abilities of routine parameters (c-statistics ≤0.86) and the assessed cytokines (c-statistics ≤0.81, Table S5). Notably, the major findings were reproducible in the validation cohort (Fig. 2D and F).

Admission levels of several serum proteins are associated with 21-day transplant-free survival

In the discovery cohort, 51 patients (26%) died and 47 (24%) received LTX within 21 days after admission (non-SpS), while 100 patients (50%) were SpS. Patients who were non-SpS displayed higher international normalized ratio (INR), ammonia levels, hepatic encephalopathy grades, and MELD scores (Table 3). Although no obvious separation was detected between the SpS group and non-SpS group in the PCA (Fig. 3A), 39 proteins significantly differed between the groups (FDR <0.05, Fig. 3B). The majority of these proteins were secreted by hepatocytes (27; 69%) that were nearly uniformly decreased in patients who were non-SpS (Fig. 3C, Table S6), for example alpha1-antitrypsin (SERPINA1) or leucine-rich

(G/H): Discriminative performances for the three proteins were assessed in univariable logistic regression models, results are depicted via receiver operating curves for both the discovery (G) and validation (H) cohort. ALDOB, aldolase B; ALF, acute liver failure; APAP, acetaminophen; APOC1, apolipoprotein C–I; APOC3, apolipoprotein C-III; FAH, fumarylacetoacetase; FDR, false discovery rate; NA, not available; not sig, features not reaching statistical significance in differential abundance analysis (*p* <0.05); vWF, von Willebrand factor.

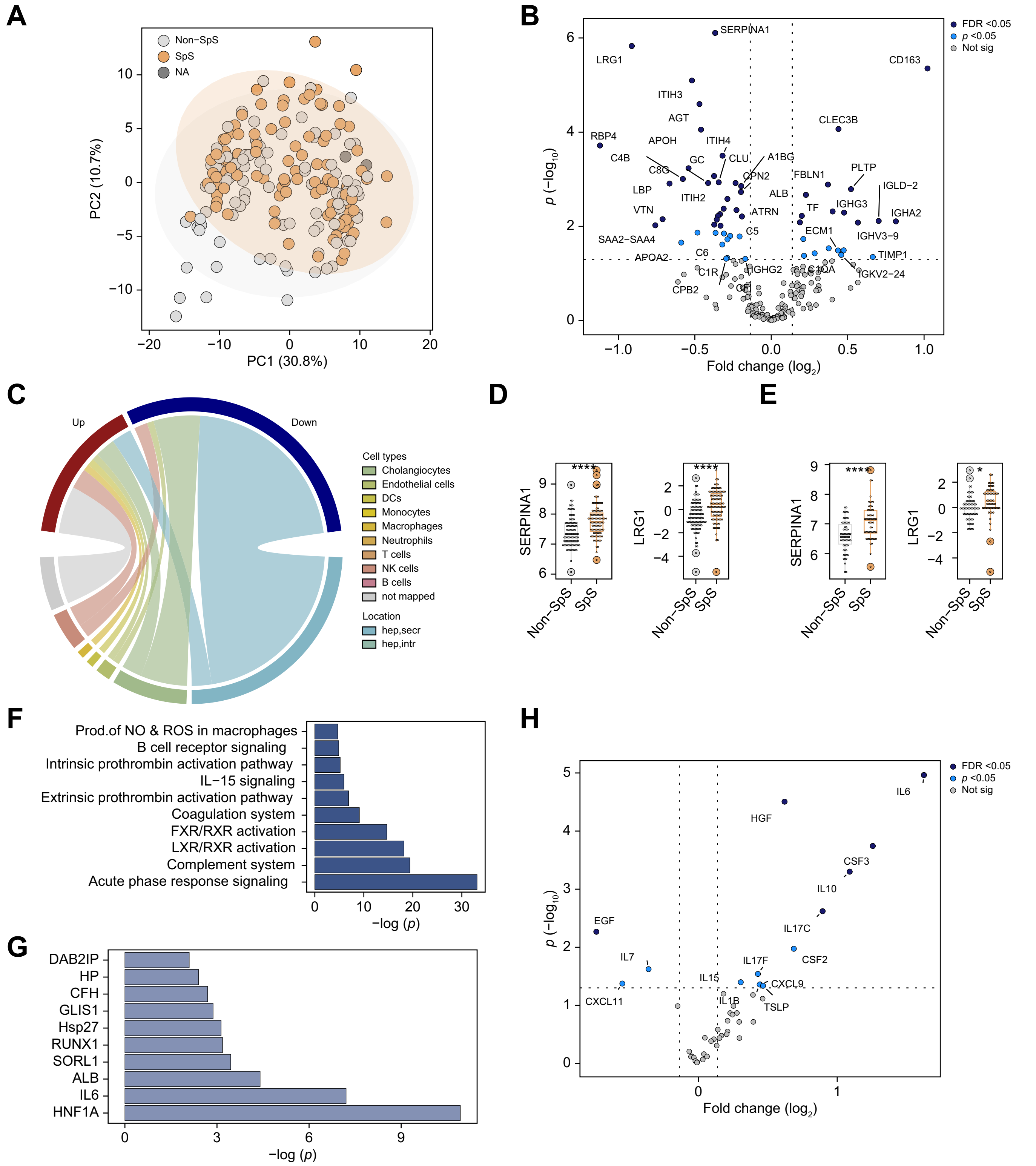

Fig. 3. Serum proteomic alterations in patients with ALF with *vs.* without spontaneous survival. (A/B) The first two dimensions of the principal component analysis (A) show no obvious separation between patients passing away or receiving liver transplantation within 21 days after admission (non-SpS) and spontaneous survivors (SpS). A 39-protein signature (12 elevated and 27 diminished in SpS) discriminated both groups as seen in the corresponding volcano plot (B) (FDR <0.05, Bayesian linear regression). A $\log_2$ fold-change >0 indicates proteins elevated in non-SpS *vs.* SpS (discovery cohort). (C) The signature of altered proteins was mapped to publicly available liver single-cell RNAseq data (discovery cohort). (D/E) SERPINA1 and LRG1 are robustly associated with 21-day outcome in both discovery (D) and validation (E) cohort. Significance levels are indicated as follows: **p* <0.05, ***p* <0.01, ****p* <0.001 (Bayesian linear regression). (F/G) Ingenuity Pathway Core Analysis (IPA) depicts 10 pathways (F) and upstream regulator networks (G) that were most significantly altered in the 21-day outcome dataset (full results, see Tables S9 and S10) (Fisher's exact test) (discovery cohort). (H) Results of the cytokine profiling comparing non-SpS and SpS are depicted in a volcano plot; six features were

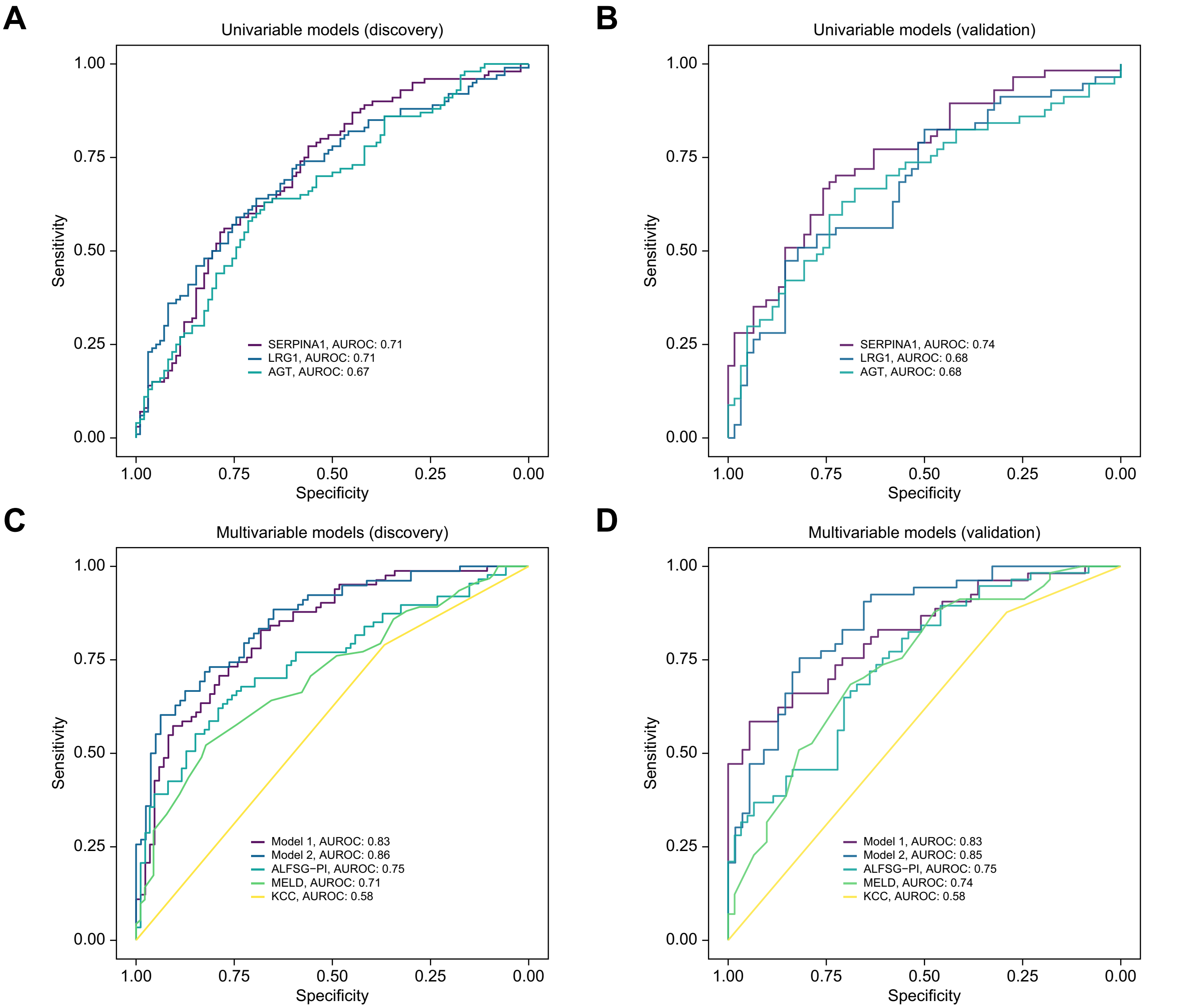

Fig. 4. Ability of selected proteins and models to predict 21-day outcome in patients with ALF. (A/B) Areas under receiver operating curves (AUROCs) delineate the ability of the top three biomarkers to predict ALF outcome in the discovery (A) and validation (B) cohort (univariable logistic regression). (C/D) AUROCs for best-performing five-feature models (model 1 comprising SERPINA1, IL6, EGF, ATRN, and serum bilirubin; model 2 comprising SERPINA1, INR, the need for mechanical ventilation, EGF and serum bilirubin) are compared to the ALFSG-PI, the model for end-stage liver disease (MELD) and the King's College Criteria (KCC) in the discovery (C) and validation (D) cohort (multivariable logistic regression). ALFSG-PI: ALFSG prognostic index; ATRN: attractin; EGF, epidermal growth factor; INR: international normalized ratio; SERPINA1: alpha1-antitrypsin.

alpha-2-glycoprotein (LRG1) (Fig. 3D). In contrast, immune-related proteins such as scavenger receptor cysteine-rich type 1 protein M130 (CD163) or various immunoglobulin components were often elevated in patients who were non-SpS (Fig. 3B and C, Table S6).

To account for the heterogeneity of our cohort regarding etiology, disease severity, and treatment modalities, the Bayes-corrected linear regression model was adjusted for different covariates (*e.g.* APAP yes/no, MELD, fulfillment of King's College Criteria (KCC), the need for dialysis or vasopressor therapy at baseline). Notably, the adjusted models yielded comparable results and the most prominent predictors remained significant across all adjusted models (Fig. S1). To obtain further insights into the processes associated with 21-day outcome, we used

differentially abundant (FDR <0.05, Bayesian linear regression) (discovery cohort). 21-day outcome, spontaneous survival *vs.* liver transplantation or death during the first 21 days post study admission; FDR, false discovery rate; hep, intr, hepatocellular intracellular features; hep, secr, hepatocellular secreted features; LRG1: leucine-rich alpha-2-glycoprotein; MELD: model for end-stage liver disease; NA, not available; not sig, features not reaching statistical significance in differential abundance analysis (p <0.05); SERPINA1: alpha1-antitrypsin.

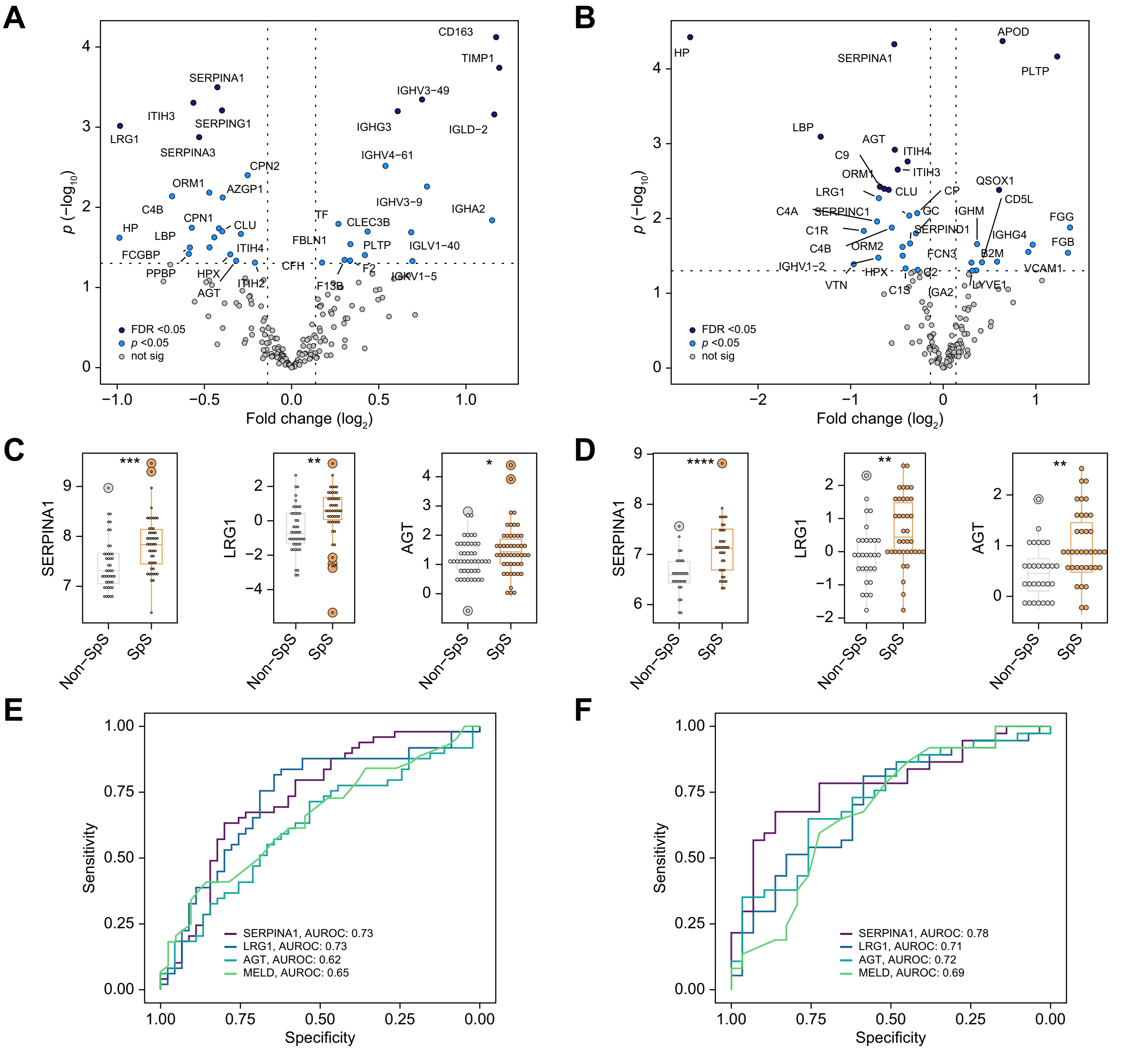

Fig. 5. Proteins associated with spontaneous survival among patients with APAP-induced ALF. (A/B) Volcano plots depicting differential abundance analysis results in patients with APAP in the discovery (A) and validation cohort (B) (Bayesian linear regression). A $\log_2$ fold-change >0 indicates proteins elevated in non-SpS *vs.* SpS. (C/D) Dot plots visualizing protein levels of features associated with 21-day outcome (SERPINA1, LRG1, AGT) in APAP cases (discovery: C, validation: D). Significance levels are indicated as follows: **p* <0.05, ***p* <0.01, ****p* <0.001 (Bayesian linear regression). (E/F) Predictive performances as determined via logistic regression (discovery: E, validation: F) of SERPINA1, LRG1, AGT, and MELD in patients with APAP. AGT, angiotensinogen; LRG1, leucine-rich alpha-2-glycoprotein; MELD, model for end-stage liver disease; not sig, features not reaching statistical significance in differential abundance analysis (*p* <0.05); SERPINA1, alpha1-antitrypsin.

Ingenuity Pathway Analysis (IPA, Qiagen, Hilden, Germany). The core analysis identified a significant (*p* <0.05) overlap with 36 pathways (Table S7), with the APR being the most significant (Fig. 3F). Hepatocyte nuclear factor 1A (HNF1A), a key liver-enriched transcription factor driving production of multiple secreted hepatocellular proteins and IL6, a master APR regulator were the top upstream regulators (Fig. 3G, Table S8). Notably, well-established IL6 targets include SERPINA1[6] and LRG1.[33] Accordingly, a targeted cytokine profiling revealed, that most proinflammatory cytokines were higher in non-SpS individuals and IL6 was the most significantly altered marker (Fig. 3H, Table S9).

SERPINA1 and LRG1 displayed a moderate correlation (R_s = 0.65, *p* <0.0001) whereas neither one markedly correlated with other established hepatic secretory proteins such as transthyretin or transferrin or their upstream regulators (*e.g.* IL6) (Fig. S2). LRG1, SERPINA1, and angiotensinogen (AGT) constituted the best discriminators for 21-day transplant-free

survival with c-statistics ~0.7 in a univariable logistic regression analysis that was similar in performance to the composite MELD score and was confirmed in the validation cohort (Fig. 4A–D, Table S10). In contrast, inflammation-related parameters did not display comparable discriminative properties with c-statistics ≤0.67 in the discovery cohort and an even worse performance in the validation cohort (Table S10).

As APAP is the leading etiology in both cohorts and clearly differs from patients with non-APAP ALF we investigated the behavior of the detected proteins in this more homogeneous subgroup. Although the volcano plots visualizing proteins that are significantly altered in patients who were SpS *vs.* non-SpS differed somewhat between the discovery and validation cohorts (Fig. 5A and B), SERPINA1 and LRG1 (Fig. 5C and D) emerged as the most robust prognostic markers reaching c-statistics 0.73/0.78 and 0.73/0.71 in the discovery and validation cohorts, respectively (Fig. 5E and F).

To predict the ALF course, we first assessed whether the addition of a proteomics-based parameter can improve the prognostic value of ALFSG-PI, however, only minor improvements were achieved (*e.g.* AUROC 0.77/0.79, p <0.0001 [likelihood ratio test] for SERPINA1 and 0.78/0.77, p <0.0001 [likelihood ratio test] for AGT in the discovery and validation cohort, respectively) (Table S11).

Next, we conducted a multivariable analysis that first considered all variables significantly associated with 21-day outcome in univariable analysis and displayed an AUROC >0.55 to be included in a potential model. We used the established ALFSG-PI score as a comparator and focused on the identification of alternative five-feature combinations. For model 1, we limited the search to continuous parameters. Among them, a combination of two secreted proteins (SERPINA1, attractin [ATRN]), two cytokines (IL6, epidermal growth factor [EGF]), and bilirubin displayed a reproducible performance (AUROCs 0.83 in both cohorts) numerically surpassing the AUROC of the ALFSG-PI score (AUROCs 0.75 in both cohorts), but the difference did not reach statistical significance. Consideration of categorical parameters yielded model 2 (encompassing SERPINA1, EGF, the INR, bilirubin, and the need for ventilation), which significantly improved the prediction (AUROC 0.86 in the discovery cohort and 0.85 in the validation cohort; p = 0.008, DeLong's test; Fig. 4C and D; Table S12, Figs S3 and S4). Both models also outperformed the MELD score (AUROC 0.71 in the discovery cohort and 0.74 in the validation cohort) and the KCC (AUROCs 0.58 in both cohorts) (Fig. 4C and D).

Discussion

In our study, we used two complementary proteomic techniques to analyze admission samples from a large multicenter observational cohort with a centralized study protocol.[1,8,9] By mapping the evaluated proteins to the corresponding liver cell populations, we demonstrated an increase in several endothelial markers and a decrease in many secreted proteins. Among the former, vWF was the protein most significantly upregulated in patients with ALF *vs.* controls. This is in line with previous results, both in ALF[34] and chronic liver disease,[35] as well as the fact that its secretion is promoted by inflammation and endothelial damage.[34,35] Regarding ALF etiologies, patients with APAP and non-APAP ALF displayed unique proteomic patterns and several hepatocellular injury markers constituted robust discriminators. This is not surprising as APAP constitutes the more acute ALF subtype displaying higher levels of routine hepatocellular damage markers.[1] Among them, ALDOB was the best analyte to diagnose APAP ALF, and its usefulness was confirmed in the validation cohort. Our finding is in line with a recent proteomic study that uncovered ALDOB as a robust marker of hepatocellular drug-induced liver injury.[36] Although ALT and ALDOB are both found in the cytoplasm and have a similar serum half-life,[37] the latter is produced primarily in mid-lobule hepatocytes whereas ALT is more periportal.[36] Because of that, ALDOB might be more sensitive to hepatocellular damage that occurs in the central area.[36] Notably, a reliable detection of APAP-related ALF is of obvious clinical importance as its course can be ameliorated by timely administration of N-acetyl cysteine and the etiology might be difficult to determine because of impaired mental status or lack of cooperation.[1,2]

A combination of pathway analysis and single-cell mapping revealed a decrease in HNF1A-induced secreted proteins as a major indicator of poor prognosis. HNFs are key liver-enriched transcription factors orchestrating the synthesis of secreted hepatocellular proteins. In line with that, a defective expression of HNF4α was suggested to drive hepatocellular failure in alcoholic hepatitis,[38] whereas its forced re-expression reversed experimental ALF.[38] An impaired APR was the key process associated with poor prognosis. This is unsurprising as APR is believed to protect the body from stresses and is involved in microbial defense.[6] For example, genetic inhibition of APR resulted in a significantly increased mortality in a mouse model of microbial infection.[39] Among the assessed APPs, the levels of SERPINA1 and LRG1 were particularly good markers of a poor prognosis. The fact that serum levels of both proteins are poorly correlated with levels of their upstream regulators further suggests that the inability of the hepatocytes to adjust protein production to the organismal needs is linked to dismal outcomes.

This observation is biologically plausible as both proteins have multiple beneficial functions. For example, LRG1 promotes angiogenesis[40] whereas SERPINA1 is a major protease inhibitor with anti-inflammatory and tissue-protective properties.[41] Among the APR regulators, a strong overlap between the outcome dataset and IL6 signaling was revealed. This finding is in line with the important role of IL6 in liver regeneration and the demonstrated impaired regeneration of IL6-deficient mice.[42,43]

It is also consistent with a previous report that uncovered hepcidin, that is another well-established APP, as a good predictor of ALF outcome.[9] In contrast to secreted proteins, cytokines and inflammatory proteins such as CD163 seem to be less robust indicators of prognosis but might be useful in disease subgroups or as adjuncts in prognostic scores.[44,45] In our modeling effort, we obtained AUROC values comparable to or even superior to previously published models[4,8] and in a direct comparison seemed to be even superior. In addition, model 1 relied only on continuous laboratory parameters, whereas the other score included variables such as coma grade that might be observer-dependent.

Although the mass spectrometry technique is robust and widely available, it misses less abundant proteins such as some intracellular proteins (e.g. lactate dehydrogenase or carbamoyl phosphate synthetase 1) which were previously suggested to be of prognostic relevance in ALF[46,47] but were detected only in a subset of our patients (data not shown).

Such proteins can be detected after depletion of the most abundant serum proteins,[46] however, this approach is known also to introduce artifacts and is too laborious for routine use. A further limitation of our work is that we were not able to compare patients with ALF with patients who had acute liver injury without ALF, that we did not assess longitudinal samples, and were not able to fully account for temporal biases and changing treatments. Such analyses may yield important additional insights and warrant further studies.

In conclusion, our study assessed the unique ALFSG cohort to detect alterations in levels of serum proteins and cyto-/chemokines in individuals with ALF of different etiologies. These findings provide novel etiopathogenic insights and uncover attractive diagnostic biomarkers as well as predictors of disease outcome. The fact that several of the identified biomarkers (*i.e.* SERPINA1, IL6) are readily available in the clinical routine should facilitate their translation. Given the limited cohort size, our study was not sufficiently powered to develop and validate a novel clinically relevant score, but rather aimed at obtaining insight into the pathophysiology in ALF. Our findings will need to be validated in independent cohorts with different non-APAP ALF etiologies.

Affiliations

[1]*Medical Clinic III, Gastroenterology, Metabolic Diseases and Intensive Care, University Hospital RWTH Aachen, Health Care Provider of the European Reference Network on Rare Liver Disorders (ERN RARE LIVER), Aachen, Germany;* [2]*Department of Public Health Sciences, Medical University of South Carolina, Charleston, SC, USA;* [3]*Department of Internal Medicine, Division of Digestive and Liver Diseases, UT Southwestern Medical Center, Dallas, TX, USA;* [4]*Department of Critical Care Medicine, University of Alberta, Edmonton, AB, Canada;* [5]*Department of Econometrics, Prague University of Economics and Business, Prague, Czechia;* [6]*Department of Molecular Systems Biology, Helmholtz Centre for Environmental Research, Leipzig, Germany;* [7]*Department of Hepatology and Gastroenterology, Charité-Universitätsmedizin, Campus Charité Mitte and Campus Virchow-Klinikum, Berlin, Germany;* [8]*Fraunhofer Institute for Translational Medicine and Pharmacology ITMP, Frankfurt am Main, Germany;* [9]*Goethe University, Institute of Clinical Pharmacology, Frankfurt am Main, Germany;* [10]*Department of Gastroenterology, Hepatology, Infectious Diseases and Endocrinology, Hannover Medical School, Hannover, Germany;* [11]*Department of Internal Medicine, Division of Gastroenterology, University of Michigan, Ann Arbor, MI, USA*

Abbreviations

APOC1, apolipoprotein C–I; APOC3, apolipoprotein C-III; APP, acute-phase protein; APR, acute-phase response; AST, aspartate aminotransferase; ATRN, attractin; AUROC, area under the receiver operating curve; CAT, catalase; CCL2, C–C motif chemokine ligand 2; CCL7, C–C motif chemokine ligand 7; CD160, scavenger receptor cysteine-rich type 1 protein; CSF, colony-stimulating factor; DILI, drug-induced liver injury; EGF, epidermal growth factor; FAH, fumarylacetoacetase; FDR, false discovery rate; HGF, hepatocyte growth factor; HNF1A, hepatocyte nuclear factor 1A; INR, international normalized ratio; IPA, Ingenuity Pathway Analysis; KCC, King's College Criteria; LRG1, leucine-rich alpha-2-glycoprotein; LTX, liver transplantation; MELD, model for end-stage liver disease; non-SpS, non-spontaneous survivors; PCA, principal component analysis; PIGR, polymeric immunoglobulin receptor; SERPINA1, alpha1-antitrypsin; SpS, 21-day transplant-free spontaneous survivors; vWF, von Willebrand factor.

Financial support

PS is supported by the German Research Foundation (DFG) consortium SFB 1382 (ID 403224013) "Gut-liver axis" and DFG grant STR 1095/6-1 (Heisenberg professorship).

Conflicts of interest

PS reports receiving grant support and lecture fees from Grifols and CSL Behring, grant support and advisory board fees from Arrowhead Pharmaceuticals, grant support from Vertex Pharmaceuticals, advisory board fees from Dicerna Pharmaceuticals and Ono Pharmaceuticals, and lecture fees from Alnylam Pharmaceuticals. RJF has received research support from Kezar Pharmaceuticals, Takeda Pharmaceuticals, and the NIH (ALFSG and DILIN). WML consults for Genentech, SeaGen, GSK, and Veristat and receives research support from Gilead, Alexion, Vivet, Camurus, and Lipocine, none related to the current article. BE was supported by the PRACTIS – Clinician Scientist program of Hannover Medical School, funded by the German Research Foundation (DFG, ME 3696/3). All other authors report no conflicts of interest.

Please refer to the accompanying ICMJE disclosure forms for further details.

Authors' contributions

Conceptualization (lead): RJF, PS. Conceptualization (equal): KR. Visualization (lead): KR.
Visualization (supporting): ASK. Methodology (lead): PS. Data curation (lead): KR. Data curation (supporting): JAR. Formal analysis (lead): KR. Formal analysis (supporting): F-MH, PT. Writing – original draft (lead): KR. Writing – review and editing (lead): KR. Writing – review and editing (supporting): VD-M, WML, CJK, ZS, JAR, KS, MvB, FT, NZ, PS. Resources (lead): PS. Resources (equal): LK, IK, KS, MvB, SL, NZ, AK, BE, RT, RJF. Supervision (lead): PS. Funding acquisition (lead): PS. Full access to the all the data and approved the final version of this manuscript: all authors.

Data availability statement

Data are available upon reasonable request to the corresponding author.

Supplementary data

Supplementary data to this article can be found online at https://doi.org/10.1016/j.jhepr.2025.101338.

References

Author names in bold designate shared co-first authorship

[1] Stravitz RT, Lee WM. Acute liver failure. Lancet 2019;394:869–881.
[2] European Association for the Study of the Liver. EASL Clinical Practical Guidelines on the management of acute (fulminant) liver failure. J Hepatol 2017;66:1047–1081.
[3] **McPhail MJW**, **Farne H**, Senvar N, et al. Ability of King's College Criteria and model for end-stage liver disease scores to predict mortality of patients with acute liver failure: a meta-analysis. Clin Gastroenterol Hepatol 2016;14:516–525.e5.
[4] Koch DG, Tillman H, Durkalski V, et al. Development of a model to predict transplant-free survival of patients with acute liver failure. Clin Gastroenterol Hepatol 2016;14:1199. 206.e2.
[5] **Tavabie OD**, **Karvellas CJ**, Salehi S, et al. A novel microRNA-based prognostic model outperforms standard prognostic models in patients with acetaminophen-induced acute liver failure. J Hepatol 2021;75:424–434.
[6] Kuscuoglu D, Janciauskiene S, Hamesch K, et al. Liver – master and servant of serum proteome. J Hepatol 2018;69:512–524.
[7] Tacke F, Luedde T, Trautwein C. Inflammatory pathways in liver homeostasis and liver injury. Clin Rev Allergy Immunol 2009;36:4–12.
[8] Rutherford A, King LY, Hynan LS, et al. Development of an accurate index for predicting outcomes of patients with acute liver failure. Gastroenterology 2012;143:1237–1243.
[9] Spivak I, Arora J, Meinzer C, et al. Low serum hepcidin is associated with reduced short-term survival in adults with acute liver failure. Hepatology 2019;69:2136–2149.
[10] Hamesch K, Mandorfer M, Pereira VM, et al. Liver fibrosis and metabolic alterations in adults with alpha-1-antitrypsin deficiency caused by the Pi*ZZ mutation. Gastroenterology 2019;157:705. 19.e18.
[11] Wei R, Wang J, Su M, et al. Missing value imputation approach for mass spectrometry-based metabolomics data. Sci Rep 2018;8:1–10.
[12] **Niu L**, **Geyer PE**, Wewer Albrechtsen NJ, et al. Plasma proteome profiling discovers novel proteins associated with non-alcoholic fatty liver disease. Mol Syst Biol 2019;15:1–16.
[13] Gurbuz B, Guldiken N, Reuken P, et al. Biomarkers of hepatocellular synthesis in patients with decompensated cirrhosis. Hepatol Int 2023;17:698–708.

[14] **Åkesson J**, **Hojjati S**, Hellberg S, et al. Proteomics reveal biomarkers for diagnosis, disease activity and long-term disability outcomes in multiple sclerosis. Nat Commun 2023;14:6903.

[15] Cohen J. Statistical power analysis for the behavioral sciences. 2nd ed. Routledge; 1988.

[16] Skates SJ, Gillette MA, LaBaer J, et al. Statistical design for biospecimen cohort size in proteomics-based biomarker discovery and verification studies. J Proteome Res 2013;12:5383–5394.

[17] Team RC. R: a language and environment for statistical computing, 2021. https://www.r-project.org/; 2021.

[18] RStudio Team. RStudio: integrated development environment for R, 2023. https://posit.co; 2023.

[19] Lazar C, Burger T. imputeLCMD: a collection of methods for left-censored missing data imputation, 2022. https://cran.r-project.org/package=imputeLCMD; 2022.

[20] Morgan M, Obenchain V, Hester J, et al. SummarizedExperiment: SummarizedExperiment container. 2021 [ductor.org/packages/Summarized Experiment].

[21] Ritchie ME, Phipson B, Wu D, et al. Limma powers differential expression analyses for RNA-sequencing and microarray studies. Nucleic Acids Res 2015;43:e47.

[22] Gu Z, Eils R, Schlesner M. Complex heatmaps reveal patterns and correlations in multidimensional genomic data. Bioinformatics 2016;32:2847–2849.

[23] Gu Z. Complex heatmap visualization. iMeta 2022;1:1–15.

[24] Gu Z, Gu L, Eils R, et al. Circlize implements and enhances circular visualization in R. Bioinformatics 2014;30:2811–2812.

[25] Friedman J, Hastie T, Tibshirani R. Regularization paths for generalized linear models via coordinate descent. J Stat Softw 2010;33:1–22.

[26] Tay JK, Narasimhan B, Hastie T. Elastic net regularization paths for all generalized linear models. J Stat Softw 2023;106.

[27] Robin X, Turck N, Hainard A, et al. pROC: an open-source package for R and S+ to analyze and compare ROC curves. BMC Bioinformatics 2011;8:12–77.

[28] Wei T, Simko V. R package 'corrplot': Visualization of a correlation matrix(Version 0.95) 2024. https://github.com/taiyun/corrplot.

[29] DeLong ER, DeLong DM, Clarke-Pearson DL. Comparing the areas under two or more correlated receiver operating characteristic curves: a nonparametric approach. Biometrics 1988;44:837–845.

[30] Moreira MJ. A conditional likelihood ratio test for structural models. Econometrica 2003;71:1027–1048.

[31] Collins GS, Reitsma JB, Altman DG, et al. Transparent reporting of a multivariable prediction model for individual prognosis or diagnosis (TRIPOD): the TRIPOD Statement. BMC Med 2015;13:1–10.

[32] Hughes RD, Zhang L, Tsubouchi H, et al. Plasma hepatocyte growth factor and biliprotein levels and outcome in fulminant hepatic failure. J Hepatol 1994;20:106–111.

[33] Shirai R, Hirano F, Ohkura N, et al. Up-regulation of the expression of leucine-rich α2-glycoprotein in hepatocytes by the mediators of acute-phase response. Biochem Biophys Res Commun 2009;382:776–779.

[34] Hugenholtz GCG, Adelmeijer J, Meijers JCM, et al. An unbalance between von Willebrand factor and ADAMTS13 in acute liver failure: implications for hemostasis and clinical outcome. Hepatology 2013;58:752–761.

[35] Ferlitsch M, Reiberger T, Hoke M, et al. Von Willebrand factor as new noninvasive predictor of portal hypertension, decompensation and mortality in patients with liver cirrhosis. Hepatology 2012;56:1439–1447.

[36] **Ravindra KC**, **Vaidya VS**, Wang Z, et al. Tandem mass tag-based quantitative proteomic profiling identifies candidate serum biomarkers of drug-induced liver injury in humans. Nat Commun 2023;14:1–14.

[37] Asaka M, Miyazaki T, Hollinger FB, et al. Human aldolase B serum levels: a marker of liver injury. Hepatology 1984;4:531–535.

[38] Argemi J, Latasa MU, Atkinson SR, et al. Defective HNF4alpha-dependent gene expression as a driver of hepatocellular failure in alcoholic hepatitis. Nat Commun 2019;10:3126.

[39] Sander LE, Sackett SD, Dierssen U, et al. Hepatic acute-phase proteins control innate immune responses during infection by promoting myeloid-derived suppressor cell function. J Exp Med 2010;207:1453–1464.

[40] Wang X, Abraham S, McKenzie JAG, et al. LRG1 promotes angiogenesis by modulating endothelial TGF-β signalling. Nature 2013;499:306–311.

[41] Strnad P, McElvaney NG, Lomas DA. Alpha1-antitrypsin deficiency. N Engl J Med 2020;38:1443–1455.

[42] Cressman DE, Greenbaum LE, DeAngelis RA, et al. Liver failure and defective hepatocyte regeneration in interleukin-6- deficient mice. Science 1996;274:1379–1383.

[43] Blindenbacher A, Wang X, Langer I, et al. Interleukin 6 is important for survival after partial hepatectomy in mice. Hepatology 2003;38:674–682.

[44] Bonkovsky HL, Barnhart HX, Foureau DM, et al. Cytokine profiles in acute liver injury—results from the US drug-induced liver injury network (DILIN) and the acute liver failure study group. PLoS One 2018;13:1–20.

[45] Glavind E, Gotthardt DN, Pfeiffenberger J, et al. The macrophage activation marker soluble CD163 is elevated and associated with liver disease phenotype in patients with Wilson's disease. Orphanet J Rare Dis 2020;15:1–9.

[46] Vazquez JH, Kennon-Mcgill S, Byrum SD, et al. Proteomics indicates lactate dehydrogenase is prognostic in acetaminophen-induced acute liver failure patients and reveals altered signaling pathways. Toxicol Sci 2022;187:25–34.

[47] **Kwan R**, **Chen L**, Park MJ, et al. The role of carbamoyl phosphate synthetase 1 as a prognostic biomarker in patients with acetaminophen-induced acute liver failure. Clin Gastroenterol Hepatol 2023;21:3060. 9.e8.

Keywords: Acute liver injury; Proteomic profiling; Acetaminophen; ALF subtyping.

Received 1 August 2024; received in revised form 17 January 2025; accepted 22 January 2025; Available online 30 January 2025

Journal of Hepatology, Volume *7*

Supplemental information

Serum proteomics of adults with acute liver failure provides mechanistic insights and attractive prognostic biomarkers

Katharina Remih, Franziska-Maria Hufnagel, Anna Sophie Karl, Valerie Durkalski-Mauldin, William Martens Lee, Constantine J. Karvellas, Zemin Su, Jody A. Rule, Petra Tomanová, Laura Krieg, Isabel Karkossa, Kristin Schubert, Martin von Bergen, Frank Tacke, Sonja Luckhardt, Nicole Ziegler, Aimo Kannt, Bastian Engel, Richard Taubert, Robert John Fontana, Pavel Strnad, and the US Acute Liver Failure Study Group

Serum proteomics of adults with acute liver failure provide mechanistic insights and attractive prognostic biomarkers

Katharina Remih, Franziska-Maria Hufnagel, Anna Sophie Karl, Valerie Durkalski-Mauldin, William Martens Lee, Constantine J. Karvellas, Zemin Su, Jody A. Rule, Petra Tomanová, Laura Krieg, Isabel Karkossa, Kristin Schubert, Martin von Bergen, Frank Tacke, Sonja Luckhardt, Nicole Ziegler, Aimo Kannt, Bastian Engel, Richard Taubert, Robert John Fontana, Pavel Strnad, and the US Acute Liver Failure Study Group

Table of contents

Supplementary Methods 2

Supplementary Tables 4

Supplementary Figures 31

Supplementary References 35

Supplementary Methods

Serum proteomics

Serum samples were diluted 1:10 in lysis buffer (8 M urea and 10 mM Dithiothreitol, both Sigma-Aldrich, Taufkirchen, Germany) and enzymatically cleaved with trypsin (Promega, Walldorf, Germany) using a paramagnetic bead approach as described previously [1]. Peptides were eluted in 2% dimethyl sulfoxide (Sigma-Aldrich, Taufkirchen, Germany), resulting in one fraction that was analysed by LC-MS/MS using an Ultimate 3000 nano ultra-performance liquid chromatography system (UPLC, Dionex, USA) coupled to a Q Exactive HF (Thermo Fisher Scientific, Waltham, USA). First, peptides were separated with a trapping column (flow rate 5ml/min, Acclaim PepMap 100 C18, 3μM, nanoViper, 75 μm x 5 cm, Thermo Scientific, Germany) and an analytical column (Acclaim PepMap 100 C18, 3 μm, nanoViper, 75 μm × 25 cm, Thermo Scientific, Germany) using a 80-minute non-linear gradient of hydrophilic solution A (0.1% formic acid (*v/v)* in ddH_2O) and hydrophobic solution B (80% acetonitrile (ACN; Merck, Darmstadt, Germany) and 0.1% ammonium formate (FA; Sigma Aldrich, Taufkirchen, Germany) in ddH_2O, *v/v*) as described previously [2]. The raw data were processed against the UniProtKB reference proteome of *Homo sapiens* (4 March 2022), using MaxQuant 1.6.2.10 default settings with label-free quantification and match between runs allowed.

Olink proteomics

Serum cytokine levels were analyzed with the proximity extension assay technology using the Olink® Target 48 Cytokine panel. Measurements were performed following the manufacturer's instructions. Quality control parameters were applied and monitored as recommended by Olink®. A total of 45 proteins were reported in relative intensities (normalized protein expression, NPX). Measurements were conducted at the Fraunhofer Institute for Translational Medicine and Pharmacology (ITMP) in Frankfurt am Main (Germany).

Downstream analysis of serum proteomic datasets

The signatures of altered features were mapped to publicly available human single-cell RNAseq data of liver tissue. Guilliams et al performed scRNAseq and CITEseq on 19 human liver biopsies. Of these, most were histologically healthy, with only 5 patients showing >10% hepatic steatosis in the absence of any significant fibrosis [3,4]. The data was accessed via www.livercellatlas.org.

Functional annotation of regulated proteins and prediction of enriched pathways and their corresponding upstream regulators were performed using Ingenuity Pathway Analysis (IPA, Qiagen). The displayed p-values reflect the overlap between the dataset and the corresponding pathways and were calculated via Fisher's exact test.

Supplementary Tables

Supp. Table 1. Characteristics of healthy controls derived from the Aachen Alpha1-registry. Data are expressed as median (IQR) for continuous variables and n (%) for categorical variables. P-values are calculated by the Wilcoxon rank sum test for continuous variables or Fisher's exact test for categorical variables. *ALT: alanine aminotransferase; AST: aspartate aminotransferase; INR: international normalized ratio.*

	Controls (n = 30)		
**variables**	**Female (n = 22)**	**Male (n = 8)**	**p**
**Age**	27.0 (33.00)	45.5 (17.0)	0.1
**Body mass index**	22.1 (3.5)	25.5 (7.1)	0.06
**admission labs**			
**ALT (IU/L)**	19.5 (10.8)	31.0 (11.8)	0.02
**AST (IU/L)**	24.0 (9.3)	25.0 (10.0)	0.67
**Alkaline phosphate (IU/L)**	63.5 (29.0)	66.0 (18.0)	0.73
**Bilirubin (mg/dL)**	0.4 (0.2)	0.6 (0.1)	0.01
**Creatinine (mg/dL)**	0.8 (0.2)	1.0 (0.2)	0.005
**INR**	0.9 (0.1)	1.0(0.1)	0.11
**Platelet count (x10^9 / l)**	240.0 (101.0)	233.5 (89.0)	0.39

Supp. Table 2. Comparison of mean protein abundancies between acute liver failure (ALF) subjects [discovery cohort] and healthy controls. A log fold change > 0 indicates proteins elevated in ALF. Data are sorted according to false discovery rate adjusted p-value (FDR). Features with an FDR < 0.05 were mapped to publicly available single-cell RNAseq data (livercellatlas.org). For hepatocellular proteins, information on their localization (i.e., secreted vs. intracellular) was added. *logFC: log2 fold change; p: p-value; FDR: false discovery adjusted p-value uniprot: uniprot identifier; gene: gene symbol.*

logFC	p	FDR	uniprot	Gene	celltypes	secreted/intracellular
5.44	2.81274E-67	3.99409E-65	P04275	VWF	Endothelial cells	
2.64	7.72508E-31	5.4848E-29	P01619	IGKV3-20		
-2.13	1.53378E-30	7.25991E-29	P02743	APCS	Hepatocytes	secreted
-2.24	2.77013E-29	8.73294E-28	P05546	SERPIND1	Hepatocytes	secreted
2.1	3.07498E-29	8.73294E-28	O00391	QSOX1		
3.76	2.26288E-27	5.35548E-26	P55058	PLTP	Endothelial cells	
-0.95	4.97842E-27	9.45924E-26	P04217	A1BG	Hepatocytes	secreted

-1.57	5.32915E-27	9.45924E-26	P10909	CLU	Hepatocytes, Cholangiocytes	secreted
2.08	8.02039E-26	1.26544E-24	P02763	ORM1	Hepatocytes	secreted
-0.97	9.06211E-26	1.28682E-24	P08185	SERPINA6	Hepatocytes	secreted
1.9	1.04017E-24	1.34277E-23	P10643	C7		
-5.91	7.16213E-24	8.47519E-23	B0YIW2	APOC3	Hepatocytes, NK cells, Cholangiocytes	secreted
-3.16	9.33522E-23	1.01969E-21	P35858	IGFALS	Hepatocytes	secreted
3.26	4.8012E-22	4.86979E-21	Q9Y6R7	FCGBP		
-2.44	1.32855E-20	1.2577E-19	P02647	APOA1	Hepatocytes, NK cells, Cholangiocytes	secreted
-1.31	2.04803E-20	1.81763E-19	P19823	ITIH2	Hepatocytes	secreted
-4.19	4.4261E-20	3.69709E-19	K7ER74	APOC4-APOC2	Hepatocytes	secreted
6.92	1.01862E-19	8.03579E-19	P05062	ALDOB	Hepatocytes, NK cells, Cholangiocytes	intracellular
-0.87	1.95021E-19	1.45753E-18	P01031	C5	Hepatocytes	secreted
-4.31	3.17783E-19	2.25626E-18	K7ERI9	APOC1	Hepatocytes, NK cells	secreted
1.62	1.28611E-18	8.69652E-18	A0A0C4DH38	IGHV5-51		
1.02	1.5397E-18	9.93809E-18	CON__P02768-1	ALB	Hepatocytes, NK cells, Cholangiocytes	secreted
-3.06	4.88613E-18	3.01665E-17	V9GYM3	APOA2	Hepatocytes, NK cells, Cholangiocytes	secreted
-3.21	1.80225E-17	1.06633E-16	P04004	VTN	Hepatocytes	secreted
-1.03	6.17464E-17	3.5072E-16	G3XAM2	CFI	Hepatocytes	secreted
-2.62	9.70833E-17	5.30224E-16	P00747	PLG	Hepatocytes, NK cells, Cholangiocytes	secreted
2.47	1.45585E-16	7.65667E-16	P0DP06	IGHV4-30-4		
-1.09	1.86387E-16	9.45247E-16	P01024	C3	Hepatocytes, NK cells, Cholangiocytes	secreted
-1.95	3.30902E-16	1.62028E-15	P0C0L5	C4B	Hepatocytes	secreted
-1.11	1.97771E-15	9.36115E-15	A0A3B3ISJ1	PROS1	Hepatocytes	secreted
1.27	2.19559E-15	1.00572E-14	P19652	ORM2	Hepatocytes	secreted
4.65	3.70658E-15	1.6448E-14	P02675	FGB	Hepatocytes, NK cells, Cholangiocytes	secreted
-1	4.20391E-15	1.80896E-14	P19827	ITIH1	Hepatocytes, Cholangiocytes	secreted
-1.21	5.08607E-15	2.12418E-14	P22792	CPN2	Hepatocytes	secreted
-1.48	9.25382E-15	3.75441E-14	P07360	C8G	Hepatocytes	secreted

1.66	9.98269E-15	3.93762E-14	Q08380	LGALS3BP	Endothelial cells	
-1.26	1.28298E-14	4.92388E-14	P02749	APOH	Hepatocytes, Cholangiocytes	secreted
-2.43	2.29371E-14	8.57124E-14	P80108	GPLD1	Hepatocytes	secreted
-1.37	4.88575E-14	1.77891E-13	P07358	C8B	Hepatocytes	secreted
4.56	5.61152E-14	1.99209E-13	P02679	FGG	Hepatocytes	secreted
1.02	5.99555E-14	2.07651E-13	P0DOY2	IGHV3-30-3		
2.29	6.87383E-14	2.32401E-13	P01714	IGLV3-19		
2.07	7.93331E-14	2.61984E-13	P12955	PEPD	Hepatocytes	intracellular
1.58	9.08226E-14	2.93109E-13	A0A4W9A917	IGHG3		
2.35	1.02545E-13	3.23587E-13	P18428	LBP	Hepatocytes	secreted
-2.73	1.64868E-13	5.08939E-13	Q5VY30	RBP4	Hepatocytes, NK cells, Cholangiocytes	secreted
-1.55	5.59395E-13	1.69009E-12	P13671	C6	Hepatocytes, Cholangiocytes	secreted
-1.97	2.57395E-12	7.61459E-12	P29622	SERPINA4	Hepatocytes	secreted
-0.63	4.12404E-12	1.19513E-11	P01042	KNG1	Hepatocytes	secreted
3.05	6.08442E-12	1.72798E-11	P16930	FAH	Hepatocytes	intracellular
-0.62	1.94825E-11	5.42453E-11	P36955	SERPINF1	B cells, Hepatocytes	secreted
0.63	2.24501E-11	6.13061E-11	P00450	CP	Hepatocytes, NK cells, Cholangiocytes	secreted
1.48	2.37859E-10	6.37282E-10	P01717	IGLV3-25		
-1.28	3.01749E-10	7.93488E-10	P02774	GC	Hepatocytes, Cholangiocytes	secreted
0.89	4.97413E-10	1.26589E-09	P01857	IGHG1		
-0.92	4.99223E-10	1.26589E-09	P01008	SERPINC1	Hepatocytes	secreted
1.27	6.11076E-10	1.52233E-09	P01876	IGHA1		
0.61	6.71624E-10	1.64432E-09	P01023	A2M	Hepatocytes, Endothelial cells	secreted
-0.69	1.16459E-09	2.80292E-09	Q14624	ITIH4	Hepatocytes	secreted
-1.72	1.39691E-09	3.30601E-09	H0YAC1	KLKB1	Hepatocytes	secreted
-1.69	1.56504E-09	3.64322E-09	Q14520	HABP2	Hepatocytes	secreted
-0.94	1.61937E-09	3.70887E-09	P25311	AZGP1	Hepatocytes, Cholangiocytes	secreted
3.38	3.43927E-09	7.75201E-09	P04424	ASL	Hepatocytes	intracellular
-1.03	5.14803E-09	1.14222E-08	P09871	C1S	Hepatocytes, Cholangiocytes	secreted
-1.92	8.90424E-09	1.94523E-08	A0A096LPE2	SAA2-SAA4	Hepatocytes	secreted
-1.12	9.31018E-09	2.0031E-08	P07357	C8A	Hepatocytes	secreted
-0.58	1.73726E-08	3.64994E-08	B4E1Z4	CFB	Hepatocytes	secreted
2.11	1.74786E-08	3.64994E-08	P04432	IGKV1D-39		
0.75	2.1984E-08	4.52424E-08	P01834	IGKC		

2.12	2.52724E-08	5.12668E-08	P02671	FGA	Hepatocytes, NK cells, Cholangiocytes	secreted
1.34	2.77143E-08	5.54287E-08	A0A0A0MS15	IGHV3-49		
2.73	3.47816E-08	6.8597E-08	P01833	PIGR		
-1.39	4.70316E-08	9.14862E-08	P02750	LRG1	Hepatocytes	secreted
2	9.30015E-08	1.78462E-07	A0A0B4J1V1	IGHV3-21		
0.77	1.31467E-07	2.48911E-07	A0A087X1J7	GPX3		
-0.87	1.45728E-07	2.72281E-07	P04196	HRG	Hepatocytes	secreted
1.17	1.65106E-07	3.04482E-07	P01591	IGJ	B cells	
-0.83	3.85093E-07	7.01067E-07	P04003	C4BPA	Hepatocytes	secreted
-3.02	4.4699E-07	8.0345E-07	P00738	HP	Hepatocytes	secreted
1	5.05502E-07	8.97267E-07	P80748	IGLV3-21		
-0.8	7.41647E-07	1.30017E-06	C9JF17	APOD		
1.01	8.74756E-07	1.51482E-06	O43866	CD5L	Macrophages, DCs	
2.78	1.24682E-06	2.13311E-06	Q03154	ACY1	Hepatocytes	secreted
3.08	1.47859E-06	2.49952E-06	P04040	CAT	Hepatocytes, Erythroblasts, Platelets	intracellular
-1.04	1.6628E-06	2.77785E-06	P06727	APOA4		
0.48	2.8457E-06	4.69676E-06	P01009	SERPINA1	Hepatocytes	secreted
-1.38	2.87759E-06	4.69676E-06	P0C0L4	C4A	Hepatocytes	secreted
-1.34	4.01996E-06	6.48675E-06	P27169	PON1	Hepatocytes	secreted
-1.68	5.59654E-06	8.92931E-06	O14791	APOL1	Hepatocytes	secreted
-1.08	1.37298E-05	2.16626E-05	P02790	HPX	Hepatocytes, NK cells, Cholangiocytes	secreted
0.87	1.53258E-05	2.3915E-05	P02745	C1QA	Macrophages, DCs	
1.9	2.50458E-05	3.86577E-05	P24298	GPT	Hepatocytes	intracellular
-0.38	2.77541E-05	4.23773E-05	A0A7I2V2D2	SERPING1	Hepatocytes, Endothelial cells	secreted
-0.64	3.06918E-05	4.63643E-05	P00734	F2	Hepatocytes	secreted
2.36	0.00016456	0.000245974	Q7Z4W1	DCXR	Hepatocytes	intracellular
-0.55	0.000215421	0.000318643	A0A7P0T8D1	AGT	Hepatocytes, Cholangiocytes	secreted
0.27	0.000389032	0.00056951	P08603	CFH	Hepatocytes, NK cells, Cholangiocytes	secreted
-0.64	0.000394123	0.000571076	P02748	C9	Hepatocytes	secreted
-0.51	0.000408736	0.000586268	P04114	APOB	Hepatocytes, NK cells, Cholangiocytes	secreted
1.2	0.000507429	0.000713973	P01861	IGHG4		
2.84	0.000507826	0.000713973	P00352	ALDH1A1	Hepatocytes	intracellular
1.95	0.000604835	0.000842025	P32754	HPD	Hepatocytes, NK cells, Cholangiocytes	secreted

1.73	0.000847021	0.001167738	P29401	TKT	Macrophages, DCs	
-0.61	0.000880793	0.001202621	A0A3B3ISR2	C1R	Hepatocytes, Cholangiocytes	secreted
0.37	0.001136604	0.001537121	P51884	LUM		
0.73	0.00157569	0.00211083	P23142	FBLN1		
0.79	0.001848221	0.00245278	P63261	ACTG1	T cells, B cells	
-0.56	0.00247251	0.003250892	P02751	FN1	Hepatocytes, Cholangiocytes	secreted
0.58	0.003923617	0.005111501	P01871	IGHM		
1.89	0.004889676	0.006312127	P07900	HSP90AA1	T cells, B cells	
0.63	0.006067625	0.007762187	P04433	IGKV3D-11		
0.82	0.006259799	0.007936531	P01700	IGLV1-47		
-0.86	0.008284096	0.010410103	P07996	THBS1	Monocytes	
0.27	0.009787909	0.012191956	P02787	TF	Hepatocytes, NK cells, Cholangiocytes	secreted
0.45	0.012968705	0.016013532	O75636	FCN3	Hepatocytes, Endothelial cells	secreted
-0.31	0.014686437	0.017978224	P05543	SERPINA7	Hepatocytes	secreted
-0.2	0.03329387	0.040407945	P08697	SERPINF2	Hepatocytes, NK cells, Cholangiocytes	secreted
0.47	0.05276064	0.063491618	P06312	IGKV4-1		
0.42	0.085908649	0.102512841	P04211	IGLV7-43		
0.68	0.091692424	0.108502702	P04075	ALDOA		
-0.32	0.105516716	0.123829535	A0A087WT59	TTR		
0.27	0.133521433	0.155410192	A0A0A0MSV6	C1QB		
-0.29	0.162600301	0.187717421	P00748	F12		
0.19	0.19382011	0.221955287	P01011	SERPINA3		
-0.49	0.238088266	0.27046827	P01594	IGKV1-33		
-0.14	0.273265677	0.307966081	A0A4W8ZXM2	IGHV3-72		
-0.18	0.28042084	0.313541411	Q96PD5	PGLYRP2		
0.14	0.364173859	0.404005374	P02747	C1QC		
-0.15	0.368575218	0.405718457	A0A075B6S2	IGKV2D-29		
-0.08	0.464151906	0.504363894	P02765	AHSG		
0.1	0.465293452	0.504363894	A0A0B4J231	IGLL5		
0.21	0.540759058	0.581725654	A0A0B4J1U7	IGHV6-1		
0.15	0.567844233	0.603171365	P01624	IGKV3-15		
0.2	0.56918988	0.603171365	P00739	HPR		
0.08	0.580748575	0.607720984	P43652	AFM		
0.13	0.582042633	0.607720984	Q16610	ECM1		
0.15	0.750839596	0.778242501	P01602	IGKV1-5		
0.05	0.782133871	0.804804418	Q06033	ITIH3		
0.03	0.824597581	0.842394651	P02760	AMBP		

-0.04	0.839370993	0.851362008	P02649	APOE		
-0.02	0.867995359	0.874151354	P01859	IGHG2		
-0.01	0.91317593	0.91317593	O75882	ATRN		

Supp. Table 3. Comparison of mean cytokine levels between acute liver failure cases (ALF) [discovery cohort] and healthy controls. Data are sorted according to false discovery rate adjusted p-value (FDR). *logFC: fold change of the log-transformed cytokine abundancies.*

LogFC	p	FDR	uniprot ID	gene symbol
3.9400	4.12E-53	1.86E-51	P14210	HGF
2.4624	3.27E-30	7.35E-29	P40933	IL15
5.7436	2.42E-26	3.62E-25	P05231	IL6
2.0981	1.15E-19	1.29E-18	Q14116	IL18
0.6236	1.17E-18	1.06E-17	P09603	CSF1
2.5108	3.35E-18	2.51E-17	Q99731	CCL19
2.9987	1.64E-17	1.05E-16	P39900	MMP12
3.5357	6.21E-17	3.49E-16	P22301	IL10
2.8158	2.83E-15	1.41E-14	P04141	CSF2
2.3964	6.76E-15	3.04E-14	Q16552	IL17A
-2.3584	1.54E-11	6.32E-11	P01133	EGF
2.5691	1.93E-11	7.25E-11	Q9P0M4	IL17C
1.9404	2.33E-11	8.07E-11	Q07325	CXCL9
1.6882	1.18E-10	3.78E-10	P02778	CXCL10
1.5349	2.13E-09	6.39E-09	Q96PD4	IL17F
1.0868	1.49E-08	4.19E-08	P01375	TNF
1.4105	1.73E-08	4.58E-08	P13500	CCL2
1.3730	2.98E-08	7.45E-08	P10147	CCL3
1.3872	3.24E-08	7.66E-08	P60568	IL2
1.5857	1.96E-07	4.42E-07	Q969D9	TSLP
1.6667	8.13E-07	1.68E-06	P10145	CXCL8
1.0048	8.21E-07	1.68E-06	O95760	IL33
-0.5841	6.43E-06	1.21E-05	P05112	IL4
-0.5668	6.47E-06	1.21E-05	O43508	TNFSF12
1.9054	7.49E-06	1.35E-05	P09919	CSF3
0.6738	9.12E-06	1.58E-05	P51671	CCL11
0.7084	2.24E-05	3.73E-05	P03956	MMP1
0.7805	3.66E-04	5.88E-04	P48061	CXCL12
1.6512	4.29E-04	6.65E-04	P80098	CCL7
0.6484	1.28E-03	1.92E-03	P13236	CCL4
0.9066	2.53E-03	3.68E-03	Q8NEV9_Q14213	IL27
-0.3344	3.73E-03	5.25E-03	P50591	TNFSF10
-0.4887	1.48E-02	2.02E-02	P13232	IL7
0.7752	2.05E-02	2.71E-02	O14625	CXCL11
-0.5136	2.42E-02	3.12E-02	P13725	OSM

-0.3324	3.94E-02	4.93E-02	P01374	LTA
0.4348	4.30E-02	5.24E-02	P80075	CCL8
-0.2802	7.00E-02	8.29E-02	P49771	FLT3LG
0.2385	1.37E-01	1.59E-01	P78380	OLR1
-0.2600	2.39E-01	2.69E-01	P01135	TGFA
0.3380	3.71E-01	4.07E-01	P01579	IFNG
0.1157	5.63E-01	6.03E-01	Q99616	CCL13
0.0739	7.71E-01	8.07E-01	P35225	IL13
0.0398	8.90E-01	9.10E-01	P01584	IL1B
0.0004	9.98E-01	9.98E-01	P15692	VEGFA

Supp. Table 4. Comparison of mean protein abundancies between acetaminophen (APAP)- and non-APAP-induced acute liver failure cases [discovery cohort]. A log fold change > 0 indicates proteins elevated in APAP subjects. Data are sorted according to false discovery rate adjusted p-value (FDR). Features with an FDR < 0.05 were mapped to publicly available single cell RNAseq data (livercellatlas.org). For hepatocellular proteins, information on their behavior (i.e., secreted vs. intracellular) was added. *logFC: log fold change; p: p-value; FDR: false discovery adjusted p-value; uniprot: uniprot identifier; gene: gene symbol.*

logFC	P	FDR	uniprot	gene	celltypes	secreted/intracellular
4.99	3.97708E-31	7.47691E-29	P05062	ALDOB	Hepatocytes, NK cells, Cholangiocytes	intracellular
4.73	6.57044E-30	6.17621E-28	Q7Z4W1	DCXR	Hepatocytes	intracellular
3.71	1.12654E-27	7.05964E-26	P07099	EPHX1	Hepatocytes	intracellular
4.49	1.13721E-25	4.5237E-24	P04040	CAT	Hepatocytes, Erythroblasts, Platelets	intracellular
2.52	1.20311E-25	4.5237E-24	P00390	GSR		
5.56	2.65873E-25	8.33069E-24	Q16851	UGP2	T cells, Hepatocytes	intracellular
2.95	3.26205E-25	8.76093E-24	A0A024RA52	PSMA2		
2.92	7.38275E-25	1.73495E-23	P16930	FAH	Hepatocytes	intracellular
3.96	1.16774E-24	2.43928E-23	Q03154	ACY1	Hepatocytes	secreted
-1.04	1.61494E-24	3.03609E-23	P02747	C1QC	Macrophages, DCs	
4.73	4.07298E-24	6.9611E-23	P07900	HSP90AA1	T cells, B cells	
2.83	5.03868E-24	7.89392E-23	H0YMZ1	PSMA4	B cells	
3.28	5.55991E-24	8.04049E-23	P25788	PSMA3	Endothelial cells, B cells	
-3.18	1.5078E-23	2.02476E-22	P01833	PIGR		
-1.64	3.20525E-23	4.01725E-22	A0A0C4DH41	IGHV4-61		
3.24	7.65237E-23	8.99153E-22	P11021	HSPA5	B cells, Endothelial cells	
3.09	4.2939E-22	4.74855E-21	G3V5Z7	PSMA6		
3.62	5.18873E-22	5.33636E-21	P04424	ASL	Hepatocytes	intracellular
2.81	5.39313E-22	5.33636E-21	P28066	PSMA5	B cells	
3.02	8.58026E-22	8.06544E-21	P20618	PSMB1	Macrophages, DCs, Endothelial cells	
-1.99	9.93231E-22	8.89178E-21	P01703	IGLV1-40		
2.86	3.36758E-21	2.87775E-20	A0A7P0TAE1	HSP90B1	Macrophages, DCs, B cells	
5.16	3.63564E-21	2.97174E-20	P00352	ALDH1A1	Hepatocytes	intracellular
3.23	4.78887E-21	3.75128E-20	P04179	SOD2	Neutrophils	

-1.01	8.71818E-21	6.55607E-20	A0A0A0MSV6	C1QB	Macrophages, DCs	
2.99	1.3676E-20	9.88882E-20	P25786	PSMA1	Hepatocytes, B cells	intracellular
3.03	1.70671E-20	1.18837E-19	O14818	PSMA7	Macrophages, DCs, Endothelial cells	
-2.09	2.44909E-19	1.64439E-18	P04432	IGKV1D-39		
1.48	2.77914E-19	1.80165E-18	P02647	APOA1	Hepatocytes, NK cells, Cholangiocytes	secreted
-1.22	6.37517E-19	3.99511E-18	P01714	IGLV3-19		
2.63	2.54755E-18	1.54496E-17	Q5H9A7	TIMP1	Macrophages, DCs, Endothelial cells	
3.4	4.21454E-18	2.47605E-17	P29401	TKT	Macrophages, DCs	
3.37	6.86274E-18	3.90968E-17	P04406	GAPDH	B cells, Hepatocytes	intracellular
-1.05	8.70364E-18	4.8126E-17	A0A0B4J1X5	IGHV3-74		
-0.93	1.18836E-17	6.3832E-17	P01619	IGKV3-20		
-1.57	1.24301E-17	6.49125E-17	H0Y755	FCGR3A	Macrophages, DCs	
3.13	2.72222E-17	1.38318E-16	P32754	HPD	Hepatocytes, NK cells, Cholangiocytes	secreted
2.53	1.43129E-16	7.08114E-16	P04075	ALDOA	Monocytes	
-0.91	5.76981E-16	2.78135E-15	A0A0C4DH38	IGHV5-51		
-1.01	1.23161E-15	5.78857E-15	P02745	C1QA	Macrophages, DCs	
-1.37	1.9129E-15	8.77134E-15	P01624	IGKV3OR2-268		
-1.16	2.02407E-15	9.06013E-15	P01700	IGLV1-47		
-0.69	5.85995E-15	2.56203E-14	P0DOY2	IGLC2		
-0.77	1.07651E-14	4.59963E-14	P01857	IGHG1		
-0.95	2.5504E-14	1.0655E-13	P80748	IGLV3-21		
-1.32	2.64535E-14	1.08114E-13	A0A0B4J1V1	IGHV3-21		
1.12	2.94487E-14	1.17795E-13	Q15582	TGFBI	Macrophages, DCs, B cells	
-1.01	4.12618E-14	1.61609E-13	A0A4W9A917	IGHG3		
0.95	1.69827E-13	6.51581E-13	O95445	APOM	Hepatocytes	secreted
2.24	2.02712E-13	7.62197E-13	P24298	GPT	Hepatocytes	intracellular
2.94	3.78153E-13	1.39398E-12	P02679	FGG	Hepatocytes	secreted
-0.63	9.35607E-13	3.38258E-12	P01834	IGKC		
0.73	1.04673E-12	3.71294E-12	P01008	SERPINC1	Hepatocytes	secreted
0.88	2.38962E-12	8.31943E-12	P05546	SERPIND1	Hepatocytes	secreted
-1.13	3.83205E-12	1.2907E-11	A0A0A0MS15	IGHV3-49		
-0.87	3.84464E-12	1.2907E-11	P01717	IGLV3-25		
2.59	4.73263E-12	1.56094E-11	P02675	FGB	Hepatocytes, NK cells, Cholangiocytes	secreted

1.86	5.48507E-12	1.77792E-11	O14791	APOL1	Hepatocytes	secreted
1.16	7.25324E-12	2.3112E-11	I3L4N8	ACTG1	T cells, B cells	
-1.7	1.12756E-11	3.53302E-11	P04004	VTN	Hepatocytes	secreted
2.61	1.32726E-11	4.09058E-11	P17174	GOT1	Hepatocytes	intracellular
1.24	1.44095E-11	4.36935E-11	P27169	PON1	Hepatocytes	secreted
1.04	1.98244E-11	5.91585E-11	P12955	PEPD	Hepatocytes	intracellular
-1.04	2.398E-11	7.04413E-11	P06312	IGKV4-1		
-0.6	2.76716E-11	8.00348E-11	A0A0B4J231	IGLL5	B cells	
-1.19	3.03312E-11	8.63981E-11	P01599	IGKV1-17		
-1.01	3.14296E-11	8.81905E-11	A0A0C4DH67	IGKV1-8		
0.81	4.60121E-11	1.2721E-10	Q96PD5	PGLYRP2	Hepatocytes	secreted
0.9	4.87806E-11	1.3291E-10	A0A087WT59	TTR	Hepatocytes, NK cells, Cholangiocytes	secreted
-1.22	6.99576E-11	1.87886E-10	P01742	IGHV1-69		
1.08	8.12614E-11	2.15171E-10	H0YAC1	KLKB1	Hepatocytes	secreted
1.58	8.87559E-11	2.31751E-10	V9GYM3	APOA2	Hepatocytes, NK cells, Cholangiocytes	secreted
1.08	1.69503E-10	4.36527E-10	P29622	SERPINA4	Hepatocytes	secreted
-1.55	1.86652E-10	4.74198E-10	A0A0B4J1U7	IGHV6-1		
-1.16	2.38514E-10	5.94531E-10	Q15848	ADIPOQ		
-0.95	2.40342E-10	5.94531E-10	P01780	IGHV3-7		
0.85	2.87314E-10	7.01494E-10	P13671	C6	Hepatocytes, Cholangiocytes	secreted
-1.54	4.07776E-10	9.82845E-10	A0A0B4J1V2	IGHV2-26		
1.11	5.19482E-10	1.23624E-09	P02790	HPX	Hepatocytes, NK cells, Cholangiocytes	secreted
0.97	8.96334E-10	2.10639E-09	P55058	PLTP	Endothelial cells	
-0.53	9.84045E-10	2.28396E-09	A0A4W8ZXM2	IGHV3-72		
1.46	1.16616E-09	2.67363E-09	P00739	HPR	Hepatocytes	
2.45	1.2972E-09	2.91408E-09	P00738	HP	Hepatocytes	secreted
1.44	1.30203E-09	2.91408E-09	P02671	FGA	Hepatocytes, NK cells, Cholangiocytes	secreted
1.18	2.93864E-09	6.49958E-09	P18428	LBP	Hepatocytes	secreted
-1.11	4.29559E-09	9.39037E-09	P02775	PPBP		
-1.51	8.25175E-09	1.78314E-08	P04211	IGLV7-43		
-1.33	1.03813E-08	2.21783E-08	P01602	IGKV1-5		
0.62	1.17823E-08	2.48884E-08	P04196	HRG	Hepatocytes	secreted
0.5	1.25678E-08	2.62527E-08	P19827	ITIH1	Hepatocytes, Cholangiocytes	secreted
-1.2	1.27873E-08	2.64178E-08	P04433	IGKV3D-11		
-0.9	1.87555E-08	3.83264E-08	P01591	IGJ	B cells	
-1.12	2.98699E-08	6.03822E-08	P00747	PLG	Hepatocytes, NK cells, Cholangiocytes	secreted
0.68	4.27623E-08	8.55245E-08	P07358	C8B	Hepatocytes	secreted

-0.6	7.0474E-08	1.39464E-07	P23142	FBLN1		
-0.54	8.87896E-08	1.7388E-07	P01859	IGHG2		
1.5	1.11536E-07	2.16173E-07	A0A096LPE2	SAA2-SAA4	Hepatocytes	secreted
0.58	1.12903E-07	2.1659E-07	A0A7P0T8D1	AGT	Hepatocytes, Cholangiocytes	secreted
-0.56	1.32949E-07	2.52469E-07	P04114	APOB	Hepatocytes, NK cells, Cholangiocytes	secreted
-0.7	1.44855E-07	2.72328E-07	Q08380	LGALS3BP	Endothelial cells	
-0.98	1.72887E-07	3.21809E-07	Q9Y6R7	FCGBP		
-0.37	1.9678E-07	3.62692E-07	P05543	SERPINA7	Hepatocytes	secreted
-0.89	2.01815E-07	3.68361E-07	P0C0L5	C4B	Hepatocytes	secreted
-1.36	2.24229E-07	4.05337E-07	P02776	PF4		
-1.44	2.6349E-07	4.71773E-07	A0A0C4DH34	IGHV4-28		
-1.23	3.36227E-07	5.96326E-07	P01701	IGLV1-51		
-0.62	3.64442E-07	6.40328E-07	P02649	APOE	Macrophages, DCs, Hepatocytes	secreted
-0.74	6.7062E-07	1.16737E-06	P01876	IGHA1		
-1.14	7.9125E-07	1.36473E-06	A0A0B4J1Y8	IGLV9-49		
-0.93	1.05021E-06	1.79018E-06	P0C0L4	C4A	Hepatocytes	secreted
-0.9	1.05697E-06	1.79018E-06	A0A075B6K4	IGLV3-10		
-0.71	1.2796E-06	2.1479E-06	P01871	IGHM		
-1.03	1.48609E-06	2.47243E-06	P01782	IGHV3-9		
-0.8	1.81739E-06	2.9971E-06	P10643	C7		
0.44	2.0344E-06	3.3258E-06	P19823	ITIH2	Hepatocytes	secreted
-0.68	2.38445E-06	3.86445E-06	O43866	CD5L	Macrophages, DCs	
-1.02	2.40826E-06	3.86968E-06	A0A0C4DH68	IGKV2-24		
-1.22	2.9453E-06	4.6925E-06	A0A0J9YY99	IGLD-2		
0.58	3.70078E-06	5.84662E-06	P07360	C8G	Hepatocytes	secreted
0.67	4.07043E-06	6.377E-06	P07357	C8A	Hepatocytes	secreted
0.42	5.16907E-06	8.03128E-06	P43251	BTD		
-0.46	8.9495E-06	1.37107E-05	A0A087X1J7	GPX3		
-0.89	8.97033E-06	1.37107E-05	A0A182DWH7	SEPP1	Macrophages, DCs, Hepatocytes	
0.68	1.14606E-05	1.73758E-05	P02774	GC	Hepatocytes, Cholangiocytes	secreted
0.49	1.56852E-05	2.35906E-05	C9JF17	APOD		
-0.68	1.71454E-05	2.5582E-05	A0A075B6S2	IGKV2D-29		
0.92	1.91816E-05	2.83948E-05	P35858	IGFALS	Hepatocytes	secreted
-0.93	2.5001E-05	3.67203E-05	F5GZZ9	CD163	Macrophages, DCs, NK cells	
0.46	2.70301E-05	3.93927E-05	P01011	SERPINA3	Hepatocytes	secreted
0.52	2.81495E-05	4.07085E-05	P02763	ORM1	Hepatocytes	secreted
-0.68	3.22567E-05	4.62921E-05	P04003	C4BPA	Hepatocytes	secreted
-0.71	5.02305E-05	7.15404E-05	P20851	C4BPB	Hepatocytes	

0.48	5.60156E-05	7.918E-05	P25311	AZGP1	Hepatocytes, Cholangiocytes	secreted
-0.85	0.000121418	0.000170347	P01861	IGHG4		
-0.46	0.000130694	0.000181514	P09871	C1S	Hepatocytes, Cholangiocytes	secreted
1.14	0.000131308	0.000181514	Q5VY30	RBP4	Hepatocytes, NK cells, Cholangiocytes	secreted
0.26	0.000224148	0.00030759	P01042	KNG1	Hepatocytes	secreted
-1.11	0.000251884	0.000343146	A0A0G2JMB2	IGHA2		
0.33	0.000404603	0.000547233	P02760	AMBP	Hepatocytes, NK cells, Cholangiocytes	secreted
0.44	0.000410727	0.000551548	P00748	F12	Hepatocytes, NK cells, Cholangiocytes	secreted
-0.34	0.000459537	0.000612716	O00391	QSOX1		
-0.43	0.000487019	0.000644785	P19320	VCAM1	Macrophages, DCs	
-0.31	0.000508999	0.000666491	A0A3B3ISJ1	PROS1	Hepatocytes	secreted
0.24	0.000510504	0.000666491	P08185	SERPINA6	Hepatocytes	secreted
-0.68	0.000762196	0.000988227	Q16610	ECM1	Endothelial cells	
-0.23	0.000854832	0.001100742	P01023	A2M	Hepatocytes, Endothelial cells	secreted
-0.47	0.000980736	0.001254275	A0A3B3ISR2	C1R	Hepatocytes, Cholangiocytes	secreted
0.41	0.001168048	0.001483737	P02743	APCS	Hepatocytes	secreted
0.28	0.001208231	0.00152448	G3XAM2	CFI	Hepatocytes	secreted
-0.18	0.002050403	0.002569838	P08603	CFH	Hepatocytes, NK cells, Cholangiocytes	secreted
0.26	0.002425278	0.003019551	P01031	C5	Hepatocytes	secreted
-0.18	0.005084798	0.006289092	P00450	CP	Hepatocytes, NK cells, Cholangiocytes	secreted
-0.31	0.005372673	0.006601716	E9PHK0	CLEC3B		
0.39	0.005736855	0.007003433	P15169	CPN1	Hepatocytes	secreted
0.24	0.00674552	0.008181663	Q14624	ITIH4	Hepatocytes	secreted
0.32	0.007420557	0.008942722	P02749	APOH	Hepatocytes, Cholangiocytes	secreted
-0.28	0.007579048	0.009075548	P10909	CLU	Hepatocytes, Cholangiocytes	secreted
0.36	0.010908239	0.012979423	P13796	LCP1	Macrophages, Neutrophils, B cells	
-0.35	0.015659771	0.018515955	Q96RL7	VPS13A	Hepatocytes, Cholangiocytes	
0.27	0.020448688	0.024027209	Q06033	ITIH3	Hepatocytes, NK cells, Cholangiocytes	secreted
0.32	0.022296758	0.026035966	P02751	FN1	Hepatocytes, Cholangiocytes	secreted
-0.35	0.024358174	0.028267511	Q9Y5Y7	LYVE1	Macrophages, DCs, Hepatocytes	intracellular
-0.49	0.025453121	0.029356973	P07996	THBS1	Monocytes	
-0.14	0.025835578	0.029616394	P04217	A1BG	Hepatocytes	secreted
0.14	0.028639902	0.032632131	P22792	CPN2	Hepatocytes	secreted

0.31	0.029072167	0.032925104	P19652	ORM2	Hepatocytes	secreted
-0.51	0.036775887	0.0414004	A0A0C4DH43	IGHV2-70D		
-0.15	0.06730001	0.075311916	B4E1Z4	CFB		
0.14	0.074023305	0.082345451	P36955	SERPINF1		
-0.18	0.077915835	0.086165747	P01024	C3		
0.13	0.0977423	0.107459371	P01009	SERPINA1		
0.12	0.108667783	0.118776414	P08697	SERPINF2		
-0.12	0.119733716	0.130115253	P02765	AHSG		
0.17	0.162521433	0.175597871	P04275	VWF		
-0.09	0.223740041	0.24036073	O75882	ATRN		
0.18	0.245023676	0.261729836	P08571	CD14		
0.22	0.262761486	0.279091295	P02750	LRG1		
0.11	0.269027241	0.284141131	P05160	F13B		
0.1	0.347830509	0.365319194	P00734	F2		
-0.07	0.353076075	0.368768345	A0A7I2V2D2	SERPING1		
0.13	0.405115072	0.420782505	P06727	APOA4		
0.12	0.414305035	0.427963443	A0A087WSY5	CPB2		
-0.06	0.550208959	0.565241991	P43652	AFM		
0.06	0.613433623	0.626769137	P02748	C9		
-0.02	0.829390302	0.842839875	A0A0C4DGB6	ALB		
-0.01	0.924335988	0.928631374	P51884	LUM		
0.01	0.926771277	0.928631374	P02787	TF		
0.01	0.928631374	0.928631374	O75636	FCN3		

Supp. Table 5. Univariable logistic regression analysis modeling the probability of the acute liver failure case being related to acetaminophen or not. Model parameters obtained in the discovery cohort were then assessed in the validation cohort. Performances were evaluated via the c-statistic and are depicted for both discovery and validation cohort. Depicted are the 10 best-performing variables from the discovery cohort.*95 % CI: 95 % confidence interval; ALDOB: fructose-bisphosphate aldolase B; CAT: catalase; PIGR: polymeric immunoglobulin receptor; CCL7: CC-chemokine ligand 7; CCL2: CC-chemokine ligand 2; ALT: alanine aminotransferase; AST: aspartate aminotransferase; CXCL11: C-X-C motif chemokine 11; IL15: interleukin 15.*

	Discovery cohort			Validation cohort
**Variable**	**estimate**	**p**	**C-statistic (95%CI)**	**C-statistic (95%CI)**
ALDOB	0.84	1.23E-12	0.93 (0.89 - 0.96)	0.92 (0.85 - 0.98)
CAT	0.61	4.75E-14	0.9 (0.85 - 0.94)	0.89 (0.83 - 0.95)
Bilirubin	-0.23	3.46E-11	0.86 (0.8 - 0.91)	0.93 (0.87-0.98)
PIGR	-0.69	7.69E-14	0.86 (0.81 - 0.91)	0.92 (0.87 - 0.97)
CCL7	0.61	1.47E-09	0.81 (0.75 - 0.88)	0.88 (0.81 - 0.95)
CCL2	0.97	4.17E-10	0.8 (0.73 - 0.87)	0.88 (0.81 - 0.95)
ALT	0	5.4E-09	0.8 (0.74 - 0.86)	0.87 (0.81 - 0.94)
AST	0	5.59E-07	0.8 (0.74 - 0.86)	0.84 (0.77 - 0.92)
CXCL11	-0.59	6.58E-08	0.76 (0.69 - 0.83)	0.80 (0.7 - 0.9)
IL15	1.11	4.76E-08	0.76 (0.69 - 0.83)	0.88 (0.82 - 0.95)

Supp. Table 6. Comparison of mean protein abundancies between individuals surviving the first 21 days post study admission (SpS) and those who either received liver transplantation or died (non-SpS) [discovery cohort]. A log fold change > 0 indicates proteins elevated in the non-SpS group. Data are sorted according to false discovery rate adjusted p-value (FDR). Features with an FDR < 0.05 were mapped to publicly available single cell RNAseq data (livercellatlas.org). For hepatocellular proteins, information on their behavior (i.e., secreted vs. intracellular) was added. *logFC: log fold change; p: p-value; FDR: false discovery adjusted p-value; uniprot: uniprot identifier; gene: gene symbol.*

logFC	p	FDR	uniprot	Gene	Celltypes	secreted/intracellular
-0.37	7.8199E-07	0.00013929	P01009	SERPINA1	Hepatocytes	secreted
-0.91	1.4818E-06	0.00013929	P02750	LRG1	Hepatocytes	secreted
1.02	4.4435E-06	0.00027846	F5GZZ9	CD163	Macrophages, DCs, NK cells	
-0.52	7.9972E-06	0.00037587	Q06033	ITIH3	Hepatocytes, NK cells, Cholangiocytes	secreted
-0.47	2.5298E-05	0.00095121	A0A7P0T8D1	AGT	Hepatocytes, Cholangiocytes	secreted
0.44	8.5759E-05	0.00237305	E9PHK0	CLEC3B		
-0.46	8.8358E-05	0.00237305	P02749	APOH	Hepatocytes, Cholangiocytes	secreted
-1.12	0.00019195	0.0045108	Q5VY30	RBP4	Hepatocytes, NK cells, Cholangiocytes	secreted
-0.32	0.00031575	0.00659572	Q14624	ITIH4	Hepatocytes	secreted
-0.54	0.00058487	0.01099556	P02774	GC	Hepatocytes, Cholangiocytes	secreted
-0.37	0.00085422	0.01433652	P01011	SERPINA3	Hepatocytes	secreted
-0.58	0.00098634	0.01433652	P0C0L5	C4B	Hepatocytes	secreted
-0.34	0.00116213	0.01433652	P10909	CLU	Hepatocytes, Cholangiocytes	secreted
-0.41	0.00120042	0.01433652	P07360	C8G	Hepatocytes	secreted
-0.23	0.00120374	0.01433652	A0A7I2V2D2	SERPING1	Hepatocytes, Endothelial cells	secreted
-0.67	0.00123469	0.01433652	P18428	LBP	Hepatocytes	secreted
0.37	0.00129639	0.01433652	P23142	FBLN1		
-0.2	0.00139622	0.01458271	P04217	A1BG	Hepatocytes	secreted
0.52	0.00161907	0.01602023	P55058	PLTP	Endothelial cells	
-0.2	0.00186173	0.01750026	P22792	CPN2	Hepatocytes	secreted
0.23	0.00214728	0.01922325	A0A0C4DGB6	ALB	Hepatocytes, NK cells, Cholangiocytes	secreted

-0.29	0.00261528	0.02234874	P19823	ITIH2	Hepatocytes	secreted
-0.31	0.00422282	0.03451695	P01008	SERPINC1	Hepatocytes	secreted
-0.23	0.00452496	0.0354455	B4E1Z4	CFB	Hepatocytes	secreted
0.4	0.0048291	0.03631486	A0A4W9A917	IGHG3		
0.48	0.00510648	0.03692378	A0A0A0MS15	IGHV3-49		
-0.34	0.00550674	0.03834323	P25311	AZGP1	Hepatocytes, Cholangiocytes	secreted
0.2	0.00599917	0.03866879	P02787	TF	Hepatocytes, NK cells, Cholangiocytes	secreted
-0.35	0.00603139	0.03866879	P02763	ORM1	Hepatocytes	secreted
-0.19	0.00617055	0.03866879	P01042	KNG1	Hepatocytes	secreted
-0.71	0.0070687	0.04224382	P04004	VTN	Hepatocytes	secreted
-0.36	0.00719044	0.04224382	P05546	SERPIND1	Hepatocytes	secreted
0.7	0.00768664	0.04336761	A0A0J9YY99	IGLD-2		
0.82	0.00784308	0.04336761	A0A0G2JMB2	IGHA2		
0.19	0.00821402	0.04347611	O75882	ATRN	Hepatocytes, Cholangiocytes	secreted
0.57	0.00832521	0.04347611	P01782	IGHV3-9		
-0.37	0.00919998	0.04674583	P15169	CPN1	Hepatocytes	secreted
-0.76	0.00951609	0.04675962	A0A096LPE2	SAA2-SAA4	Hepatocytes	secreted
-0.33	0.00970013	0.04675962	P02743	APCS	Hepatocytes	secreted
-0.48	0.01358963	0.06295411	P0C0L4	C4A		
-0.36	0.01372935	0.06295411	P07357	C8A		
-0.31	0.0142004	0.0635637	P02748	C9		
-0.27	0.01597505	0.0698444	P04196	HRG		
-0.21	0.01639168	0.07003716	P01031	C5		
0.21	0.01858396	0.07728511	A0A4W8ZXM2	IGHV3-72		
-0.29	0.01891019	0.07728511	P09871	C1S		
-0.59	0.02212988	0.08851951	V9GYM3	APOA2		
-0.32	0.02430794	0.09520609	P13671	C6		
0.38	0.02930183	0.11242334	P10643	C7		
0.47	0.03228122	0.11980822	P07996	THBS1		
0.44	0.03250116	0.11980822	Q16610	ECM1		
0.28	0.03739679	0.13520379	P02745	C1QA		
0.46	0.04037614	0.14322103	A0A0C4DH68	IGKV2-24		
0.21	0.04224971	0.14709159	P01859	IGHG2		

0.66	0.04467688	0.15271371	Q5H9A7	TIMP1		
-0.29	0.04690918	0.15748082	A0A3B3ISR2	C1R		
-0.3	0.04864209	0.15913517	A0A087WSY5	CPB2		
-0.17	0.04909489	0.15913517	G3XAM2	CFI		
-0.25	0.05367646	0.16858831	P07358	C8B		
-0.19	0.05423359	0.16858831	P01024	C3		
0.4	0.05470153	0.16858831	P04433	IGKV3D-11		
0.35	0.05836482	0.1769772	A0A0C4DH41	IGHV4-61		
-0.23	0.06302342	0.18806988	P02649	APOE		
0.54	0.06465498	0.18992401	A0A0C4DH34	IGHV4-28		
-0.14	0.07089632	0.20357591	P08697	SERPINF2		
0.21	0.07146814	0.20357591	P02747	C1QC		
-0.48	0.07584067	0.21280664	P02776	PF4		
0.17	0.07961959	0.22012474	P43652	AFM		
0.33	0.0812826	0.22052632	A0A0B4J1V1	IGHV3-21		
0.26	0.08312012	0.22052632	O43866	CD5L		
0.14	0.08328388	0.22052632	O75636	FCN3		
0.57	0.08527283	0.2206671	P04075	ALDOA		
-0.16	0.08568457	0.2206671	P02760	AMBP		
0.2	0.08837893	0.22379092	P04275	VWF		
0.17	0.08927829	0.22379092	P51884	LUM		
0.17	0.09068842	0.22433452	O00391	QSOX1		
0.31	0.09557456	0.23335087	Q15848	ADIPOQ		
0.25	0.09684265	0.23341562	P01876	IGHA1		
0.26	0.10108278	0.24055142	P01780	IGHV3-7		
0.17	0.10391714	0.24187525	A0A087X1J7	GPX3		
-0.57	0.10535087	0.24187525	P20618	PSMB1		
-0.3	0.1063067	0.24187525	P02647	APOA1		
0.22	0.10678535	0.24187525	Q96RL7	VPS13A		
-0.2	0.11738272	0.2627137	P00748	F12		
-0.32	0.12192153	0.26966174	A0A182DWH7	SEPP1		
0.31	0.12874937	0.28145211	H0Y755	FCGR3A		
-0.24	0.13112876	0.2833587	Q15582	TGFBI		

-0.61	0.15133104	0.32329812	P00738	HP		
0.57	0.15548106	0.32838562	P02675	FGB		
0.26	0.15809437	0.32838562	P01624	IGKV3OR2-268		
0.08	0.16018645	0.32838562	P08603	CFH		
0.17	0.16069934	0.32838562	A0A0A0MSV6	C1QB		
-0.15	0.17134231	0.34636941	P04114	APOB		
0.18	0.17688396	0.35376792	A0A0B4J1X5	IGHV3-74		
0.14	0.18015416	0.35651561	P05160	F13B		
0.31	0.18363394	0.3577491	P01703	IGLV1-40		
-0.26	0.18458331	0.3577491	P02775	PPBP		
0.17	0.18764554	0.35997308	P19320	VCAM1		
0.21	0.19232165	0.36521686	A0A075B6S2	IGKV2D-29		
-0.19	0.19669726	0.36979085	A0A087WT59	TTR		
-0.08	0.19923243	0.37084849	P00450	CP		
0.52	0.20354954	0.37516974	P17174	GOT1		
-0.18	0.20719949	0.37818937	P02751	FN1		
0.3	0.21477852	0.38825349	P01602	IGKV1-5		
0.18	0.22387551	0.39826349	P01871	IGHM		
0.31	0.2246096	0.39826349	P04432	IGKV1D-39		
-0.17	0.22667124	0.39826349	O95445	APOM		
0.29	0.23180689	0.40082592	A0A0C4DH43	IGHV2-70D		
0.2	0.23239375	0.40082592	P04003	C4BPA		
0.12	0.24305357	0.41540064	P01857	IGHG1		
0.2	0.25699331	0.43526795	I3L4N8	ACTG1		
0.16	0.26180888	0.4394649	P13796	LCP1		
-0.15	0.2657811	0.44064027	P80748	IGLV3-21		
-0.08	0.26719676	0.44064027	P08185	SERPINA6		
0.13	0.29141385	0.4763983	A0A0C4DH38	IGHV5-51		
0.07	0.29887577	0.48438487	P01023	A2M		
0.2	0.30588871	0.49044631	P01599	IGKV1-17		
-0.15	0.30783332	0.49044631	P19652	ORM2		
-0.16	0.31803648	0.50111188	P08571	CD14		
-0.27	0.31985865	0.50111188	P04211	IGLV7-43		

-0.43	0.32316158	0.50210229	P29401	TKT		
0.15	0.33906189	0.51927318	A0A0C4DH67	IGKV1-8		
-0.18	0.33973724	0.51927318	P02790	HPX		
-0.18	0.34662934	0.52553481	P27169	PON1		
0.4	0.35863341	0.53938465	P02679	FGG		
-0.07	0.43365059	0.64359362	P19827	ITIH1		
0.18	0.43476803	0.64359362	P01861	IGHG4		
0.07	0.44704205	0.65647986	P01834	IGKC		
0.25	0.45045693	0.65647986	P24298	GPT		
-0.37	0.45456331	0.65736847	P04040	CAT		
-0.1	0.45932843	0.65918889	Q96PD5	PGLYRP2		
0.29	0.46894791	0.66789551	P32754	HPD		
-0.13	0.47286029	0.66840401	P29622	SERPINA4		
-0.14	0.48275761	0.67730172	P01742	IGHV1-69		
0.09	0.49701184	0.69213501	P01717	IGLV3-25		
-0.13	0.50864305	0.70312422	Q9Y6R7	FCGBP		
0.05	0.53985092	0.73702258	P05543	SERPINA7		
0.26	0.54242449	0.73702258	P04424	ASL		
0.09	0.55131767	0.73702258	Q9Y5Y7	LYVE1		
-0.06	0.55487952	0.73702258	P43251	BTD		
0.21	0.55624817	0.73702258	P01833	PIGR		
-0.36	0.55668727	0.73702258	Q16851	UGP2		
-0.15	0.5639339	0.74139562	A0A0B4J1V2	IGHV2-26		
-0.1	0.58264322	0.7606731	P20851	C4BPB		
0.13	0.60720846	0.78277589	A0A0B4J1U7	IGHV6-1		
0.08	0.60790043	0.78277589	P01700	IGLV1-47		
-0.15	0.63916873	0.81744028	P28066	PSMA5		
0.11	0.65040836	0.82179017	P01701	IGLV1-51		
0.07	0.65131242	0.82179017	P06312	IGKV4-1		
-0.12	0.67856417	0.8504671	O14791	APOL1		
0.07	0.69390145	0.86393028	P01591	IGJ		
0.14	0.71549389	0.88403685	P04179	SOD2		
-0.09	0.71945552	0.88403685	P02671	FGA		

-0.12	0.7318639	0.89060063	G3V5Z7	PSMA6		
-0.07	0.73851347	0.89060063	P35858	IGFALS		
-0.06	0.73900904	0.89060063	H0YAC1	KLKB1		
-0.03	0.75133555	0.89968843	P02765	AHSG		
-0.06	0.75900258	0.90311699	A0A075B6K4	IGLV3-10		
0.04	0.7833226	0.92619276	P06727	APOA4		
-0.11	0.78949081	0.9276517	P04406	GAPDH		
-0.08	0.79810403	0.93194756	A0A024RA52	PSMA2		
0.04	0.81896726	0.94499354	P12955	PEPD		
-0.06	0.8193295	0.94499354	P00390	GSR		
0.09	0.8292555	0.94583996	P07099	EPHX1		
-0.11	0.83505861	0.94583996	P05062	ALDOB		
-0.08	0.83515656	0.94583996	P25788	PSMA3		
0.04	0.85177524	0.95732224	P00747	PLG		
-0.06	0.85705316	0.95732224	P16930	FAH		
0.09	0.86057159	0.95732224	Q7Z4W1	DCXR		
0.01	0.87364115	0.95841957	A0A3B3ISJ1	PROS1		
0.07	0.87530925	0.95841957	Q03154	ACY1		
0.02	0.88206901	0.95841957	P01619	IGKV3-20		
0.07	0.8895085	0.95841957	P07900	HSP90AA1		
-0.01	0.89130167	0.95841957	A0A0B4J231	IGLL5		
-0.02	0.8935229	0.95841957	Q08380	LGALS3BP		
0.02	0.89724385	0.95841957	P01714	IGLV3-19		
-0.04	0.90258152	0.95867415	P25786	PSMA1		
0.04	0.91346737	0.96478576	A0A7P0TAE1	HSP90B1		
-0.05	0.93344943	0.97228725	P00352	ALDH1A1		
-0.01	0.94001698	0.97228725	P36955	SERPINF1		
-0.01	0.94974333	0.97228725	P0DOY2	IGLC2		
0.01	0.95528569	0.97228725	P00734	F2		
0.02	0.95574294	0.97228725	H0YMZ1	PSMA4		
0.02	0.95981711	0.97228725	O14818	PSMA7		
-0.01	0.96140765	0.97228725	A0A0B4J1Y8	IGLV9-49		
0.02	0.96194377	0.97228725	P11021	HSPA5		

0	0.98136807	0.98661602	C9JF17	APOD		
0	0.99442401	0.99442401	P00739	HPR		

Supp. Table 7. Prediction of canonical pathways to be related to the signature of serum proteins associated with 21-day outcome [discovery cohort]. Presented are all pathways that are significantly associated with the observed proteomic changes (-log10 of the overlap p-value < 1.3).

Ingenuity Canonical Pathways	-log(p)	Associated proteins in dataset
**Acute Phase Response Signaling**	33.10	AGT, AHSG, ALB, APCS, C1QA, C1QC, C1R, C4A/C4B, C4BPA, CFB, CRP, FGA, FGB, FGG, HRG, ITIH2, ITIH3, ITIH4, LBP, RBP4, SERPINA1, SERPINA3, SERPINF2, SERPING1
**Complement System**	19.40	C1QA, C1QC, C1R, C4A/C4B, C4BPA, C6, C8B, CFB, CFH, CFI, SERPING1
**LXR/RXR Activation**	18.20	AGT, AHSG, ALB, APOA4, C4A/C4B, CD14, CLU, FGA, ITIH4, LBP, PLTP, RBP4, SERPINA1, SERPINF2
**FXR/RXR Activation**	14.70	AGT, AHSG, ALB, APOA4, C4A/C4B, CLU, FGA, ITIH4, PLTP, RBP4, SERPINA1, SERPINF2
**Coagulation System**	9.09	FGA, FGB, FGG, SERPINA1, SERPINC1, SERPINF2
**Extrinsic Prothrombin Activation Pathway**	6.90	FGA, FGB, FGG, SERPINC1
**IL-15 Signaling**	5.94	IGHA2, IGHG3, IGHV1-69, IGHV3-72, IGHV6-1, IGKC, IGKV2D-29, IGKV4-1
**Intrinsic Prothrombin Activation Pathway**	5.18	FGA, FGB, FGG, SERPINC1
**B Cell Receptor Signaling**	4.88	IGHA2, IGHG3, IGHV1-69, IGHV3-72, IGHV6-1, IGKC, IGKV2D-29, IGKV4-1
**Production of Nitric Oxide and Reactive Oxygen Species in Macrophages**	4.70	ALB, APOA4, CAT, CLU, RBP4, SERPINA1
**Atherosclerosis Signaling**	4.44	ALB, APOA4, CLU, RBP4, SERPINA1
**IL-12 Signaling and Production in Macrophages**	4.31	ALB, APOA4, CLU, RBP4, SERPINA1
**Systemic Lupus Erythematosus In B Cell Signaling Pathway**	4.27	IGHA2, IGHG3, IGHV1-69, IGHV3-72, IGHV6-1, IGKC, IGKV2D-29, IGKV4-1
**Communication between Innate and Adaptive Immune Cells**	4.18	IGHA2, IGHG3, IGHV1-69, IGHV3-72, IGHV6-1, IGKC, IGKV2D-29, IGKV4-1
**Clathrin-mediated Endocytosis Signaling**	3.59	ALB, APOA4, CLU, RBP4, SERPINA1
**Maturity Onset Diabetes of Young (MODY) Signaling**	2.80	ADIPOQ, ALDOB, APOA4
**Multiple Sclerosis Signaling Pathway**	2.45	C1QA, C1QC, C6, C8B
**LPS/IL-1 Mediated Inhibition of RXR Function**	2.37	CAT, CD14, LBP, PLTP
**Neuroprotective Role of THOP1 in Alzheimer's Disease**	2.34	AGT, C1R, SERPINA3

**Role of Tissue Factor in Cancer**	2.31	FGA, FGB, FGG
**GP6 Signaling Pathway**	2.22	FGA, FGB, FGG
**IL-6 Signaling**	2.18	CD14, CRP, LBP
**iNOS Signaling**	2.08	CD14, LBP
**Primary Immunodeficiency Signaling**	2.00	IGHG3, IGKC
**Tyrosine Degradation I**	1.83	FAH
**Pathogen Induced Cytokine Storm Signaling Pathway**	1.73	CRP, FGA, FGB, FGG
**Hepatic Fibrosis / Hepatic Stellate Cell Activation**	1.72	AGT, CD14, LBP
**IL-10 Signaling**	1.71	CD14, LBP
**Macropinocytosis Signaling**	1.67	CD14, CSF1R
**Toll-like Receptor Signaling**	1.66	CD14, LBP
**Sucrose Degradation V (Mammalian)**	1.63	ALDOB
**Superoxide Radicals Degradation**	1.63	CAT
**LPS-stimulated MAPK Signaling**	1.58	CD14, LBP
**Apelin Adipocyte Signaling Pathway**	1.57	CAT, GPX3
**Systemic Lupus Erythematosus Signaling**	1.46	C6, C8B, IGHG3, IGKC
**Actin Cytoskeleton Signaling**	1.46	CD14, GSN, LBP

Supp. Table 8. Prediction of upstream regulators to be associated with signature of serum proteins associated with 21-day outcome [discovery cohort]. Presented are all upstream regulators that are significantly associated with the observed proteomic changes (-log10 of the overlap p-value < 1.3).

**Upstream Regulator**	**-log(p)**	**target proteins in dataset**
**HNF1A**	10.9281	ALB, APOH, CPB2, CRP, ITIH4, SERPINA1, SERPING1, VTN
**IL6**	7.2055	CD163, CRP, LBP, ORM1, SERPINA3
**ALB**	4.4056	AGT, ALB
**SORL1**	3.4486	AGT, AZGP1, FBLN1, PLTP
**RUNX1**	3.1798	ALB, CSF1R
**Hsp27**	3.1349	CD163, CSF1R
**GLIS1**	2.8729	EFEMP1, FBLN1
**CFH**	2.6968	CRP
**HP**	2.3958	CD163
**DAB2IP**	2.0958	CLU
**TCF**	2.0232	SERPINA1, SERPINA3
**IL17A**	1.9914	CD163, CRP
**Mmp**	1.9208	CSF1R
**TNF**	1.8928	AGT, CD163, CFB, CRP
**IFNG**	1.8729	AGT, CD163, CSF1R
**HFE**	1.8539	TF
**HSF1**	1.8327	EFEMP1, FBLN1

**TREX1**	1.8239	CFB, GSN
**STAT1**	1.7799	C1R, C4A/C4B
**TGFB1**	1.7545	ALB, CD163, SERPINA1
**CTNNB1**	1.7144	SERPINA1, SERPINA3
**Hemoglobin**	1.7011	CD163
**Pde4**	1.6596	CD163
**CG**	1.6478	C1R, C4A/C4B, CFB
**TNFSF11**	1.5884	CSF1R
**TXN**	1.5884	C5
**FOXA2**	1.5560	ALB
**IL1**	1.5258	CRP
**LEP**	1.5258	CRP
**C1Q (family)**	1.4989	CD163
**ATF3**	1.4989	GSN
**ARID1A**	1.4855	C4A/C4B, FBLN1
**EPO**	1.3830	TF
**PPARA**	1.3635	RBP4
**mir-155**	1.3261	CD163
**CSF1**	1.3089	CD163

Supp. Table 9. Comparison of mean cytokine levels between individuals surviving the first 21 days post study admission and those either receiving liver transplantation or passing away [discovery cohort]. Data are sorted according to false discovery rate adjusted p-value (FDR). *logFC: fold change of the log2-transformed cytokine abundancies.*

LogFC	p	FDR	uniprot ID	gene symbol
-1.6250	1.09E-05	4.89E-04	P05231	IL6
-0.6207	3.12E-05	7.01E-04	P14210	HGF
-1.2563	1.80E-04	2.71E-03	P09919	CSF3
-1.0899	4.99E-04	5.62E-03	P22301	IL10
-0.8949	2.40E-03	2.16E-02	Q9P0M4	IL17C
0.7356	5.40E-03	4.05E-02	P01133	EGF
-0.6874	1.06E-02	6.81E-02	P04141	CSF2
0.3593	2.38E-02	1.34E-01	P13232	IL7
-0.4287	2.87E-02	1.44E-01	Q96PD4	IL17F
-0.3049	3.99E-02	1.48E-01	P40933	IL15
0.5482	4.21E-02	1.48E-01	O14625	CXCL11
-0.4438	4.35E-02	1.48E-01	Q07325	CXCL9
-0.4413	4.37E-02	1.48E-01	P01584	IL1B
-0.4656	4.59E-02	1.48E-01	Q969D9	TSLP
-0.1802	6.31E-02	1.86E-01	O43508	TNFSF12
-0.3930	6.60E-02	1.86E-01	P10145	CXCL8
-0.4629	7.66E-02	2.03E-01	P39900	MMP12

-0.2524	1.02E-01	2.43E-01	P13236	CCL4
0.1496	1.03E-01	2.43E-01	P50591	TNFSF10
-0.2772	1.35E-01	2.89E-01	P10147	CCL3
-0.2286	1.35E-01	2.89E-01	O95760	IL33
-0.2453	1.44E-01	2.95E-01	P13725	OSM
-0.2101	1.84E-01	3.46E-01	Q99616	CCL13
-0.3958	1.92E-01	3.46E-01	P01579	IFNG
-0.2939	1.92E-01	3.46E-01	Q16552	IL17A
-0.1398	2.60E-01	4.50E-01	P78380	OLR1
-0.2097	2.81E-01	4.68E-01	P60568	IL2
-0.2038	3.14E-01	5.04E-01	P02778	CXCL10
-0.1657	3.32E-01	5.14E-01	P80075	CCL8
-0.0464	3.61E-01	5.14E-01	P09603	CSF1
-0.2955	3.65E-01	5.14E-01	P80098	CCL7
-0.1521	3.65E-01	5.14E-01	Q14116	IL18
-0.1113	3.81E-01	5.20E-01	P03956	MMP1
-0.0910	4.13E-01	5.46E-01	P51671	CCL11
-0.1318	4.92E-01	6.32E-01	P13500	CCL2
0.0665	6.20E-01	7.74E-01	P15692	VEGFA
-0.0393	6.88E-01	8.37E-01	P05112	IL4
0.0480	7.50E-01	8.59E-01	P01375	TNF
-0.0624	7.59E-01	8.59E-01	Q99731	CCL19
0.0538	7.64E-01	8.59E-01	P01135	TGFA
0.0331	7.89E-01	8.66E-01	P49771	FLT3LG
-0.0314	8.56E-01	9.17E-01	P48061	CXCL12
0.0224	9.05E-01	9.47E-01	P35225	IL13
0.0086	9.47E-01	9.62E-01	P01374	LTA
0.0108	9.62E-01	9.62E-01	Q8NEV9_Q14213	IL27

Supp. Table 10. Univariable logistic regression analysis modeling the probability of death or transplant within 21 days. Depicted are the three best-performing proteomic variables, several features derived from the cytokine profiling as well as routine clinical parameters. Model parameters obtained in the discovery cohort were then assessed in the validation cohort. Performances were evaluated via the c-statistic and are depicted for both discovery and validation cohort. *95 % CI: 95 % confidence interval; SERPINA1: alpha1 antitrypsin; MELD: model for end-stage liver disease; LRG1: leucine-rich alpha-2-glycoprotein; IL6: interleukin 6; AGT: angiotensinogen; HGF: hepatocyte growth factor; CSF3: colony stimulating factor 3; IL10: interleukin 10.*

	Discovery cohort			Validation cohort
**Variable**	**estimate**	**p**	**C-statistic (95%CI)**	**C-statistic (95%CI)**
SERPINA1	1.54	4.5E-06	0.71 (0.64 - 0.78)	0.76 (0.67 - 0.84)
MELD	-0.1	5.43E-06	0.71 (0.63 - 0.78)	0.74 (0.65 - 0.83)
LRG1	0.57	9.65E-06	0.71 (0.64 - 0.78)	0.68 (0.58 - 0.78)
IL6	-0.28	4.37E-05	0.67 (0.59 - 0.75)	0.62 (0.51 - 0.73)
AGT	0.84	7.49E-05	0.67 (0.59 - 0.74)	0.68 (0.59 - 0.78)
HGF	-0.71	8.81E-05	0.66 (0.58 - 0.74)	0.7 (0.6 - 0.81)
CSF3	-0.27	0.00047	0.65 (0.57 - 0.73)	0.57 (0.47 - 0.68)
IL10	-0.26	0.00092	0.64 (0.56 - 0.72)	0.45 (0.34 - 0.56)
Bilirubin	-0.03	0.061	0.58 (0.5 - 0.66)	0.72 (0.63 - 0.81)
Creatinine	-0.01	0.88	0.53 (0.45 - 0.61)	0.55 (0.44 - 0.65)

Supp.Table 11: Results of the multivariable logistic regression analysis combining the ALFSG-prognostic index (ALFSG-PI) and single proteomic features to model the probability of death or transplant within 21 days. For each model, results are presented via their respective areas under the receiver operating curve (AUROCs) in the discovery and validation cohort, as well as the difference between the two. Models numerically outperforming ALFSG-PI in both cohorts are highlighted. *SERPINA1: alpha1 antitrypsin; LRG1: leucine rich alpha-2-glycoprotein 1; IL6: interleukin 6; AGT: angiotensinogen; HGF: hepatocyte growth factor; CSF3: colony stimulating factor 3; IL10: interleukin 10.*

model	AUC (discovery)	AUC (validation)	AUC (validation) – AUC (discovery)
**ALFSG-PI**	0.7465	0.746	-0.0005
**SERPINA1 + ALFSG-PI**	0.7681	0.8001	0.032
**LRG1 + ALFSG-PI**	0.7839	0.7475	-0.0364
**IL6 + ALFSG-PI**	0.7418	0.7345	-0.0073
**AGT + ALFSG-PI**	0.78	0.7659	-0.0141
**HGF + ALFSG-PI**	0.7478	0.7633	0.0155
**CSF3 + ALFSG-PI**	0.7512	0.7331	-0.0181
**IL10 + ALFSG-PI**	0.7544	0.7328	-0.0217

Supp. Table 12. Composition of the selected prognostic models (modeling probability of death or liver transplant within 21 days). *SERPINA1: alpha1 antitrypsin; IL6: interleukin 6; EGF: epidermal growth factor; ATRN: attractin; INR: international normalized ratio; HE grade: degree of hepatic encephalopathy; VIF: variable inflation factor.*

	estimate	standard error	z value	p	VIF
**Model 1**					
(Intercept)	-10.4835	3.8682	-2.7102	0.0067	
**SERPINA1**	1.6242	0.4188	3.8783	0.0001	1.0642
**IL6**	-0.3735	0.0955	-3.9098	0.0001	1.2924
**EGF**	0.2892	0.128	2.2599	0.0238	1.2775
**ATRN**	-0.8711	0.4413	-1.9737	0.0484	1.086
**Bilirubin**	-0.0719	0.0218	-3.2987	0.001	1.2573
**Model 2**					
	**estimate**	**standard error**	**z value**	**p**	**VIF**
(Intercept)	-8.8775	4.0165	-2.2102	0.0271	
**SERPINA1**	1.0815	0.4454	2.428	0.0152	1.0566
**INR**	-0.4147	0.1275	-3.2539	0.0011	1.1953
**need for ventilation (yes)**	-1.7583	0.467	-3.7654	0.0002	1.3317
**EGF**	0.4016	0.1387	2.8964	0.0038	1.263
**Bilirubin**	-0.1041	0.0263	-3.9631	0.0001	1.6811
**ALFSG-PI**					
	**estimate**	**standard error**	**z value**	**p**	**VIF**
(Intercept)	3.9343	0.8916	4.4127	0.00001	
**HE grade (3/4)**	-1.2381	0.3691	-3.3547	0.0008	1.1145
**favorable etiology (yes)**	-0.0944	0.4571	-0.2065	0.8364	1.7045
**need for vasopressor therapy (yes)**	-0.3198	0.4656	-0.6867	0.4923	1.1647
**ln(Bilirubin)**	-0.6274	0.2465	-2.5448	0.0109	1.6779
**ln(INR)**	-1.5322	0.3733	-4.1042	0.00004	1.0696

Supplementary Figures

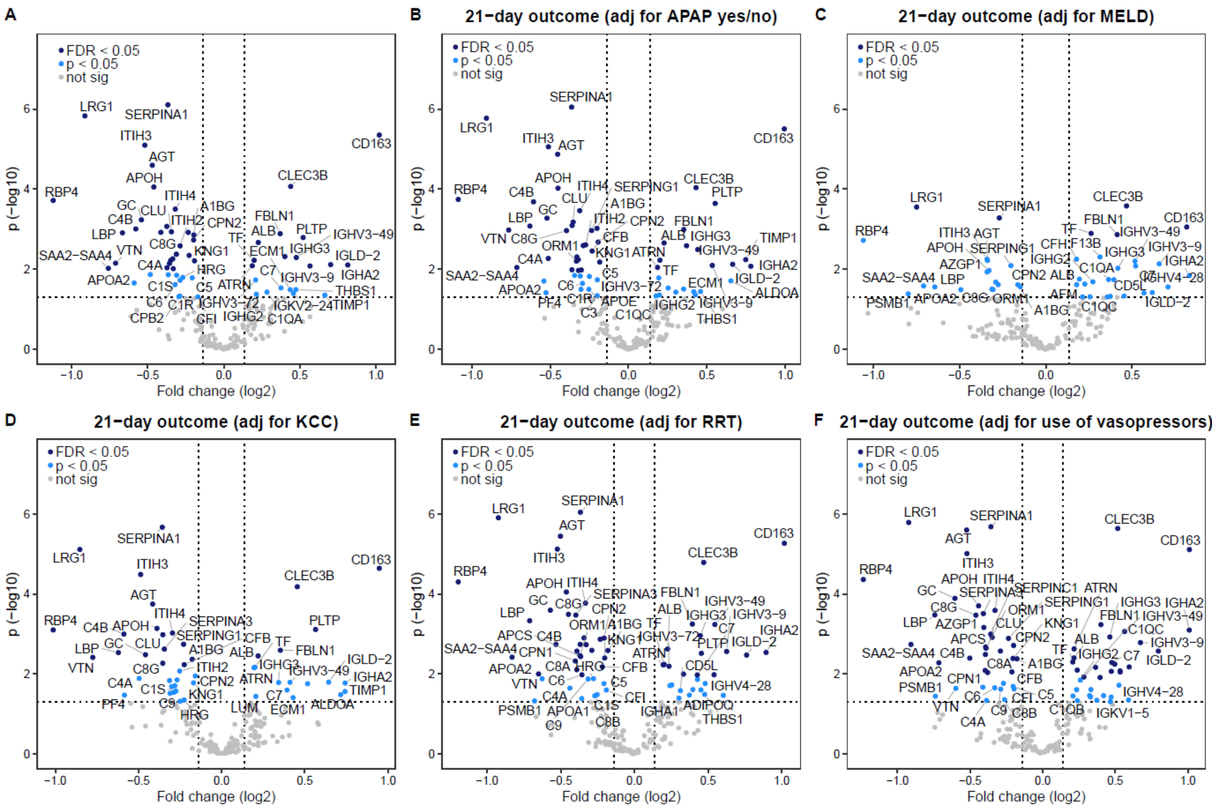

Supp. Fig. 1: Adjustment of 21-day outcome differential abundance analysis for different parameters of disease progression/severity. Volcano plots depicting proteins that are differentially abundant between spontaneous survivors and non-survivors are presented in the original form as stated in Figure 3 of the main manuscript (no covariates added) [A] and after the addition of covariates representing either disease etiology (APAP yes/no) [B] or surrogates of disease severity (MELD [C], King's College Criteria [KCC] [D], need for renal replacement therapy [RRT] [E] or need for the use of vasopressors [F].

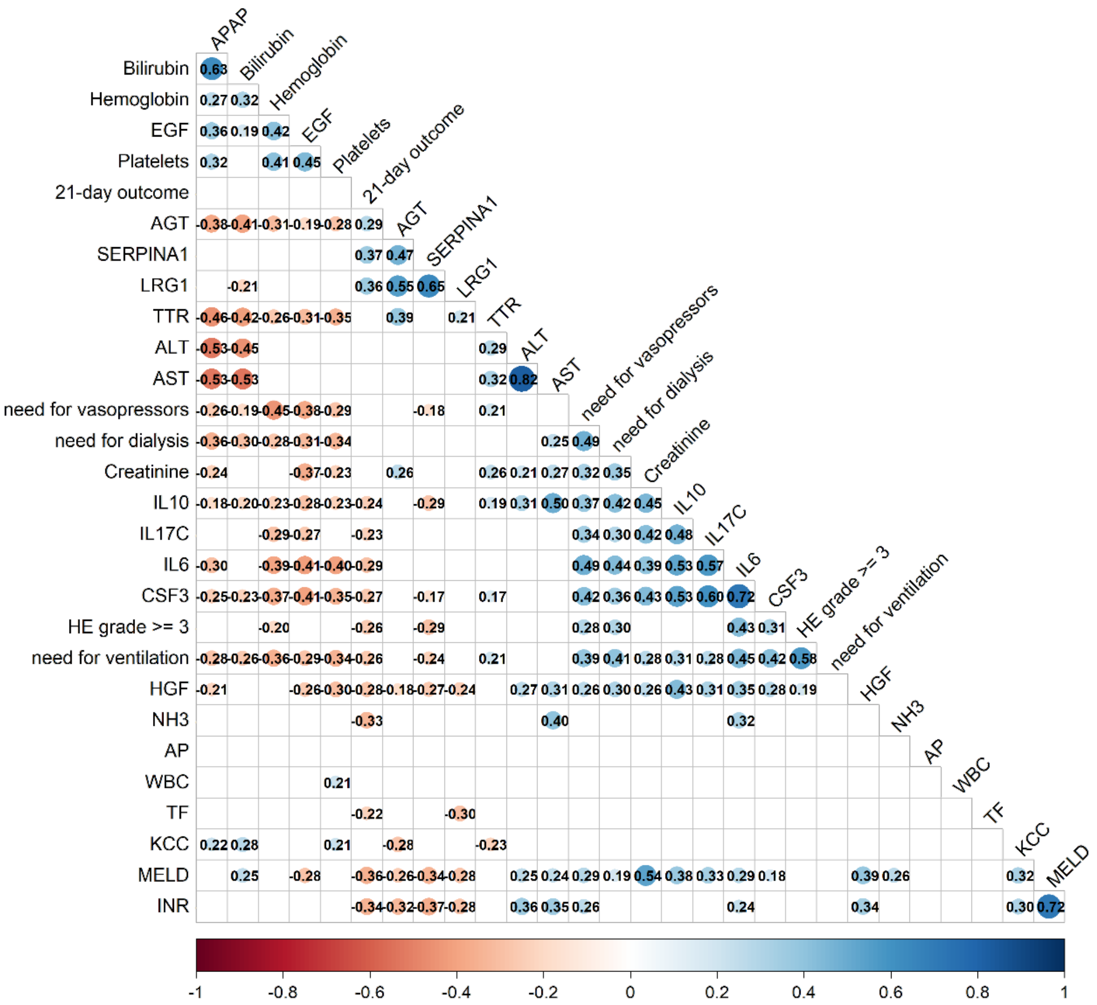

Supp. Fig. 2. Correlation between selected parameters in the discovery cohort. Spearman rank coefficients that remained statistically significant after correction for multiple testing via false-discovery rate adjustment (FDR < 0.05) are displayed. *EGF: epidermal growth factor; 21-day outcome: spontaneous survival vs liver transplantation or death during first 21 days post study admission; AGT: angiotensinogen; SERPINA1: alpha1-antitrypsin; LRG1: leucine-rich alpha-2-glycoprotein; TTR: transthyretin; ALT: alanine aminotransferase; AST: aspartate aminotransferase; IL10: interleukin 10; IL17C: interleukin 17C, IL6: interleukin 6; CSF3: colony-stimulating factor 3; HE grade >= 3: degree of hepatic encephalopathy >= 3; HGF: hepatocyte growth factor; NH3: venous ammonia levels; AP: alkaline phosphatase; WBC: white blood cell count; TF: transferrin; KCC: fulfilling King's College Criteria yes/no; MELD: model for end-stage liver disease; INR: international normalized ratio.*

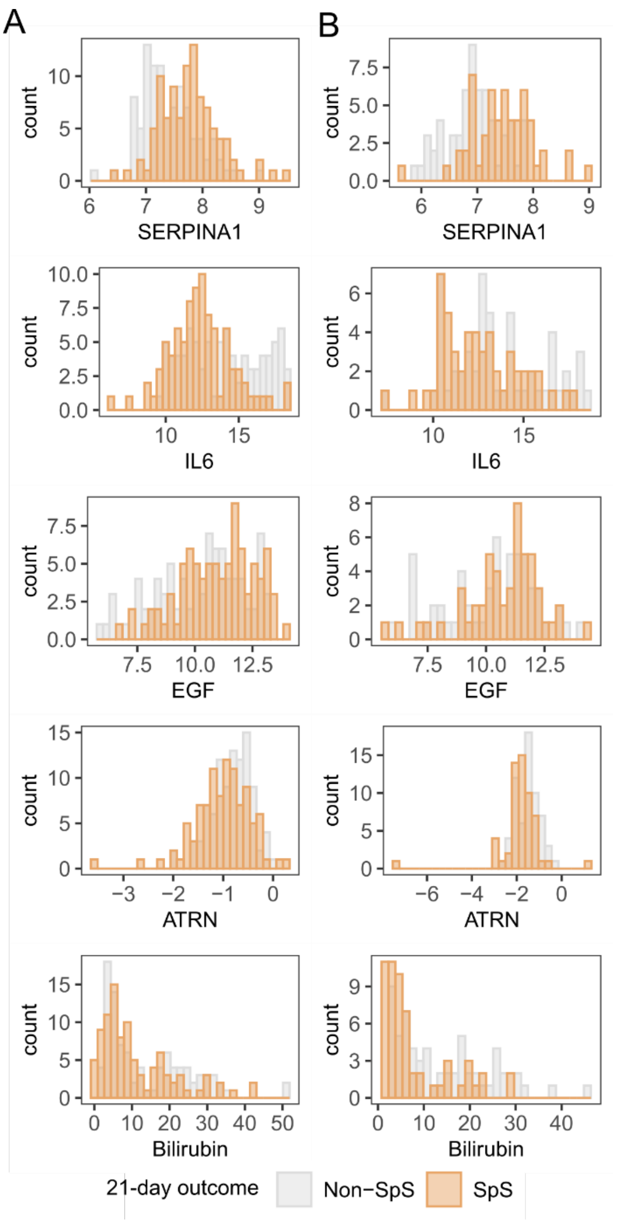

Supp. Fig. 3. Distribution of continuous parameters assessed in multivariable logistic regression. Histograms displaying the distribution of continuous parameters included in models 1 and 2 in the discovery [A] and validation [B] cohort. *SERPINA1: alpha1 antitrypsin; IL6: interleukin 6; EGF: epidermal growth factor; ATRN: attractin.*

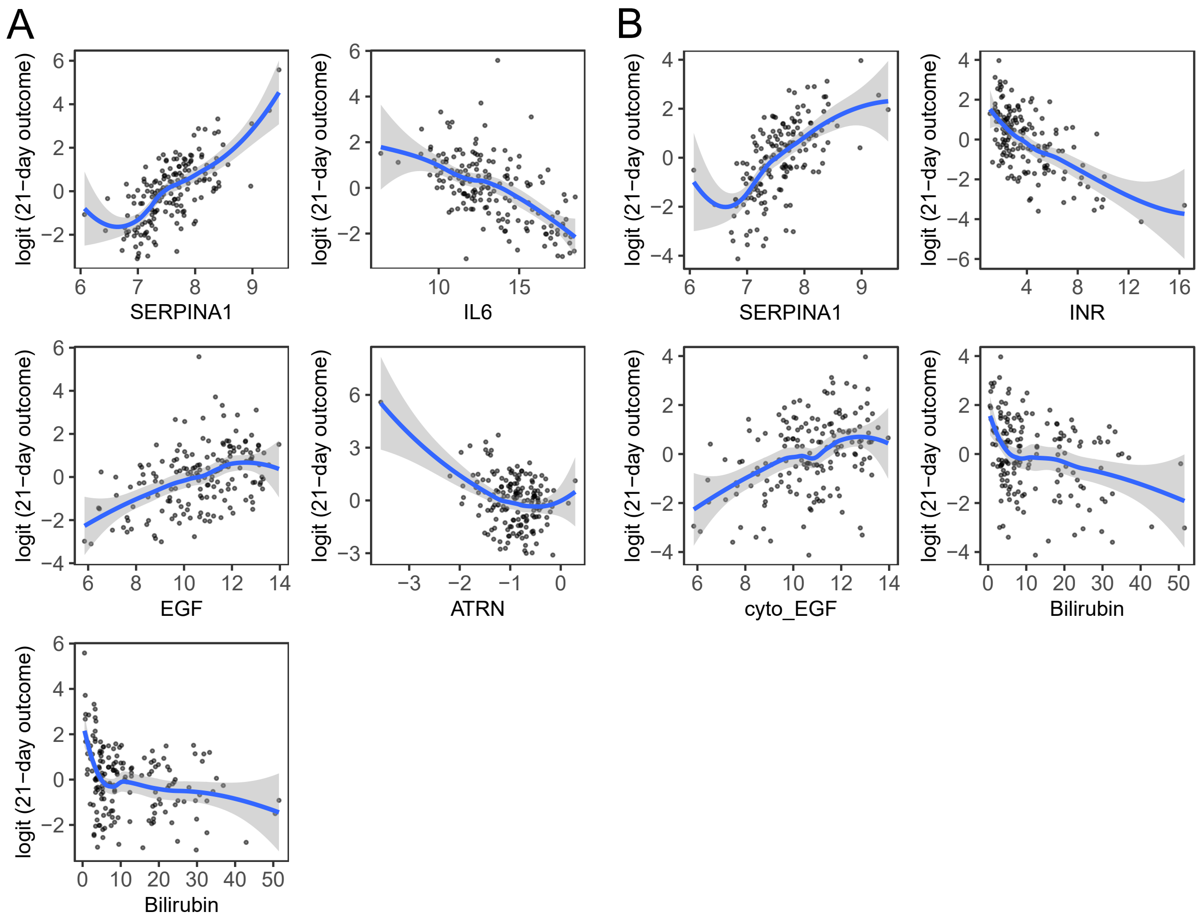

Supp. Fig. 4. Assessment of relationship between continuous modeling parameters and logit of the 21-day outcome. Plots visualizing the relationship between the continuous parameters chosen for models 1 and 2 and the logit of the 21-day outcome [A: model 1, B: model 2]. *SERPINA1: alpha1 antitrypsin; IL6: interleukin 6; EGF: epidermal growth factor; ATRN: attractin; INR: international normalized ratio.*

Supplementary References

1 **Wang Z, Karkossa I**, Großkopf H, *et al.* Comparison of quantitation methods in proteomics to define relevant toxicological information on AhR activation of HepG2 cells by BaP. *Toxicology*. 2021;448. doi: 10.1016/j.tox.2020.152652

2 **Schmidt JR, Geurtzen K**, von Bergen M, *et al.* Glucocorticoid Treatment Leads to Aberrant Ion and Macromolecular Transport in Regenerating Zebrafish Fins. *Front Endocrinol (Lausanne)*. 2019;10:1–17.

3 **Guilliams M, Bonnardel J**, Haest B, *et al.* Spatial proteogenomics reveals distinct and evolutionarily conserved hepatic macrophage niches. *Cell*. 2022;185:379-396.e38.

4 **Guilliams M, Bonnardel J**, Haest B, *et al.* [dataset] Spatial proteogenomics reveals distinct and evolutionarily-conserved hepatic macrophage niches (single cells). *Gene Expr Omnibus*. 2022;GSE192740.

Author names in bold designate shared co-first authorship.
